# A collection of bacterial isolates from the pig intestine reveals functional and taxonomic diversity

**DOI:** 10.1038/s41467-020-19929-w

**Published:** 2020-12-15

**Authors:** David Wylensek, Thomas C. A. Hitch, Thomas Riedel, Afrizal Afrizal, Neeraj Kumar, Esther Wortmann, Tianzhe Liu, Saravanan Devendran, Till R. Lesker, Sara B. Hernández, Viktoria Heine, Eva M. Buhl, Paul M. D’Agostino, Fabio Cumbo, Thomas Fischöder, Marzena Wyschkon, Torey Looft, Valeria R. Parreira, Birte Abt, Heidi L. Doden, Lindsey Ly, João M. P. Alves, Markus Reichlin, Krzysztof Flisikowski, Laura Navarro Suarez, Anthony P. Neumann, Garret Suen, Tomas de Wouters, Sascha Rohn, Ilias Lagkouvardos, Emma Allen-Vercoe, Cathrin Spröer, Boyke Bunk, Anja J. Taverne-Thiele, Marcel Giesbers, Jerry M. Wells, Klaus Neuhaus, Angelika Schnieke, Felipe Cava, Nicola Segata, Lothar Elling, Till Strowig, Jason M. Ridlon, Tobias A. M. Gulder, Jörg Overmann, Thomas Clavel

**Affiliations:** 1 Functional Microbiome Research Group, RWTH University Hospital, Aachen, Germany; 2 Leibniz Institute DSMZ-German Collection of Microorganisms and Cell Cultures, Braunschweig, Germany; 3 German Center for Infection Research (DZIF), Partner site Hannover-Braunschweig, Braunschweig, Germany; 4 ZIEL - Institute for Food & Health, Technical University of Munich, Freising, Germany; 5 Chair of Technical Biochemistry, Technical University of Dresden, Dresden, Germany; 6 Microbiome Metabolic Engineering Theme, Carl R. Woese Institute for Genomic Biology, Urbana, IL USA; 7 Department of Animal Sciences, University of Illinois at Urbana-Champaign, Urbana, IL USA; 8 Department of Microbial Immune Regulation, Helmholtz Centre for Infection Research, Braunschweig, Germany; 9 Laboratory for Molecular Infection Medicine Sweden (MIMS), Department of Molecular Biology, Umeå University, Umeå, Sweden; 10 Laboratory for Biomaterials, Institute for Biotechnology and Helmholtz-Institute for Biomedical Engineering, RWTH Aachen University, Aachen, Germany; 11 Electron Microscopy Facility, Institute of Pathology, RWTH University Hospital, Aachen, Germany; 12 Department CIBIO, University of Trento, Trento, Italy; 13 National Animal Disease Center, Agricultural Research Service, United States Department of Agriculture, Ames, IA USA; 14 Department of Molecular and Cellular Biology, University of Guelph, Guelph, Canada; 15 Department of Parasitology, Institute of Biomedical Sciences, University of São Paulo, São Paulo, Brazil; 16 PharmaBiome AG, Zurich, Switzerland; 17 Chair of Livestock Biotechnology, Weihenstephan School of Life Science, Technical University of Munich, Freising, Germany; 18 Institute of Food Chemistry, Hamburg School of Food Science, University of Hamburg, Hamburg, Germany; 19 Department of Bacteriology, University of Wisconsin-Madison, Madison, WI USA; 20 Institute of Marine Biology, Biotechnology and Aquaculture, Hellenic Center of Marine Research, Heraklion, Greece; 21 Host-Microbe Interactomics Group, Department of Animal Science, Wageningen University, Wageningen, The Netherlands; 22 Electron Microscopy Center, Wageningen University, Wageningen, The Netherlands; 23 Hannover Medical School, Hannover, Germany; 24 Present Address: Institute of Food Technolgy and Food Chemistry, Technische Universität Berlin, Berlin, Germany

**Keywords:** Genomics, Phylogeny, Bacterial genomics, Metagenomics, Microbiome

## Abstract

Our knowledge about the gut microbiota of pigs is still scarce, despite the importance of these animals for biomedical research and agriculture. Here, we present a collection of cultured bacteria from the pig gut, including 110 species across 40 families and nine phyla. We provide taxonomic descriptions for 22 novel species and 16 genera. Meta-analysis of 16S rRNA amplicon sequence data and metagenome-assembled genomes reveal prevalent and pig-specific species within *Lactobacillus*, *Streptococcus*, *Clostridium*, *Desulfovibrio*, *Enterococcus*, *Fusobacterium*, and several new genera described in this study. Potentially interesting functions discovered in these organisms include a fucosyltransferase encoded in the genome of the novel species *Clostridium porci*, and prevalent gene clusters for biosynthesis of sactipeptide-like peptides. Many strains deconjugate primary bile acids in in vitro assays, and a *Clostridium scindens* strain produces secondary bile acids via dehydroxylation. In addition, cells of the novel species *Bullifex porci* are coccoidal or spherical under the culture conditions tested, in contrast with the usual helical shape of other members of the family *Spirochaetaceae*. The strain collection, called ‘Pig intestinal bacterial collection’ (PiBAC), is publicly available at www.dsmz.de/pibac and opens new avenues for functional studies of the pig gut microbiota.

## Abstract (web summary)

The authors present a public collection of 117 bacterial isolates from the pig gut, including the description of 38 novel taxa. Interesting functions discovered in these organisms include a new fucosyltransferease and sactipeptide-like molecules encoded by biosynthetic gene clusters.

## Introduction

Pigs are invaluable animals in biomedical research. The physiology of their digestive tract and omnivorous feeding behaviour make them ideal models for studying gastrointestinal and metabolic pathologies where mice hold limited value^1–3^. For instance, transgenic pig models of colorectal cancer^4^ and diabetes^5^ help generating useful preclinical data on molecular mechanisms underlying disease. Pigs are also very important agricultural domestic animals, with an estimated worldwide population nearing one billion individuals. Both biomedical research and agriculture would benefit from enhanced understanding of the pig gut microbiome. Intestinal microorganisms are known to regulate the onset of chronic diseases such as cancer^6–8^ and metabolic disorders^9–11^. They also influence animal growth^12,13^ and play an important role in resistance against enteric infections^14,15^. However, underlying mechanisms remain poorly described and microbiome-based applications are hampered due to our still limited knowledge about gut microbiota members^16,17^.

Sequencing studies have provided detailed insights into the pig gut microbiome, including a reference catalogue of ~8 million microbial genes using stool from almost 300 pigs^18^. In agreement with other reports^16,19,20^, this study highlights the necessity to assess gut microbiomes in a host-specific manner considering the narrow overlap in terms of gene diversity and the occurrence of bacterial taxa between host species, despite the high similarity of functional potential between ecosystems. The study by Xiao et al.^18^. also underlines the urgent need to describe novel bacteria, considering that <10% of the genes detected could be taxonomically assigned at the genus level. The renaissance of cultivation has already generated valuable data pertaining to the human and mouse gut microbiota^19,21,22^. In contrast, there has so far been no comprehensive study of cultured bacteria from the pig intestine, despite obvious added value: providing access to well-described bacterial strains will facilitate (i) functional studies to dissect microbe–host interactions underlying diseases in preclinical contexts, and (ii) intervention trials with defined cocktails of commensals as an alternative to antibiotics use. Such trials could particularly influence enteric infection-associated post-weaning diarrhoea that affect millions of pigs worldwide and is linked to the use of colistin, a last-resort antibiotic in human medicine^23,24^.

In this work, we aimed to establish the pig intestinal bacterial collection (PiBAC), a publicly available repository of cultured commensal strains from the pig intestine. This includes the formal description of 38 novel bacterial taxa complemented by metagenomic investigations. A detailed survey of the ecological distribution of all taxa as well as new functional insights are presented.

## Results and discussion

### PiBAC: a resource of cultured bacteria from the pig gut

**Fig. 1:**
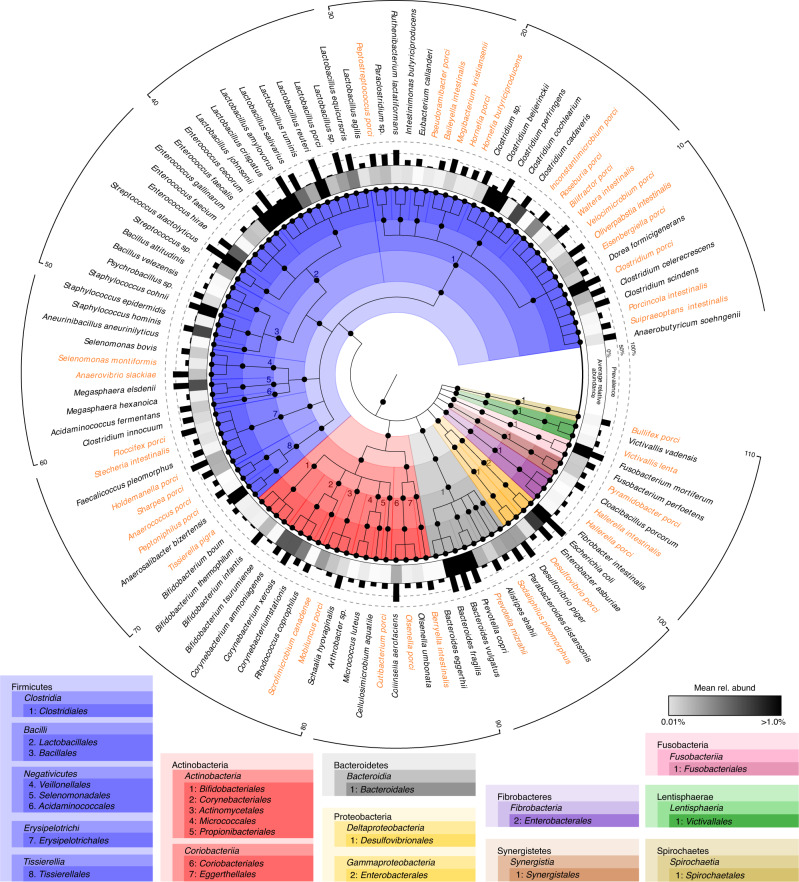
Species within the pig intestinal bacterial collection (PiBAC). Cladogram depicting the taxonomic classification of all 110 species. The colour code is according to phyla and the lineage of bacteria is given in boxes below the cladogram. Novel taxa (their candidate names) appear in orange letters. The outer black bars represent the prevalence of each species as determined by 16S rRNA gene search against 1346 pig gut-derived amplicon datasets (see Methods). The grey gradient below indicates the mean relative abundances of each isolate in the samples that were positive for the given species.

Cultivation of bacteria from the pig intestine has been sporadic, focusing mainly on pathogens or probiotic strains, with little effort placed on commensals. Hence, we isolated ~1100 bacterial pure cultures from the pig gut. A total of 117 strains representing 110 species across 40 families and nine phyla were selected based on MALDI-biotyping and 16S rRNA gene sequences to provide maximal species-level coverage, with the addition of multiple strains for six species due to different growth and metabolic features or origins of isolation. The diversity and occurrence in the pig intestine of the 110 selected species is summarized in Fig. 1 and a listing with detailed metadata (e.g. origin, accession numbers, genome features, culture conditions, etc.) is provided in Supplementary Data 1. A phylogenomic tree of all strains can be seen in Supplementary Fig. 1. Most species within the collection (*n* = 91) were detected in 16S rRNA gene amplicon data from 1346 samples at a prevalence >10%, indicating their presence in dominant communities within pig gut microbiota. Seventeen species had a prevalence >50% and a relative abundance >0.5%, suggesting that they are key members of the pig gut microbiome (Fig. 1 and Supplementary Data 1). Genomes were generated for all 117 strains (including two closed genomes) and were used for taxonomic description, functional profiling including the occurrence of antimicrobial resistance genes (Supplementary Fig. 2), biomolecule identification, and prediction of minimal bacterial consortia (see sections below). All strains were deposited at the German Collection of Microorganisms and Cell Cultures (DSMZ) to ensure long-term preservation and public accessibility. A specific list linked to the metadatabase Bac*Dive*^25^ was created to allow rapid query of the collection by users (www.dsmz.de/pibac). To help researchers looking for pig gut bacteria besides our own isolates, this online list also contains 31 type strains isolated previously by others from the intestine of pigs and available at the DSMZ (see Supplementary Data 1).

### Novel diversity within the collection

Thirty-eight strains represented novel taxa based on MALDI profiles, 16S rRNA gene analysis, and genome-based taxonomic assignment. Cellular fatty acids and the production of short-chain fatty acids (SCFAs) were also determined (see protologues and Supplementary Fig. 3). Based on these results, the creation of 22 novel species and 16 novel genera is proposed to accommodate these isolates shown in orange letters in Fig. 1 and listed in colour in Supplementary Data 1. Their taxonomic description, including genome-based readouts and proposals for names, are provided at the end of the methods section. Note that, as in many other cases and despite relying on up-to-date parameters to delineate bacterial taxa, these descriptions are based on single strains, hence reclassifications may occur in the future once additional strains have been isolated^26^. 16S rRNA gene-based trees showing the phylogenetic placement of each novel taxon along with corresponding microscopic images are provided in Supplementary Fig. 4. Electron micrographs were generated for *Pseudoramibacter porci*, *Stecheria intestinalis*, and *Tissierella pigra* as these isolates presented interesting cell morphologies (Supplementary Figs. 5–7).

**Fig. 2:**
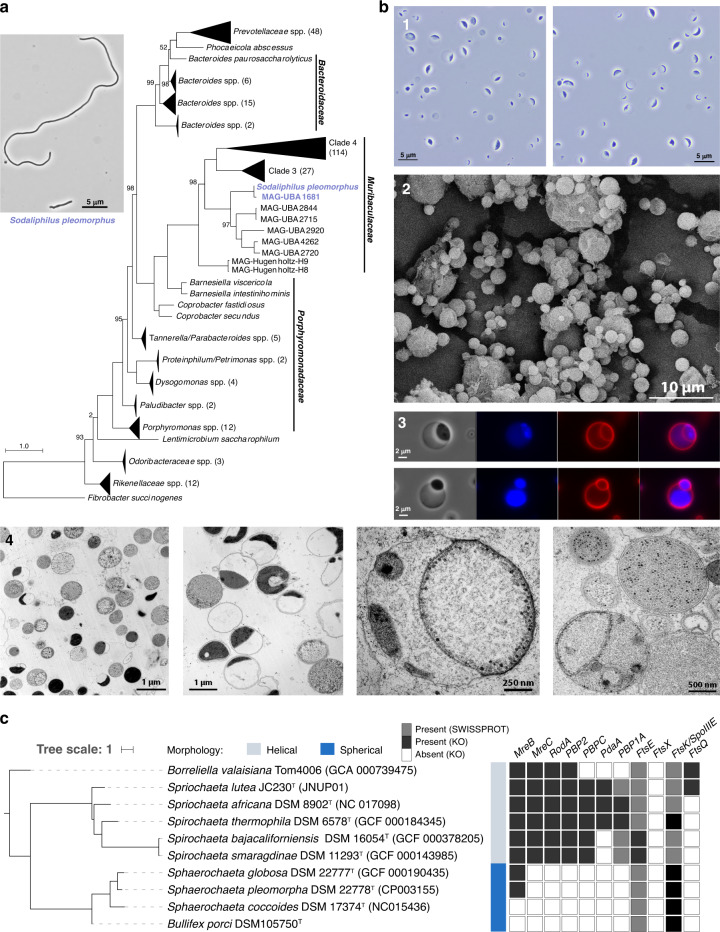
Novel diversity within the collection. **a** Phylogenomic tree of *Sodaliphilus pleomorphus* within members of the order *Bacteroidales* (phylum Bacteroidetes) together with a phase contrast micrograph of the strain grown on WCA agar with 5% sheep blood for 7 days at 37 °C under anaerobic conditions. **b** Microscopic investigations of *Bullifex porci* DSM 105750^T^. Picture 1 represent cells in their own growth medium observed immediately after removal from the anaerobic culture tube. The image was obtained using a N-Achroplan objective (100×/1,25 Oil Ph3 M27) mounted on an Axio Lab.A1 microscope equipped with an Axiocam 105 camera (Zeiss, Jena, Germany). Picture 2 is a scanning electron micrograph obtained as detailed in the methods section. Panel 3 shows cells stained with FM4-64 (red; membrane) and DAPI (blue; DNA) next to the same cells observed by phase contrast (left-hand side). Panel 4 shows cells imaged by transmission electron microscopy using 1.5% (first two pictures) or 0.2% (last two pictures) glutaraldehyde fixation. Multiple other images of *B. porci* obtained using a variety of sample preparations and microscopy techniques are available in Supplementary Figs. 8–10. **c** Phylogenomic tree showing the placement of *B. porci* among closest relatives within the family *Spirochaetaceae* (phylum Spirochaetes) together with the presence (black or grey boxes) or absence (white boxes) of genes involved in cell morphology and division, peptidoglycan synthesis, and cell wall formation (the entire set of genes tested is shown in Supplementary Fig. 2). *Sphaerochaeta associata* was not included in the analysis as no genome is yet available for this species. KO: KEGG orthology.

Among the 38 novel taxa, *Sodaliphilus pleomorphus* is the first pig isolate within the newly described family *Muribaculaceae*^16^ (phylum Bacteroidetes, order *Bacteroidales*). Both 16S rRNA gene-based phylogeny (Supplementary Fig. 4d) and phylogenomic analysis (Fig. 2a) showed the separate genus status of *Sodaliphilus*, which grows very slowly under the strictly anaerobic conditions tested and can form long filamentous cells (Fig. 2a). Another interesting isolate, *Bullifex porci*, represents a novel genus within the family *Spirochaetaceae*, phylum Spirochaetes. Whilst members of this phylum are usually helically shaped, the isolate showed a coccoidal to spherical cell morphology (Fig. 2b). Multiple micrographs are available in Supplementary Figs. 8–10. Growth was observed in media with varying osmolarities without observable change in the spherical cell shape (Supplementary Fig. 9). As four previously published species within the genus *Sphaerochaeta* were reported to have similar traits (three of which having genomes available)^27–29^, we searched the genomes of *B. porci* and *Spirochaetaceae* for genes involved in cell morphology and division, peptidoglycan synthesis, and cell wall formation (Supplementary Fig. 2a)^30–32^. Both the *rodA* and *mreC* genes were absent within *Sphaerochaeta* spp. and *Bullifex*: RodA is a peptidoglycan polymerase that, when knocked out, can lead to loss of control over cell elongation and body diameter^33^, whilst the cell shape protein MreC is key to rod-type cells formation (Fig. 2c)^34^. Interestingly, only *B. porci* and *Sphaerochaeta coccoides* lacked the *mreB* gene involved in cytoskeleton ring formation leading to cell rigidity^35^. Among the conserved proteins forming the divisome (or septal ring) involved in bacterial binary fission^30^, *ftsQ* and *ftsX* were absent in *B. porci* (Fig. 2c and Supplementary Fig. 2a). Both genes were reported to be also absent within the genomes of Planctomycetes species with unusual modes of cell division^32^. Regarding peptidoglycan biosynthesis, whilst the *murA-G*, *murJ*, and *mraY* genes were present within all studied genomes, homologous genes to the penicillin-binding proteins 1a, 2, and C were absent from *Sphaerochaeta* spp. and *B. porci* (Fig. 2c). Analysis of peptidoglycans detected the presence of murotetrapeptides containing L-Orn at third position of the peptide chain linked to a glycine (Supplementary Fig. 11). Altogether, these investigations highlight the particular cell structure of *B. porci*. Further studies are needed to dissect the cellular biology of this novel species (e.g. its mechanisms of cell division).

### Towards new bacterial functions

Genome-based mapping of the collection on KEGG pathways is shown in Supplementary Fig. 12. As antibiotics are a matter of concern in pig farming, data on the occurrence of antimicrobial resistance genes are summarized in Supplementary Data 1 and Supplementary Fig. 2b. The overall EggNOG-derived functional profile of all isolates indicated that nearly 25% of annotated proteins have unknown functions (Supplementary Fig. 2c), stressing the need to perform in-depth functional studies on cultured microbes. We therefore sought to highlight novel functions within PiBAC.

**Fig. 3:**
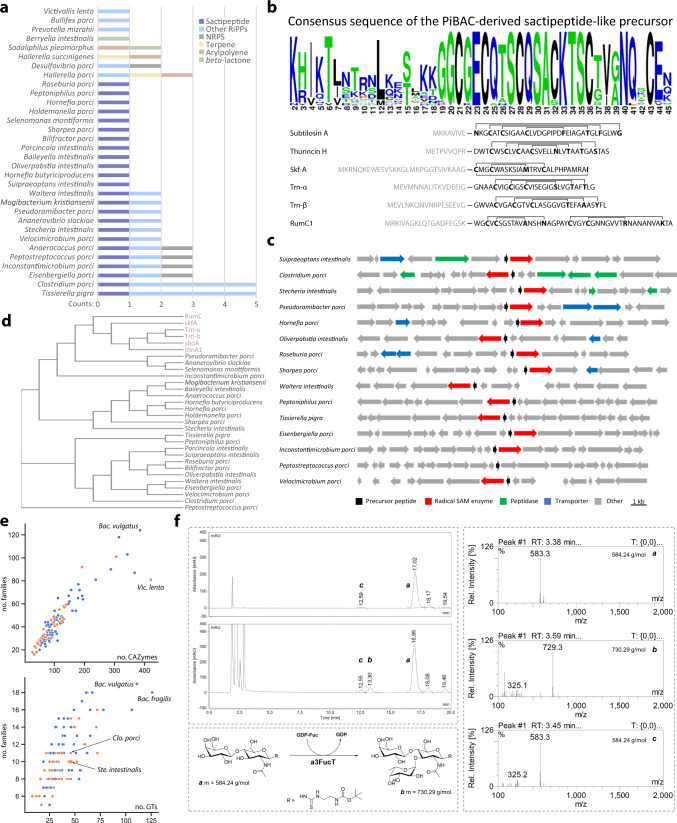
New bacterial functions. **a** Number of biosynthetic gene clusters (BGCs) identified within the novel taxa. NRPS non-ribosomal peptide synthetase, RiPP ribosomally synthesized and post-translationally modified peptides. **b** Top: consensus amino acid sequence of the PiBAC-derived sactipeptide-like BGCs with high sequence similarity in the precursor peptide sequence. Bottom: amino acid sequences of currently known sactipeptide natural products. Grey letters indicate amino acids of the leader peptides cleaved off in final products. Lines between bold amino acid residues indicate cyclization in the mature peptide. **c** Comparison of genetic organizations flanking the putative sactipeptide-like BGCs exemplarily shown for 15 of the pig strains. **d** Phylogenetic tree comparing amino acid sequences of known sactipeptide precursors (top six entries in red) and those from the pig bacteria. **e** Number of single CAZymes against CAZymes familes (top) and glycosyltransferases (GT) against GT families (bottom) for each member of the collection (*n* = 117 genomes representing 110 species depicted as dots). All data are provided in Supplementary Data 1. Orange dots indicate the 38 novel taxa while blue dots represent known bacterial species. Bacterial names correspond to species with the highest numbers of single enzymes or enzyme families. The position of *Clostridium porci* and *Stecheria intestinalis* is also shown (bottom), as these species encoded a GT of family 10 (along with *Bacteroides fragilis*). **f** Reaction of the new FucT from *C. porci* with *N*-acetyllactosamine. Top left box: HPLC measurements of the reaction without (negative control; top chromatogram) or with the co-substrate GDP-fucose (bottom). Relevant peaks (compounds) are named with letters: *a*, substrate (LacNAc type 2); *b*, target product; *c*, unidentified product with the same mass as the substrate and proposed to be iso-LacNAc, an isomer of LacNAc with a different bond between galactose and GlcNAc leading to a shift in retention time; likely originates from remnant transglycosidase activity in the enzyme preparation as also observed in the negative control. Right box: mass spectra of the relevant HPLC peaks. Bottom left box: putative reaction pathway catalyzed by *C. porci*.

Due to the importance of specialized bacterial metabolites for microbe–microbe and microbe–host interactions^36^, we bioinformatically analysed the genomes of all 38 novel taxa for the presence of biosynthetic gene clusters (BGCs). Sixty-four putative BGCs were identified in 32 of the 38 strains, encoding a broad variety of biosynthetic systems for natural products such as terpenes, arylpolyones, β-lactones, non-ribosomal peptides (NRPs), and ribosomally synthesized and post-translationally modified peptides (RiPPs) (Fig. 3a). Whereas the overall number of BGCs in these novel taxa was rather low for prokaryotic organisms, there was a striking enrichment for sactipeptide-like BGCs (24 of the 32 positive strains) compared with publicly available genomes (<1% prevalence in bacteria after BLAST search against the NCBI nr database using the prototypical SCIFF sequence motif mentioned below). Sactipeptides form a specific class of RiPPs with characteristic intramolecular sulfur-to-*alpha* carbon thioether bonds. The C-terminal amino acid sequence of the corresponding precursor peptides was highly conserved regardless of the phylogeny of isolates: all possessed the SCIFF (six cysteines in 45 residues) sequence motif initially defined in clostridia^37^. However, the sequences were significantly different from precursor peptides of previously characterized sactipeptide-encoding BGCs for subtilosin A^38^, thurincin H^39^, sporulation killing factor A (Skf-A)^40^, thuricin CD (Trn-*alpha* and -*beta*)^41^, and ruminococcin (RumC)^42^ (Fig. 3b). Despite the high homology of the precursor peptides (especially between positions 19–36), the overall genetic organization in proximity of the putative sactipeptide BGCs differed markedly across isolates (Fig. 3e), which precludes simple horizontal gene transfer between species. Phylogenetic analysis of the precursor peptides revealed a close relationship among all previously characterized metabolites whilst the new sequences identified in this study were clearly separated (Fig. 3d). The antibacterial potential of known sactipeptides^38–42^ and the widespread occurrence of unique, sactipeptide-like BGCs in our isolates suggest an important role of the encoded molecules in shaping the composition of pig gut microbiomes, which can now be investigated.

We then assessed carbohydrate-active enzymes (CAZymes) considering their importance for both ecological interactions in the gut and biotechnological applications^16,43,44^. Annotation of genomes against the CAZy database (www.cazy.org) revealed that all isolates encoded CAZymes, with a diversity of up to 421 enzymes in the novel species *Victivalis lenta* compared with an average number of 106 ± 67 for all other species (Fig. 3d). When looking specifically at glycosyltransferases (GTs), *Bacteroides fragilis* encoded the highest number (*n* = 126). This and two other species encoded a GT of family 10, which includes *alpha*-(1,3)-fucosyltransferases (FucT) that are of particular biotechnological interest^45^ (Fig. 3d). One of these isolates was the novel species *Clostridium porci*, a prevalent dweller of the pig intestine (see next section). Its putative FucT contained the FxxxFEN motif reported as conserved within *alpha*-(1,3)-FucT^46^. Structural modelling identified the highest structural similarity to 2NZW (identity, 30.8%; coverage, 84.8%), a known *alpha*-(1,3)-FucT. Gene ontology analysis via I-TASSER predicted the highest scoring molecular function as ‘fucosyltransferase activity’ (GO:0008417, score = 0.37). Heterologous expression and enrichment of this protein from *C. porci* allowed in vitro testing. The substrate LacNAc type 2 (peak a in Fig. 3f) was converted to a product with the molecular mass of fucosylated LacNAc (Lewis X) according to mass spectrometry (peak b in Fig. 3f). A fucosidase assay confirmed this product as a Lewis X epitope (fucose attached to the GlcNAc moiety of LacNAc in an *alpha*-3-glycosidic bond). Whilst detailed biochemistry of this enzyme requires additional work, its identification is a further proof of the functional bacterial diversity present within our strain collection.

### The ecology of PiBAC strains reveals host specificity

**Fig. 4:**
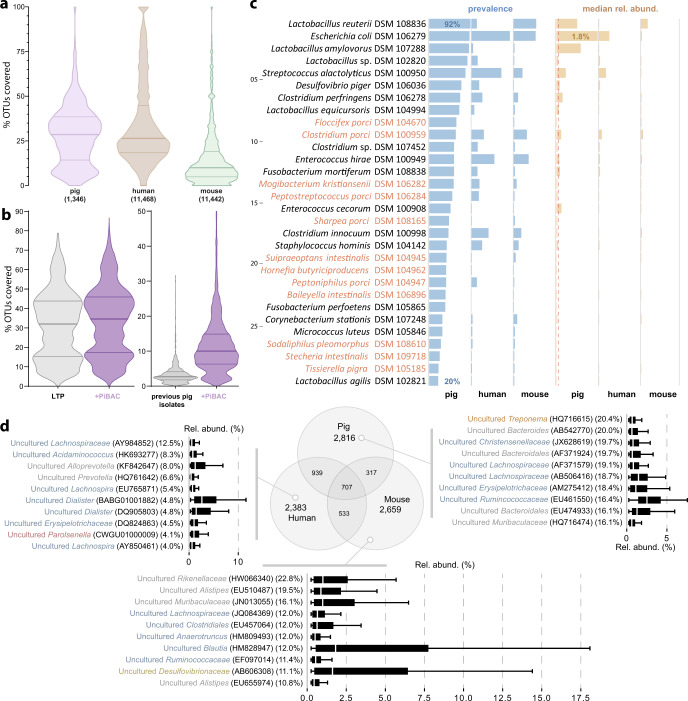
Host-specificity revealed by meta-analyses of 16S rRNA amplicons. **a** Percentage of IMNGS-derived^47^ 16S rRNA amplicon reads from the pig, mouse, and human intestine (numbers of samples analysed indicated in brackets) covered by sequences from all isolates. Horizontal lines indicate the median (middle), 25% (bottom), and 75% (top) quantiles. **b** Coverage of the 1346 pig gut samples by the 16S rRNA gene sequences from 13,903 isolates available via the Living Tree Project (LTP)^48^ or from 31 strains isolated from the pig intestine and available from strain collections prior to the present work with (violet) or without (grey) addition of the 38 novel taxa in PiBAC. **c** List of the 30 isolates with significantly increased prevalence and relative abundances in the pig intestine. The number of samples considered were as in **a**. Novel taxa are written in orange letters. The dashed line indicates 0.1% median relative abundance. **d** Overlap of 16S rRNA amplicon-based molecular species between the three host species (pig, human, mouse). The number of samples considered are as in **a**. The Venn diagram shows OTU numbers common between or unique to the respective host. The box plots display the relative abundance of the respective top-10 prevalent, host-specific OTUs (their prevalence is in brackets; coloured according to phyla as in Fig. 1). The box plots indicate the median (middle line), 25% and 75% quantiles (bottom and top line, respectively), and 1.5 × inter-quantile range after Tukey. For analyses in **c** and **d**, mouse and human data were randomly sub-sampled to obtain a number comparable to pigs (*n* = 1346 samples).

We collected all pig, mouse, and human gut-derived 16S rRNA amplicon datasets available in IMNGS^47^ with >5000 sequences (*n* = 1346, 11,442 and 11,468, respectively). Sequence mapping to the PiBAC species at 97% identity revealed a median cultured fraction of 35.8% in the pig gut vs. 24.4% and 10.5% in human and mouse, respectively (Kruskal–Wallis test; *p* < 0.001) (Fig. 4a). Further analysis showed study-specific cultured fractions that varied depending on diet, age, and gut locations with marked inter-individual differences (Supplementary Fig. 13). The 38 novel taxa accounted for a modest but significant increase in the median cultured fraction of pig microbiomes (+2.6%) relative to the entire diversity of isolates available via the Living Tree Project^48^ (*n* = 13,903) (Fig. 4b). The contribution of PiBAC was higher (+7.3%) relative to pig-derived isolates available via international collections before the present work (see listing in Supplementary Data 1) (Fig. 4b).

Thirty species in the collection had increased prevalence and relative abundances in the pig intestine compared with mice and humans, indicating host preference (see data for all collection members in Supplementary Data 1). These included four new taxa described in the present study with prevalence >50% across the 1346 samples tested, albeit at low relative abundances: *Floccifex porci*, *Clostridium porci*, *Mogibacterium kristiansenii*, and *Peptostreptococcus porci* (Fig. 4c). Host specificity was confirmed using all operational taxonomic units (OTUs) (*n* = 10,354) identified in the thousands of amplicon datasets. OTU mapping (97% identity) revealed only a small overlap (6.8%) between the three host species (Fig. 4d). OTUs that were unique to a given host were uncultured and characterized by prevalence ≤20%, which may reflect islands of diversity depending on the population considered (e.g. pig herd).

**Fig. 5:**
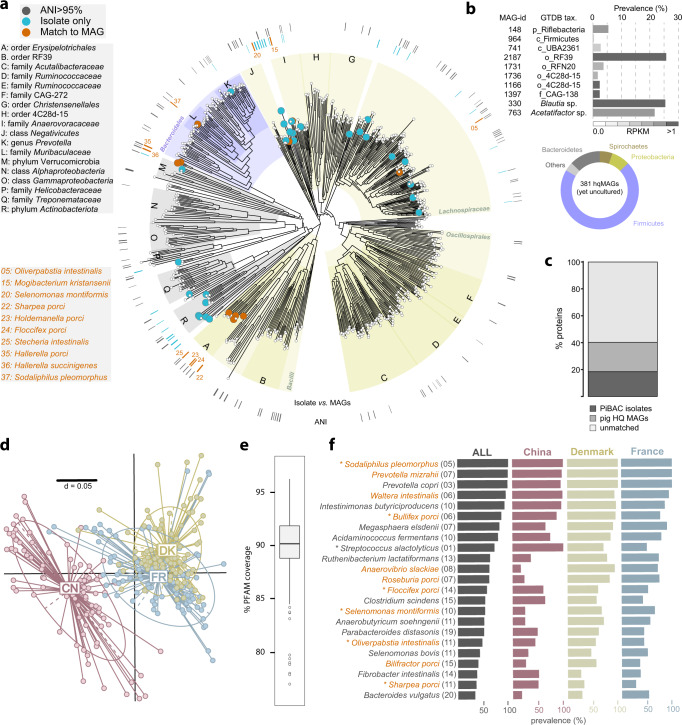
Metagenome-based diversity and functional prediction. **a** Phylogenomic tree of high-quality, bacterial metagenome-assembled genomes (MAGs) together with novel taxa within the collection. See text for tree construction and quality thresholds. The grey bars in the outer ring indicate genomes with a species-level match to MAGs archived in GTDB (ANI value >95%)^53^, no matter whether cultured or not. The colours in the inner ring indicate species-level match between the new PiBAC taxa and the MAGs catalogue. **b** Top: list of most-wanted taxa captured by metagenomics but without a cultured representative. Taxa were selected because they represent high taxonomic ranks and based on high prevalence or abundance. Bottom: coloured ring representing the phylum distribution of all 381 yet uncultured species represented by a high-quality (hq) MAG from the present study. **c** Contribution of the isolates and hqMAGs collected in the present study to the pig-derived metagenomic gene catalogue (7,685,872 proteins)^18^. **d** Jaccard similarity plot of PFAM-based metagenomic profiles of 284 pig faecal samples according to their country of origin: CN, China; Fr, France; DK, Denmark. **e** Functional coverage of the metagenomes in **d** (*n* = 284) by all species-level PiBAC genomes. The box plot indicates the median (middle line), 25% and 75% quantiles (bottom and top line, respectively), and 1.5 × inter-quantile range; outliers are indicated with dots. **f** List of the 23 species most often selected (>50% pigs in at least one country) within minimal communities (20 species on average) best matching the PFAM profiles of faecal pig metagenomes. The bar plots show the prevalence of each bacterial species across the entire cohort and in each country. The species marked with a star were identified as being enriched in pigs based on meta-amplicon or MAGs analysis (Figs. 4c and 5a). The numbers in bracket indicate the median selection rank of individual species across all minimal communities in which they were present. Novel taxa are written in orange letters.

The amplicon-based findings above were complemented by metagenomic analysis. A total of 589 high-quality (>90% completeness, <5% contamination)^49^ metagenome-assembled genomes (hqMAGs) were reconstructed from previously published pig gut metagenomes^18^ using a newly described workflow^50^ (see ‘Methods’ section) (Fig. 5a). These hqMAGs are available at https://github.com/strowig-lab/PIBAC. Ten of the 38 isolates representing new cultured taxa had a match ≥95% to MAGs, suggesting that they represent dominant bacterial members within pig microbiomes as detected by shotgun sequencing (Fig. 5a and Supplementary Data 2): *Holdemanella porci* (prevalence, 18% of the 295 pig metagenomes; mean relative abundance, 0.16 ± 0.07%), *Floccifex porci* (13%; 0.14 ± 0.05%), *Hallerella porci* (5%; 0.27 ± 0.30%), *Hallerella succinigenes* (27%; 0.21 ± 0.22%), *Mogibacterium kristiansenii* (12%; 0.13 ± 0.03%), *Oliverpabstia intestinalis* (99%; 0.23 ± 0.80%), *Selenomonas montiformis* (25%; 0.38 ± 0.42%), *Sharpea porci* (1%; 0.28 ± 0.21%), *Sodaliphilus pleomorphus* (99%; 0.31 ± 0.26%), *Stecheria intestinalis* (3%; 0.44 ± 0.42%).

We then assessed the overlap of the 38 novel taxa with the catalogue of human-associated bacteria compiled by extensive metagenomic assembly on more than 9500 human metagenomes^51^. Genomes were assigned to species-level genome bins (SGBs) at average nucleotide identity (ANI) values >95% as described in the original work cited above and implemented in an updated version of PhyloPhlAn^52^. Seventeen of the 38 genomes matched human SGBs, with ten having no cultured representative, confirming them to be unexplored even in the human microbiome (Supplementary Data 2). The other seven species had been isolated at least once previously but have so far not been validated and thus still represent cultured taxa newly described here. Most of these 17 pig strains matching a human SGB are members of the phylum Firmicutes (*n* = 11), three of which comprised >100 MAGs (*Holdemanella porci, Oliverpabstia intestinalis*, and *Waltera intestinalis*) and can thus be considered as prevalent gut bacterial species in human. More than half of the strains representing new taxa in the collection (*n* = 21) had however no match to the human SGBs catalogue and may thus represent pig-specific species (Supplementary Data 2).

Comparison to the Genome Taxonomy Database (GTDB)^53^ (Supplementary Data 2) and up-to-date repositories of MAGs from the mouse and human gut^50,51^ showed that most pig-derived hqMAGs (*n* = 381; 65%) represented unknown taxa (ANI values <95%). This highlights the novel richness within this metagenome-based species catalogue and the utility to further explore pig microbiomes by cultivation. Most of these yet-uncultured bacteria belong to the phylum Firmicutes and a list of ten ‘most-wanted’ taxa is provided (Fig. 5b). Searching for sequences from the original pig gene catalogue (7,685,872 proteins)^18^ assignable to a genome revealed that PiBAC isolates accounted for 18.4% of the proteins with the inclusion of hqMAGs increasing this by 21.8%, providing a total coverage of 40.2% (Fig. 5c). Moreover, a further 69,360 proteins from the isolates and 114,643 proteins from hqMAGs could not be assigned to proteins in the existing catalogue, suggesting that the created resources not only account for a reasonable fraction of the gene catalogue but also complement it.

Taken together, these findings highlight the value of combining cultivation with metagenomics to study microbial diversity in the pig intestine and the need to both widen the pool of pig gut microbiomes studied and continue efforts in cultivating pig gut bacteria to obtain important taxonomic groups not currently represented by isolates.

### Functional prediction towards archetypal taxa

The high diversity and still substantial unknown fraction of microbes within gut microbiomes^54,55^ highlight the usefulness of developing simplified communities of cultured microbes that can be used to study molecular mechanisms in controlled experimental settings^14,56,57^.

The literature-derived metagenomes aforementioned^18^ were used as a comprehensive pool of data to infer the composition of minimal communities of isolates that best represent faecal pig microbiomes functionally. Binary (presence/absence) profiling of 284 metagenomes at the level of protein families (PFAMs)^58^ indicated a significant clustering according to the country of origin (Fig. 5d). Pigs from China, which were continuously fed low doses of antibiotics^18^, were distant from the two other groups. The landscape of PFAMs present across all PiBAC isolates covered on average 90% of the PFAMs from the input metagenomes, making this collection suitable for strain selection to best mimic the original functional potential of pig microbiomes (Fig. 5e).

For each metagenome, we selected a set of species out of the 110 available in PiBAC (the number of species selected was determined in a sample-specific manner and was 20 on average) based on an iterative scoring system that optimizes the number of matches vs. mismatches between the reference genomes and the given metagenomes (see details in the ‘Methods’ section). The proportion of minimal communities in which each species was selected, both across the entire cohort and per country, was then plotted: a total of 23 species were characterized by a prevalence >50% in the pigs of at least one country (Fig. 5f). They spanned 13 bacterial families across four phyla, Gram-positive and -negative as well as facultative and strictly anaerobes, including 12 novel taxa described in this study that can produce all major SCFAs (formate, acetate, propionate, butyrate) along with lactate and succinate (Supplementary Fig. 3). Moreover, three of them (*Bilifractor porci*, *Prevotella mizrahii*, and *Oliverpabstia intestinalis*) showed bile salt hydrolase (BSH) activity (see next section). Additional functions covered by this consortium include: complex carbohydrate metabolism (*Fibrobacter intestinalis*, *Bacteroides vulgatus*, and *Prevotella copri*); secondary bile acid production (*Clostridium scindens*); trans-aconitate oxidation and anaerobic glutamate fermentation (*Acidaminococcus fermentans*); butyrate production from amino acids (*Intestinimonas butyriciproducens*). The sole species picked in 100% of the pigs across all three countries was *S. pleomorphus*, followed closely by *Prevotella mizrahii* (>98%), which may reflect the importance of these novel taxa in pig microbiomes. In Chinese pigs, *Streptococcus alactolyticus* was selected in all samples vs. 50% in samples from France and 71% from Denmark. This may be explained by common usage of antibiotics in the Chinese pigs studied^59^ and the detection of multiple antibiotic resistance genes such as *lsaE*, *lnuB*, *ant(9)-Ia*, and *ant(6)-Ia* in the isolate’s genome (Supplementary Data 1).

In summary, the proposed species have a high degree of phylogenetic and functional diversity and can now be used as minimal bacterial consortia in experimental and applied studies, e.g. prevent antibiotic use linked to enteric infections in pig farming^23,60^. The composition of minimal consortia will have to be amended according to future progress in isolating and describing additional strains.

### Bile acid-metabolizing features of the pig isolates

**Fig. 6:**
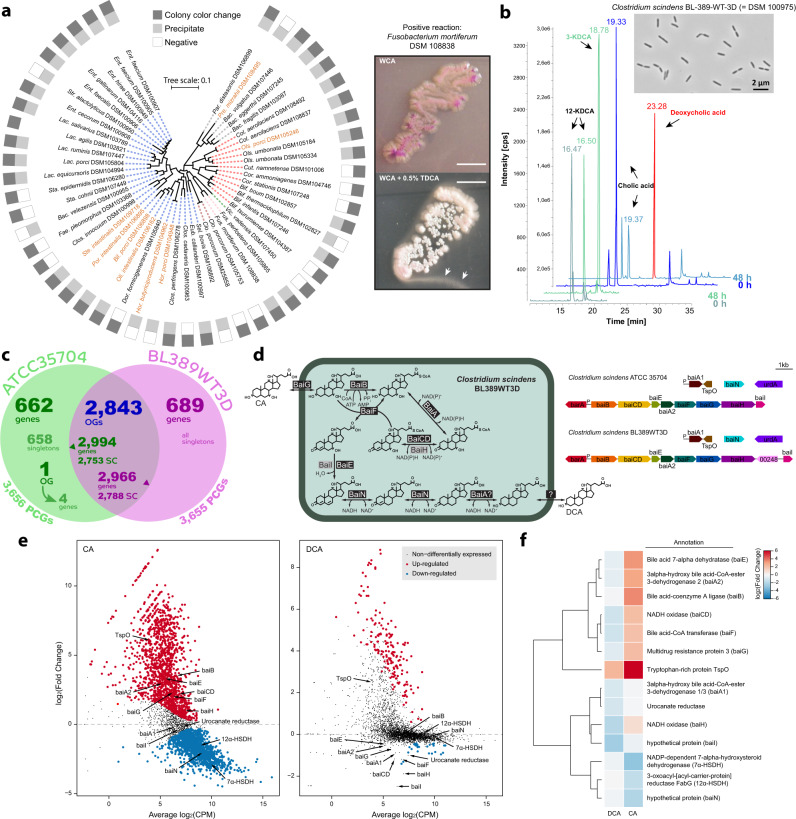
Bile acids metabolism capacities and functional host-specific traits. **a** Left: 16S rRNA gene-based phylogenetic tree showing the diversity of isolates able to deconjugate bile acids. Species (corresponding branches) are coloured according to phyla as in Fig. 1. Right: magnified pictures of a culture of *Fusobacterium mortiferum* grown on plates (WCA, Wilkins–Chalgren-Anaerobe medium) without (top) or with (bottom) addition of 0.5% (w/v) tauro-conjugated deoxycholic acid (TDCA). Two criteria were considered as indicative for positive reaction: (i) whitening of the colonies; (ii) formation of a halo surrounding colonies (solid medium; white arrows in the picture) or precipitates visible at the bottom of the wells and jellification of the medium when grown as liquid cultures (not shown). The scale bars represent 5 mm. **b** Chromatograms showing the conversion of cholic acid to deoxycholic acid by *C. scindens* DSM 100975. KDCA, ketodeoxycholci acid. The two chromatograms on top span the entire run time. The bottom ones are zoom-in sections (XIC:+MRM *m/z* 424.367/371.300 Da) until the indicated time to visualize the appearance of 3-KDCA. **c** Venn diagram of comparative genomic analysis between the human faecal isolate *C. scindens* ATCC 35704 and the pig isolate DSM 100975 (=BL-389-WT-3D). SC single-copy, PCG protein-coding genes, OG orthologous group. **d** Gene organization of the bile acid-inducible (*bai*) genes in the two strains and overview of the bile acid 7-*alpha*-dehydroxylation biochemical pathway. **e** Scatterplots of average log_2_(CPM) vs. log_2_(fold-change) from duplicate 0.1 mM cholic acid (CA) or deoxycholic acid (DCA) induced cultures of *C. scindens* DSM 100975. Genes involved in bile acid metabolism are labelled. **f** Heat map of bile acid-metabolizing genes from CA- and DCA-induced transcriptomes. Genes included in **e** and **f** were differentially expressed (+/−) 0.58 log_2_(FC) with false discovery rate (FDR) < 0.05.

To further characterize the isolates functionally, we studied their ability to convert bile acids, key mediators of microbe–host interactions^61^. In contrast to numerous sequencing studies^62–65^, knowledge of cultured bile acid-metabolizing bacteria is scarce, which hampers applications towards targeted modulation of secondary bile acid metabolism. Nearly half of the collection (*n* = 50 strains across six phyla) showed cleavage of taurine residues from bile acids in vitro (Fig. 6a). This shows that multiple phylogenetically diverse species are capable of deconjugating bile acids, providing cultured isolates to previous metagenomic findings^62,64^. In contrast, only one isolate, strain BL-389-WT-3D (=DSM 100975) of the species *Clostridium scindens* was able to produce the secondary bile acid deoxycholic acid (DCA) from cholic acid (CA) via 7-*alpha*-dehydroxylation (Fig. 6b). Although *Collinsella aerofaciens* is known to dehydrogenate primary bile acids^66^, the two strains of this species within the collection were inactive on CA under the conditions tested. Due to the impact of secondary bile acids on colonization resistance against pathogens^67,68^, metabolic health^69^, and intestinal inflammation and tumorigenesis^70,71^, *C. scindens* DSM 100975 was characterized further.

Among the 3655 protein-coding genes detected in its closed genome, comparative genomics with the human isolate *C. scindens* ATCC 35704^T^ revealed 2966 genes in common (distributed in 2843 orthologous groups) (Fig. 6c)^72^. A substantial fraction of the 689 genes unique to the pig isolate were open reading frames (ORFs) of unknown function or mobile genetic elements (Supplementary Data 3). Indeed, several large gene clusters (CS_BL389WT3D; p_00410-00671, p_00936-00958, p_01430-01477, p_02151-02499, p_02513-02560, p_02778-02828) appeared to encode phage-related genes. A similar observation was made in the 662 genes unique to the human-derived strain ATCC 35704^T^, indicating the acquisition of distinct mobile genetic elements and phages in the distinct host colonic environments. A feature unique to the human strain is the cortisol-inducible *desABCD* operon reported to encode steroid-17,20-desmolase and 20-*alpha*-hydroxysteroid dehydrogenase involved in the conversion of cortisol to 11-oxy-androgens^73,74^. The gene organization of the bile acid inducible (*bai*) operon, responsible for 7-*alpha*-dehydroxylation of primary bile acids, was nearly identical in both strains (*baiBCDEAFGH*; CS_BL389WT3Dp_00241-00249) (Fig. 6d). In DSM 100975 (pig isolate), the *baiI* gene encoding putative bile acid 7-*alpha*-dehydratase is downstream of *baiH* following a single ORF of unknown function (CS_BL389WT3Dp_00248).

The pig-derived strain did not grow in the defined medium recently reported for ATCC 35704^T^ (ref. ^72^), indicating that additional nutrients required for growth remain to be determined. We then performed RNA-Seq analysis of strain DSM 100975 in the absence (PYF control medium) or presence of bile acids (0.1 mM CA or 0.1 mM DCA). A total of 1393 genes (including 709 hypothetical proteins) were significantly upregulated by CA (>0.58 log_2_FC; FDR > 0.05), whilst 1336 (359 hypothetical proteins) were downregulated (Fig. 6e). Significant upregulation of the *baiBCDEAFGH* genes was only induced by CA (2.00 to 3.53 log_2_FC; FDR = 0.016 to 8.1 × 10^−5^) (Fig. 6f). Expression of the *baiN* gene, previously reported to encode a flavin-dependent reductase involved in the reductive arm of the *bai* pathway, was downregulated by both CA and DCA, which is distinct from the constitutive expression in ATCC 35704^T^ (ref. ^75^).

In conclusion, these in vitro assays provide multiple strains able to metabolize bile acids and highlight differences between a pig and a human isolate. This fits with the concept of functional adaptation of intestinal bacteria to a specific host and associated environmental conditions^76,77^, although a higher number of strains will be required for comprehensive analysis. PiBAC can now be used as a toolbox for in vitro and in vivo studies of the impact of bile acids on microbe–microbe and microbe–host interactions.

The pool of yet undescribed bacteria is still very large^17,54,55^. Contemporary loss of bacterial diversity is a real concern^78,79^, stressing the need to archive strains and describe novel taxa, as presented in this study. We focused on pig gut microbiota considering the importance of pigs in biomedical research and agriculture and the host specificity of intestinal microbiomes^18,19,80,81^. The collection of isolates presented here reveals a substantial taxonomic and functional diversity of bacteria that opens avenues for mechanistic studies and biotechnological applications. Important members of the pig gut microbiome such as Archaea, fungi, and strains of yet unknown bacterial phyla are not included and the current resource does not address strain-level diversity. Hence, this work also shows that efforts to isolate and characterize known and yet uncultured microbial taxa from the intestine must be continued.

## Methods

### Pig gut samples

Detailed information about the origin of all strains, including isolation site, donors, and gut region are provided in Supplementary Data 1.

For the 98 strains isolated in Germany, gut samples from 19 pigs were used, including German Landrace, German Landrace crossed to minipigs, p53 and APC^1311/+^ pigs^4,82^ from the Technical University of Munich (animal facility Thalhausen), and one Aachen minipig^83^ hosted at the RWTH University Hospital (Institute of Experimental Animal Science). Fresh faecal samples were collected directly from the rectum of individual pigs using sterile gloves and prepared immediately as described below in the section ‘Bacterial isolation, cultivation, and storage’. Besides faeces, intestinal content was collected using sterile equipment from pigs euthanized for the purpose of other experiments. Animal use was approved by the Federal Government of Bavaria (approval no. ROB55.2-2-2532.Vet_02-18-33, 29).

Additional strains were isolated: (i) at the National Animal Disease Centre of the United State Department of Agriculture (Iowa, USA) from mixed bred pigs obtained from a commercial supplier (*n* = 6 isolates). After euthanasia, samples were collected from the lumen and mucosa of the ileum, caecum, and mid-colon for bacterial cultivation. Piglets were acquired and managed in accordance with the National Animal Disease Centre Animal Care and Use Committee guidelines; (ii) at the University of Guelph (Canada) from farmed ‘wild’ boars, i.e., living in a free-range enclosure and left to forage, breed, and raise their young with minimal human intervention (*n* = 10 isolates). Permission of the farmer was asked prior to sampling. Defecation was watched from a safe distance, and droppings were collected from three separate boars once they had moved away, and within 10 min of voiding. Faecal samples were immediately placed in sterile sample collection bags and onto ice packs, transferred to the laboratory and stored at −80 °C. At the time of culture, samples were carefully thawed, and a ‘core’ region from inside of the dropping was removed for further work, to minimize contamination from the ground; (iii) at the Department of Bacteriology, University of Wisconsin-Madison, USA, as described in detail previously (*n* = 3 isolates)^84^.

### Culture media

The culture medium used for the isolation of each strain is given in Supplementary Data 1. Medium compositions are listed below (all quantities are per litre of distilled water). All media were supplemented with a redox potential indicator (either resazurin, 1 mg, or phenosafranine, 2.5 mg) and with 15 g agar whenever appropriate.

BBE: *Bacteroides* bile esculin agar with amikacin (BD, ref. PA-254480.02).

BHI: Brain Heart Infusion (Oxoid, ref. CM1135), 37.0 g; l-cysteine, 0.5 g; dithiothreitol (DTT), 0.2 g.

Bifido: *Bifidobacterium* Selective Agar (Anaerobe Systems, ref. AS-6423).

Blood agar: Columbia Agar with Sheep Blood PLUS (Oxoid, ref. PB5039A).

BSM: Bifidus Selective Medium (Millipore, ref. 90273), 42.5 g; BSM Supplement (Millipore, ref. 116,83055) 116 mg.

CA: Cellulose Agar: KH_2_PO_4_, 1 g; MgSO_4_, 0.1 g; NaCl, 0.25 g; CaCl_2_, 0.05 g, cellulose, 0.3 g.

Corio2: M9 minimal salts (Merck, ref. M6030), 11.3 g; Tween-80, 1 ml; PdCl_2_, 0.8 g (dissolved first in 1 M HCl). After autoclaving (121 °C, 15 min): bile salts (Fluka ref. 48305) 4 g; mucin type III (Sigma, ref. M1778), 250 mg; arginine, glycine, histidine, and lysine, each 6 g; ampicillin, 50 mg; neomycin 50 mg/l; l-cysteine, 0.5 g; DTT, 0.2 g; sheep blood (Thermo Fischer, ref. R54008), 50 ml.

FAA: Fastidious Anaerobe Agar (Acumedia, ref. LAB090), 46 g; as such or supplemented with either (**I**) nalidixic acid and colistin, 10 mg each; (**II**) defibrinated sheep blood, 5% (v/v); (**III**) filter-sterilized spent culture medium (from a bioreactor inoculated with human faeces)^85^, 3%.

FAT: Beef fat, 10 ml (500 g organic beef was boiled and fat was collected from the surface); Tween-20, 1 ml; tryptone, 2 g. After autoclaving: filter-sterilized pig faecal water, 6 ml; rumen fluid (Bar Diamond Inc., ID, USA), 50 ml; vitamin solution (as in DSMZ medium 141), 1 ml; l-cysteine, 0.5 g; DTT, 0.2 g.

FSA: *Fusobacterium* Selective Agar (Anaerobe Systems, ref. AS-6427).

GAM-mod: Gifu Anaerobic Medium, modified (HyServe, ref. 05433), 41.7 g; l-cysteine, 0.5 g; DTT, 0.2 g.

HM: Hog gastric mucin medium^86^; hog gastric mucin, 10 g; CaCl_2_, 0.45 g; MgSO_4_, 0.45 g; KH_2_PO_4_, 2.25 g; K_2_HPO_4_, 2.25 g; NaCl, 4.5 g; (NH_4_)_2_SO_4_, 4.5 g; cysteine, 0.05 %; haemin, 0.05 g; resazurin, 0.0001 %; Noble agar 1.6 %.

LKV: Laked Brucella Blood Agar with kanamycin and vancomycin (Anaerobe Systems, ref. AS-112).

McConkey: Roth, ref. X922.1.

MDM: Modified Dehority Medium (DSMZ medium 1668) supplemented with cellobiose, 10 g.

Medium 78: Chopped Meat Medium (DSMZ medium 78).

MRS: Lactobacillus Broth according to De Man, Rogosa, and Sharpe (DSMZ medium 11).

MUC: KH_2_PO_4_, 0.4 g; Na_2_HPO_4_, 0.53 g; NH_4_Cl, 0.3 g; NaCl, 0.3 g; MgCl_2_·6H_2_O, 0.1 g; CaCl_2_, 0.11 g; NaHCO_3_, 4 g; Na_2_S·9H_2_O, 0.25 g; alkaline trace element solution, 1 ml; trace element solution (according to DSMZ medium 141), 1 ml; mucin type III (Sigma, ref. M1778), 2.5 g. After autoclaving (121 °C, 15 min): vitamin solution (according to DSMZ medium 141), 1 ml; filter-sterilized human faecal water, 7 ml.

NM: M9 minimal salts (Merck, ref. M6030), 11.3 g; yeast extract, 0.2 g. After autoclaving (121 °C, 15 min): penicillin and streptomycin, each 25 mg; trace element and vitamin solutions (as DSMZ medium 141); ethanol and methanol (each 3 ml).

Nutrient agar: DSMZ medium 1.

Oil-RF-PBS: Phosphate-buffered saline, 500 ml; rumen fluid, 500 ml; Tween-20, 0.5 ml; olive oil, 5 ml. Used for pre-cultivation (5 days) prior to plating on other media.

PG: Postgate Medium^87^: KH_2_PO_4_, 0.5 g; NH_4_Cl, 1 g; Na_2_SO_4_, 2 g; CaCl_2_·6H_2_O, 0.1 g; MgSO_4_·7H_2_O, 1 g; sodium lactate, 3.5 g; yeast extract, 1 g; thioglycolic acid, 0.1 g; FeSO_4_·7H_2_O, 0.5 g; NaHCO_3_, 2 g; PdCl_2_, 2 mg. After autoclaving (121 °C, 15 min): penicillin, 25 mg; trace element and vitamin solutions (as in DSMZ medium 141).

RF-PBS: Phosphate-buffered saline, 500 ml; rumen fluid, 500 ml. RF-PBAS was used for pre-cultivation (5 days) prior to plating onto other media.

SAB: sodium acetate, 1 g; trypticase, 2 g; yeast extract, 2 g; L-cysteine hydrochloride monohydrate, 0.5 g; valeric acid, 5 mM; isovaleric acid, 5 mM; 2-methylbutyric acid, 5 mM; isobutyric acid, 6 mM; 2-methyl valeric acid, 5 mM; NiCl_2_·6H_2_O, 1.5 mg; FeSO_4_·H_2_O, 0.5 mg; MgSO_4_·7H_2_O, 0.8 g; KH_2_PO_4_, 0.5 g; K_2_HPO_4_, 0.5 g; KCl, 0.05 g; CaCl_2_·7H_2_O, 0.05 g; NaCl, 1.5 g; NH_4_Cl, 1 g; MnSO_4_·7H_2_O, 0.6 mg; ZnSO_4_·7H_2_O, 0.1 mg; CuSO_4_·5H_2_O, 0.02 mg; KAl(SO_4_)_2_·12H_2_O, 0.2 mg; H_3_BO_3_, 7 mg; CoSO_4_·7H_2_O, 4 mg; Na_2_MoO_4_·2H_2_O, 0.5 mg; Na_2_SeO_3_·5H_2_O, 3 mg; Na_2_WO_4_·2H_2_O, 4 mg; nitrilotriacetic acid, 0.15 mg; resazurin, 1 mg. The following compounds were aseptically added to the medium after autoclaving: streptomycin, 100 mg; tetracycline, 100 mg; penicillin G, 100 mg; NaHCO_3_, 2 g; Na_2_S, 0.4 g; methanol, 3.3 ml; sodium formate, 11 g; and vitamin solution (DSMZ medium 141), 1 ml. SAB medium was used for pre-cultivation (5 days) prior to plating onto other media.

SM: Tryptic peptone, 5 g; peptone, 5 g; yeast extract, 10 g; beef extract, 5 g; K_2_HPO_4_, 2 g; Tween-80, 1 ml; salt solution (as in DSMZ medium 104), 40 ml. After autoclaving (121 °C, 15 min): hemin (dissolved in 1 M NaOH), 5 mg; vitamin K1 (dissolved in 95% ethanol), 1 µl; glucose, 5 g; l-cysteine, 0.5 g.

WCA: Wilkins–Chalgren Anaerobe broth (Oxoid, ref. CM0643), 33.0 g; l-cysteine, 0.5 g; DTT, 0.2 g. Either used as such or supplemented with mucin, 18.5 g (**WCA-MUC**) or with bromoethanesulfonic acid, 0.221 g, and sodium molybdate, 0.205 g (**WCA1**).

### Bacterial isolation, cultivation, and storage

The rationale behind this isolation project was to establish a collection of bacteria that is of sufficient size to act as a valuable resource for the scientific community and brings also important taxonomic diversity and novel functions to light. We deliberately focused on bacteria, which are dominant members of the ecosystem. This is not a sign of neglecting other microbes and viruses in the pig intestine^88–92^, but rather that the diversity of microbes to be catalogued is tremendous and that the required cultivation efforts are colossal if not tailored. As in recently published studies^93–95^, in order to capture as much diversity as possible, we employed an array of rich and selective culture media (see listing and composition in the previous section) in combination with samples from different gut locations (albeit mostly faeces) and pigs of various origins (most of the pigs were from one animal facility in Germany; others originated from two facilities in the USA or were wild boars from a farm in Canada; see detailed listing in Supplementary Data 1). The respective bacterial isolation procedures are described in the following paragraphs. We deliberately focused on cataloguing diversity at the species level instead of isolating multiple strains of known species, as one primary intention was to fill current phylogenetic gaps and also discover novel functions. Moreover, guaranteeing the public access to all strains is associated with multiple time-consuming quality checks that are not yet compatible with the processing of several hundreds to thousands of isolates. Bacterial diversity in the mammalian gut below the species level is very important^96,97^, but out of scope of the present work.

For all strains isolated in Germany, the procedure followed to collect intestinal samples and keep them under strictly anaerobic conditions as soon as possible is shown in Supplementary Fig. 14. Briefly, samples were immediately diluted approximately 1:10 in anoxic PBS supplemented with peptone (0.2% w/v), l-cysteine, and DTT (each 0.5%). After manual homogenization by vigorous shaking, glass flasks were left to stand for max. 1 min to sediment debris. The slurry was diluted again 1:10 in the same anoxic buffered solution contained in another glass flask using a sterile syringe fitted with a needle to inoculate through the sterile rubber stopper and thereby guarantee anoxic conditions. Samples were transported to the lab on ice prior to further processing, which occurred max. 1.5 h after sample collection.

For direct isolation, tenfold dilution series (10^−2^ to 10^−6^) were prepared in an anaerobic workstation (either Don Whitley Scientific, Bingley, UK, or MBraun GmbH, Garching, Germany) containing an atmosphere of N_2_ (89.3%), CO_2_ (6%), and H_2_ (4.7%). Dilutions (10 µl) were pipetted onto one of the culture media listed above and spread on the agar by tilting plates. Cells were incubated at 37 °C for 1–40 days prior to picking. Pure cultures were obtained by re-streaking single colonies at least three times before transfer into broth medium in Hungate tubes^98^ and observation of colony morphology and cells by light microscopy. Strictly anaerobic strains were subjected to growth under aerobic conditions to check for potential latent contaminations with facultative anaerobes. For aerobic isolation, the same procedure as above was followed using aerobic media and incubation conditions. For enrichment procedures prior to plating, faecal slurries were prepared as above and transferred (100 µl) directly into Hungate tubes containing the desired culture medium and incubated for 7–21 days at 37 °C on a shaker. The obtained mixed cultures were then plated as above.

For strains isolated in Ames, Iowa, USA, a previously published protocol^86^ was followed. Briefly, intestinal mucosal surfaces were washed with sterile PBS and scraped with a sterile microscope slide followed by inoculation into HM medium, as were the corresponding luminal contents. Three enrichments were done in series (10 days each) in broth to enhance the growth of mucolytic bacteria before inoculation on solid media. Bacteria were isolated on solid HM medium after incubation at 39 °C for 5 days.

For strains isolated in Canada, 0.5 g of thawed faecal sample was homogenized in degassed Tryptic Soy Broth in an anaerobic chamber. Tenfold dilution series (10^−2^ to 10^−8^) in saline (0.9% NaCl) were plated (100 µl each) onto different solid media types (FAA-I to -III, Nutrient agar, MRS, BHI) in duplicate and incubated either aerobically or anaerobically at 37 °C. After 72 h and every 24 h up to 7 days, isolated colonies were re-streaked onto FAA plates. Bacterial stocks were frozen and stored at −80 °C in freezing media (12% w/v skim milk, 1% v/v glycerol, 1% v/v DMSO).

For maintaining all strains in culture, the standard medium used was anoxic WCA supplemented with l-cysteine and DTT (0.05% and 0.02% (w/v), respectively). Rumen fluid or 6% CO_2_ in the atmosphere were added if necessary (Supplementary Data 1). Media that can be used to cultivate the strains as well as appropriate incubation times are also indicated online (www.dsmz.de/pibac). For storage, cryo-stocks were prepared by mixing freshly grown cultures 1:1 in a sterile, 40% (v/v) glycerol-containing medium. Cryo-aliquots (400 µl) were dispensed into sterile Eppendorf tubes kept under sterile conditions in a laminar flow cabinet under constant flow of the anaerobic gas mixture before freezing on dry-ice. For long-term storage, all strains were deposited at the Leibniz-Institute DSMZ (Braunschweig, Germany).

### Strain identification

Cultures were first identified using a MALDI-Biotyper following the manufacturer’s instructions (Bruker). The identity of interesting strains was confirmed by amplifying the 16S rRNA gene using primers 27f and 1492r^99^, sequencing using primer 27f after PCR-product cleaning, and searching for most closely related species with a valid name using EzBioCloud^100^. For all strains included in the collection, nearly full-length 16S rRNA gene sequences were generated by re-sequencing the aforementioned PCR products using primers 338r, 785f, and 1492r. Contigs were built in MEGA (v10)^101^ and ambiguous positions were double-checked using electropherograms. All sequences, their length, and accession numbers are listed in Supplementary Data 1. The identity of all strains was confirmed at the Leibniz Institute DSMZ using a MALDI-Biotyper and partial sequencing of 16S rRNA genes. A threshold of 98.7% 16S rRNA gene sequence identity using EzBioCloud^100^ was first considered as an indication for novel taxa, and draft genomes were generated for corresponding strains (see method below). For digital DNA:DNA hybridization (dDDH), the Genome-to-Genome Distance Calculator 2.0 (GGDC), a web service freely available at http://ggdc.dsmz.de, provided a genome-based delineation of species by reporting dDDH estimates as well as their confidence intervals. Additionally, species-level delineation was confirmed using ANI values as calculated by FastANI^102^. Genus-level delineation was inferred via percentage of conserved proteins (POCP) using BLASTP^103^ where only matches with an *E*-value <1e−5, identity >40%, and query coverage of >50% were counted^104^. Genome based-phylogenomic trees were created using PhyloPhlAn (v0.99)^52^ based on 400 marker genes.

### Metabolic characterization

Cellular fatty acids were determined at the Leibniz Institute DSMZ for all novel taxa under their respective optimal growth conditions (www.dsmz.de/pibac). Approximately 100 mg (wet weight) of cell biomass was extracted according to the standard protocol of the Microbial Identification System (MIDI Inc., version 6.1; technical note #101). The composition of cellular fatty acids was identified by comparison with the TSBA40 naming table.

The identity of strain DSM 106279 was confirmed to be *Escherichia coli* via genome comparison and enzymatic profiling using the EnteroPluri test following the manufacturer’s instruction (Liofilchem, Italy).

BSH activity was determined using WCA or BHI medium (depending on strains) supplemented with 0.5% (w/v) taurodeoxycholic acid (TDCA) (Merck, Germany) from filter-sterilized aqueous stock solutions. For plate-based assays (1.5% g/l agar), a single colony was picked and transferred onto solid medium with and without TDCA and incubated for at least 5 days at 37 °C (under anaerobic conditions whenever appropriate) until growth was observed. Isolates were considered positive in case of halos surrounding and/or whitening of colonies due to precipitation after deconjugation (Fig. 6a). For liquid assays (if strains did not grow on agar medium), fresh cultures were mixed 1:1 (total volume 2 ml) with TDCA-containing medium in deep 96-well plates, which were sealed with sterile, semipermeable membranes and incubated as above. Positive strains generated precipitates at the bottom of the wells and jellification of the medium. *Fusobacterium mortiferum* DSM 108838 (= FSA-380-WT-2B) was used as positive control.

To test the ability of *C. scindens* DSM 100975 and two strains of *Collinsella aerofaciens* to metabolize bile acids, duplicate fresh cultures were transferred (1:10) in anoxic WCA broth containing 50 µM CA and incubated at 37 °C for 48 h under anaerobic conditions. Samples were taken at 0 h and 48 h, centrifuged (13,000 × *g*, 10 min) and supernatants were collected and stored at −80 °C until analysed by chromatography coupled with mass spectrometry according to our previously described protocols^66^. *Extibacter muris* DSM 28560^T^ was used as positive control^105^.

For novel taxa, the concentration of SCFAs (acetate, propionate, butyrate, valerate), branched SCFAs (isobutyrate, isovalerate), and intermediate metabolites (lactate, succinate, formate) were determined using high-performance liquid chromatography (HPLC). Bacteria were grown in modified YCFA broth (DSMZ medium 1611) supplemented with 0.02% DTT in Hungate tubes for 48 h or 96 h at 37 °C under anaerobic conditions and with shaking (200 r.p.m.). Triplicate cultures were measured for each strain. Negative controls consisted of medium without bacteria. After incubation, samples were centrifuged (10,000 × *g*, 10 min, RT) and supernatants were collected and stored at −20 °C for a maximum of 7 days prior to measurement. Before HPLC analysis, samples were filtered into 2-ml short thread vials with screw caps (VWR International GmbH, Germany) using non-sterile 0.2-µm cellulose membrane filters (Phenomenex, Germany). Vials were then placed in the refrigerated autosampler of the HPLC system, a Hitachi Chromaster 5450 (VWR International GmbH, Germany) fitted with a Refractive Index detector and a Shodex SUGAR SH1011 column (300 × 8.0 mm) (Showa Denko Europe, Germany). A Shodex SUGAR SH-G (6.0 × 50 mm) was used as guard column. The injection volume was 40 µl. The running temperature was 40 °C. The eluent was 10 mM H_2_SO_4_ with a constant flow of 0.6 ml/min. Concentrations were determined using external standards via comparison of the retention time (all compounds were purchased from Sigma-Aldrich). Peaks were integrated using the Chromaster System Manager software (Version 2.0, Hitachi High-Tech Science Corporation). For each of the tested strains, only SCFA concentrations >0.8 mM (limit of detection (LOD) for succinate, lactate, and acetate) or >0.5 mM (LOD for glucose and all other SCFAs) in at least one of the triplicates were considered for calculation. Results are summarized in Supplementary Fig. 3 and appear below in the protologues whenever appropriate.

### Impact of growth medium osmolarity on *Bullifex porci* DSM 105750^T^

The strain was grown for 7 days (168 h) at 37 °C with constant shaking (220 r.p.m.) under anaerobic conditions in Hungate tubes containing 10 ml of culture. For dilution assays, a gradient of medium dilutions (100, 90, 75, 50, 25, and 10%) was obtained by mixing the basal medium (BHI broth supplemented with 10% rumen fluid) with autoclaved and gassed distilled water in appropriate volume ratios. For increasing osmolarity, the medium was supplemented with the appropriate amount of NaCl (0, 0.25, 0.50, 0.75, 1.00, and 2.50%; w/v) prior to gassing and autoclaving.

Triplicate cultures were tested for each condition. The osmolarity of all media was measured using an OSMO Station OM-6050 (Arkray, Kyoto, Japan). Growth was followed overtime by measuring the OD_600 nm_ directly within the Hungate tubes using a CO8000 Cell Density meter. After 1 week of growth, in order to increase cell density for microscopy, cells were centrifuged (3500 × *g*, 10 min) and re-suspended in a lower volume of the corresponding growth medium. Cell morphology was observed by phase contrast microscopy (see below).

### Phase contrast microscopy

The microscopic images in Fig. 2b1 and in Supplementary Figs. 4 and 9 represent cells in their own growth medium. These images were acquired using a N-Achroplan objective (100×/1,25 Oil Ph3 M27) mounted on an Axio Lab.A1 microscope equipped with an Axiocam 105 camera (Zeiss, Jena, Germany).

### Fluorescence staining and microscopy

*B. porci* DSM 105750^T^ was grown at 37 °C for 4 days in BHI broth supplemented with 10% rumen fluid (Bar Diamond Inc., ID, USA), cysteine (0.05% w/v), and DTT (0.02%) under anaerobic conditions (89.3% N_2_, 6% CO_2_, 4.7% H_2_). Cultures (1 ml) were centrifuged (2 min, 5000 × *g*) and washed once with PBS. Harvested cells were incubated for 10 min in the dark at room temperature with the membrane stain FM4-64 (1 µg/ml) and the DNA stain 4′,6-diamidino-2-phenylindole (DAPI) (2 µg/ml). After final PBS wash, cells were placed onto microscope slides and visualized by phase contrast and fluorescence microscopy using a Zeiss Axio Imager.Z2 microscope (Zeiss, Jena, Germany) equipped with a Plan-Apochromat x 63 phase contrast objective lens, appropriate filter sets, and an ORCA-Flash 4.0 LT digital CMOS camera (Hamamatsu Photonics, Shizuoka, Japan) using the Zeiss Zen 2 (Blue Edition) software. Brightness and contrast level of the images were adjusted using ImageJ (version 1.50g).

### Bacterial cultures for further microscopy analyses

*Bullifex porci* DSM 105750^T^, *Pseudoramibacter porci* DSM 106894^T^, *Stecheria intestinalis* DSM 109718^T^, and *Tissierella pigra* DSM 105185^T^ were analysed via scanning electron microscopy at the University Hospital of RWTH Aachen and via transmission electron microscopy at both the University Hospital of RWTH Aachen and at Wageningen University in order to validate results in independent laboratories using different sample preparations based on classical fixation or high-pressure freezing followed by freeze substitution. The strains were cultured at 37 °C under anaerobic conditions in Hungate tubes with constant shaking (200 r.p.m.) as follows: *B. porci*, BHI broth supplemented with 10% rumen fluid, 148 h; *P. porci*, GAM broth, 36 h; *S. intestinalis*, GAM modified broth supplemented with 5% rumen fluid, 96 h; *T. piger*, BHI broth, 24 h. Cell were pelleted (8000 × *g*, 10 min; 3000 × *g* for *B. porci*) and immediately processed.

### Negative staining

Bacteria in their growth medium were allowed to adsorb on formvar-carbon-coated nickel grids (Maxtaform, 200 mesh, Plano, Wetzlar, Germany) for 10 min prior to washing with distilled water. Samples were then stained by placing a drop of 1% phosphotungstic acid (Agar Scientific, Stansted, UK) in distilled water (pH 7.2) onto the grid for a few seconds. The grid edge was carefully laid onto a filter paper to remove the staining solution from the grid. After air drying, samples were examined at the electron microscopy facility of the University Hospital of RWTH Aachen using a Hitachi HT7800 transmission electron microscope operating at an acceleration voltage of 100 kV.

### Scanning electron microscopy

At the Electron Microscopy Facility of the RWTH University Hospital, bacteria were fixed with 3% (v/v) glutaraldehyde (Agar Scientific, Wetzlar, Germany) in 0.1 M Soerensen’s phosphate buffer, washed in phosphate buffer for 15 min, and dehydrated by incubating consecutively in an ascending ethanol series (30, 50, 70, 90, and 100%) for 10 min each and the last step thrice. The samples were critical point dried in liquid CO_2_ (Polaron, GaLa Instrumente, Bad Schwalbach, Germany) and sputter coated (Sputter Coater EM SCD500, Leica, Wetzlar, Germany) with a 10-nm gold/palladium layer. Samples were analysed using an environmental scanning electron microscope (Hitachi S-4800, Hitachi, Japan) with a 10-kV acceleration voltage in a high vacuum environment.

### Transmission electron microscopy

At the Electron Microscopy Facility of the RWTH University Hospital, bacterial cells were fixed with either 0.2% or 1.5% glutaraldehyde in 0.1 M Soerensen’s phosphate buffer for 30 min and then embedded in 1% low melting agarose (Sigma, Steinheim, Germany). They were frozen in a high-pressure freezer (EM ICE, Leica, Wetzlar, Germany) and subsequently embedded into Epon (Serva, Heidelberg, Germany) via freeze substitution. Samples were then incubated in ethanol at −80 °C for 50 h, brought slowly to room temperature, incubated in fresh ethanol three times for 10 min followed by 30 min in propylene oxide (PO), 2 h in a 1:1 mixture of PO and Epon, 2 h in pure Epon, and finally cured in pure Epon for 2 h at 90 °C. Ultrahin sections (70–100 nm) were cut with an ultramicrotome (Reichert Ultracut S, Leica), picked up on Cu/Rh grids (HR23 Maxtaform, Plano, Wetzlar, Germany) and stained with 0.5 uranyl acetate and 1% lead citrate (both EMS, Munich, Germany) to enhance contrast. Samples were examined using a LEO 906 transmission electron microscope (Carl Zeiss, Oberkochen, Germany) operating at an acceleration voltage of 60 kV.

At the Electron Microscopy Centre of Wageningen University, two different protocols were used:

For protocol 1, cultures of *B. porci* in anoxic WCA medium were pelleted by centrifugation (6000 r.p.m., 5 min) and fixed (3 h, 4 °C) using 2% (v/v) formaldehyde and 2.5% (v/v) glutaraldehyde in 0.1 M cacodylate buffer [Na(CH_3_)_2_ AsO_2_·3H_2_O] (CB). Pellets were washed in CB, fixed (1 h on ice) using 1% osmium tetroxide in CB, and then washed twice in CB. The buffer was removed and replaced with 70% (v/v) ethanol overnight and then embedded in a small volume (100–200 µl) of 1% agarose. Ethanol was increased to 90% and then 100% (v/v) with 30-min intervals at room temperature. An equal volume of a 1:1 mixture of 100% PO and ethanol was added and replaced after 30 min by 100% PO for a further 30 min. Finally, PO was replaced by Epon in PO (1:4 mixture) overnight at 4 °C. The specimens were then placed in Epon in PO (1:1 mixture) for 2 h at room temperature and finally in pure Epon at 4 °C prior to transfer to a casting mould for polymerization at 60 °C. Thin sections were cut from the Epon blocks with a diamond knife using a Reichart Ultracut S apparatus (Leica). The sections were transferred to a 50 mesh copper grid, which was dried and stained by immersion in 1% uranyl acetate in distilled water for 10 min and then Reynolds lead citrate stain for 2 min. Whenever ruthenium red was used for staining, the fixation buffer CB included 0.075% (w/v) ruthenium red and the copper grids were stained only in 1% uranyl acetate in distilled water for 10 min. Cells were imaged using a transmission electron microscope (JEOL 1400 plus).

For protocol 2, *B. porci* was cultured as mentioned above in the section: ‘Bacterial cultures for further microscopy analyses’. Cells were pelleted by centrifugation (4500 × *g*, 10 min) and small aliquots of the pellet were frozen in a Leica EM HPM100 high-pressure freezing system. Freeze substitution was carried out with 0.2% osmium tetroxide and 0.1% uranyl acetate in acetone for 24 h at −90 °C, 24 h at −80 °,C and 24 h at −60 °C using a Leica EM AFS2 equipped with a freeze substitution processor. The temperature was then raised to −20 °C in 12 h and the samples were washed six times with acetone. The temperature was raised further to 4 °C and the samples were infiltrated with acetone:Spurr resin mixtures 2:1, 1:1, and 1:2 each for 6 h. Finally, the samples were infiltrated with pure Spurr for 3 × 6 h at 20 °C and the resin was polymerized for 8 h at 70 °C. Thin sections were cut from the Spurr blocks with a diamond knife using a Leica Ultramicrotome UC7. The sections were transferred to 50 mesh copper grids and stained by UranyLess (10 min) and lead citrate (10 min) both ready-to-use staining solutions from Electron Microscopy Sciences (Hatfield, PA, USA). Cells were imaged using a JEOL 1400 plus transmission electron microscope operating at an acceleration voltage of 120 kV.

### Peptidoglycan analysis

Sacculi samples obtained by boiling bacterial biomass in 5% SDS were used for peptidoglycan isolation^106,107^. Two treatments were tested before muramidase digestion: (i) DNase, RNase, α-amylase, and α-chymotrypsin alone or (ii) with addition of LiCl, EDTA, and HF treatments after protease inactivation. The soluble fraction obtained from muramidase digestion was reduced using 0.5 M sodium borate (pH 9.5) and sodium borohydride (10 mg/ml) and the pH was adjusted to 3.5 with phosphoric acid for liquid chromatography (LC). Solubilized muropeptides were detected and analysed by LC-MS using UPLC coupled with a Xevo G2/XS Q-TOF mass spectrometer (Waters Corp.) operated in positive ionization mode^108^. Data were acquired and processed using the UNIFI software package (Waters Corp.). The molecular structure of muropeptides included in the UNIFI compound library was obtained using ChemSketch (www.adclabs.com).

### Large-scale 16S rRNA gene amplicon analyses

To evaluate the occurrence of isolates, datasets were downloaded from the IMNGS database^47^. All 16S rRNA amplicon samples that contained >5000 sequences and were labelled as ‘pig gut’ (*n* = 1346), ‘mouse gut’ (*n* = 11,442), or ‘human gut’ (*n* = 11,468) were used. Random subsampling of both the mouse and human samples to a number of 1346 samples was conducted to ensure comparative results pertaining to host specificity. The 16S rRNA genes of all isolates were compared to IMNGS-derived OTU sequences using blastn (*E*-value <1e−25, 97% identity, 80% query coverage). Annotation of OTUs to the Living Tree Project database^48^ was conducted using the same parameters. Collection members were considered as being enriched within the pig vs. mouse and human gut if detected in at least 20% of the pig samples and at a higher median relative abundance as determined by Wilcoxon Rank Sum test after Benjamini–Hochberg correction (adj. *p*-value <0.05). To determine common and unique IMNGS-derived OTUs across the host species, the SILVA database (v132)^109^ was reduced to single representatives per species via clustering at 97.0% using Usearch (v8.1)^110^. The subsampled host-specific datasets (*n* = 1346 samples each) were annotated against the reduced SILVA database using Usearch at 97% identity to provide a common reference point for species occurrence.

### Targeted 16S rRNA gene amplicon studies

Besides the large-scale analysis described in the previous paragraph, in order to assess the coverage of sequence-based diversity by all isolates in relation to parameters such as diet, age, and gut locations, we processed three additional datasets: (i) published data^111^ investigating the impact of dietary protein content on the faecal microbiota of castrated male Duroc pigs at the age of 165 days; (ii) stool samples from ten German landrace pigs from the animal facility of Thalhausen (TU Munich, Germany) at the age of 8, 24, and 52 weeks; (iii) samples from six wildtype pigs from the MIDY biobank^5,112^, including five locations within the gastro-intestinal tract. For studies 2 and 3, stool samples were processed and sequenced as previously reported in detail^113^ and briefly described in the next section. All data were analysed using IMNGS^47^ and Rhea^114^. Cultured fractions were determined by searching for matches between the amplicon sequences of all study-specific OTUs (those occurring at a relative abundance of ≥0.25% in at least one sample within the respective dataset) and 16S rRNA gene sequences of the isolates using blastn (*E*-value <1e−25, 80% query coverage) at two different sequence identity thresholds: 97% (as proxy for species level) and 95% (genus level).

### High-throughput 16S rRNA gene amplicon sequencing

Metagenomic DNA was isolated using a modified version of the protocol by Godon^115^. Aliquots of stool mixed in DNA stabilization solution (Invitek) (600 µl) were supplemented with 250 µl of Guanidinethiocyanat (4 M) and 500 µl of N-laurolylsarcosine (5%), mixed and incubated for 1 h at 70 °C under moderate shaking (700 r.p.m.). Cell were mechanically lysed by bead-beating (three cycles: 40 s; 6.6 m/s) with 0.1-mm glass beads using a Fast-Prep-24 fitted with a cooling adapter. The cell lysate was then vortexed with 15 mg of Poly(vinylpolypyrrolidone) and centrifuged (12,000 × *g*, 3 min, 4 °C). The clear intermediary phase was transferred into a new tube and centrifuged again. After addition of 5 µl of RNase (10 mg/ml), samples were incubated (37 °C, 20 min, 700 r.p.m.) followed by DNA purification using the NucleoSpin gDNA kit (Machery-Nagel, No. 740230.250) according to the manufacturer’s instructions. The V3–V4 regions were amplified (25 cycles) from 24 ng of template DNA in a two-step process^116^ using primer 341F and 785R^117^ and a combinatorial dual indexing strategy. PCR products were purified using magnetic beads (Beckman Coulter) and pooled in an equimolar amount of 2 nM. The multiplexed samples were sequenced on an Illumina MiSeq in paired-end mode (2 × 275 bp) using the Rapid v2 chemistry.

### Genome sequencing

Genomes were generated for all strains within the collection, including 115 draft genomes and two closed genomes for *Sodaliphilus pleomorphus* DSM 108610^T^ to refine taxonomic assignment and for *C. scindens* DSM 100975 to optimize RNAseq and comparative genomic analyses.

For draft genomes, DNA libraries were prepared using either the TruSeq® DNA PCR-Free Sample Preparation Kit (Illumina) following a protocol optimized (DNA shearing and fragment size selection) to improve assembly quality when sufficient amounts of DNA were available^118^ or using the NEBNextra Ultra II DNA Library Prep Kit (New England Biolabs) otherwise. Libraries were sequenced using the Illumina MiSeq system according to the manufacturer´s instructions.

Whole-genome sequencing of *S. pleomorphus* was carried out on a PacBio RSII (Pacific Biosciences, USA) using P6 chemistry. The genome was assembled using the ‘RS_HGAP_Assembly.3’ protocol included in SMRT Portal version 2.3.0, utilizing 68,258 postfiltered reads with an average length of 11,462 bp. The obtained chromosomal contig was trimmed, circularized, and adjusted to *dnaA* (gene for chromosomal initiation protein DnaA). For short-read correction, sequencing was also carried out on a NextSeq (Illumina, USA) in a 150-bp paired-end run. Quality improvement was performed with the Burrows-Wheeler Aligner (BWA) mapping the Illumina reads onto the obtained chromosome^119^. The final genome sequence was annotated using Prokka^120^.

To generate a closed genome for *C. scindens* DSM 100975, we used a combination of Oxford nanopore (74,727 reads, max. 143,000 bp, min. 1030 bp) and Illumina (411,178 reads, each 250 nt) sequencing. DNA was converted into Nanopore libraries using the NBD104 barcoding kit and 1D library kit SQK-LSK109. Libraries were sequenced on a SpotON R9.4.1 FLO-MIN106 flowcell for 48 h using a GridION x 5 sequencer. Basecalling was performed with Guppy 3.0.3 (Oxford Nanopore Technologies, UK). Demultiplexing and adaptor trimming from the fastq files was performed with Porechops 0.2.3 (https://github.com/rrwick/Porechop). Shotgun genomic libraries were prepared with the Hyper Library construction kit from Kapa Biosystems (Roche, Switzerland). The libraries were quantitated by qPCR and sequenced on one Illumina MiSeq flowcell for 251 cycles from each end of the fragments (250 nt length) using a MiSeq 500-cycle sequencing kit Nano. Fastq files were generated and demultiplexed with the bcl2fastq v2.20 conversion software (Illumina, USA).

Genome sequences were submitted to the NCBI and are available within BioProject PRJNA561470. Individual genome accession numbers are given in Supplementary Data 1.

### Genome processing

Illumina reads were assembled using Spades (v3.6.1)^121^ with activated BayesHammer tool for error correction and MismatchCorrector module for post-assembly mismatch and indel corrections. Assemblies were evaluated using Quast (v3.1)^122^ and CheckM (v1.0.12)^123^. Whenever appropriate, genomes were circularized using Circlator-1.4.1 (https://github.com/sangerpathogens/circlator). Genes were predicted and annotated using Prokka, accepting only closed end CDS (default setting)^120^. Pathway analysis, including the identification of cell wall and divisome proteins, was conducted by converting Prokka output into KEGG orthologue IDs using Prokka2KEGG (https://github.com/SilentGene/Bio-py/tree/master/prokka2kegg) and the KEGG Mapper tool^124^. In addition, genomes lacking a KO for FtsK were instead searched for the SpoIIIE gene and, if found, assigned to the FtsK/SpoIIIE function, as SpoIIIE is a highly related protein commonly grouped with FtsK due to their shared functionality. The presence or absence of all functions included in Fig. 2c were reconfirmed via targeted annotation (DIAMOND; >80% query coverage, >80% subject coverage, >30% identity) against databases formed from SWISSPROT, manually curated to include only the proteins named. Antibiotic resistance genes were annotated using DIAMOND with bit-scores >300 and 90.0% identity against the Comprehensive Antibiotic Resistance Database (CARD) homologue models, release October 2017 (ref. ^125^). EggNOG annotations were obtained using eggNOG-Mapper (v2)^126^. Proteins within the pig metagenomic gene catalogue were assigned to isolate- or MAG-derived proteomes using BLASTP (bitscore >100). Comparative genomics of the *C. scindens* strains was performed using OrthoFinder^127^ to infer orthologous groups and in-house scripts for data processing. Functional category analysis was done with the eggNOG-mapper function using bacteria as taxonomic scope and DIAMOND as the search program^128^. Graphing and statistical analysis (Fisher’s exact test with Bonferroni correction) were performed in R.

### In silico prediction of FucT-encoding genes

All proteins from the 38 novel taxa identified and extracted via Prokka (see genome processing above) were annotated in a targeted manner using DIAMOND v0.9.13.114 (ref. ^129^) with a query and subject coverage of 40% along with 40% sequence identity against the GT family 10 subset of the CAZy database (www.cazy.org). Variants of known FucT motifs (e.g. FEN, FxxxFEN) were then searched and matched proteins were entered into the online I-TASSER server^130^ for structural modelling and homology analysis.

### Biochemical characterization of the FucT from *Clostridium porci*

*Escherichia coli* BL21 (DE3) was transformed with a pET21a vector containing the target gene and cultured in TB medium at 37 °C. At OD_600_ of 0.5–0.8, expression was induced with 1 mM IPTG, after which the temperature was reduced to 25 °C and cells were incubated for another 24 h. Cells were harvested by centrifugation and stored at −20 °C until further processing. For purification, 4 g of cells were suspended in Tris-HCl lysis buffer (50 mM, pH 7.5, 500 mM NaCl, 20 mM imidazole), sonicated (6.5 min, 15 s pulse, 60 s pause) and centrifuged. Crude extracts were filtered and loaded onto a HisTrap column (5 ml, GE Healthcare). The column was washed with lysis buffer, and the enzyme was eluted with elution buffer (lysis buffer with 500 mM imidazole). The enzyme was dialyzed overnight in Tris-HCl buffer (50 mM, pH 7.5) and stored at 4 °C. Purification was assessed by SDS-PAGE (12%) and Western Blot.

For GT assays, *N*-Acetyllactosamine (LacNAc) carrying a tBoc linker was produced as described recently^131^. The enzyme (50 µg/ml) was incubated at 37 °C for 24 h in 50 mM MOPS (pH 6), 25 mM KCl, 5 mM MgCl_2_, 6.5 mM GDP-Fucose, 5 mM tetrasaccharide, and 0.02 U/mL FastAP. Negative controls included reactions without GDP-Fucose and with an enzyme from a rumen *Lachnospiraceae* isolate (protein accession SFH53651.1) cloned and expressed as described above for the target protein^44^. Reactions were stopped by heat treatment (5 min, 95 °C) and products were analysed using a Dionex RP-HPLC system (ThermoFisher Scientific, Darmstadt, Germany) at a wavelength of 254 nm, using an analytic MultoKrom 100-5 C18 column (250 mm × 4 mm; CS Chromatographie, Langerwehe, Germany) and a flow rate of 1 ml/min (isocratic eluent of 15% acetonitrile in water). Product peaks were collected and further analysed by ESI-MS (Finnigan Surveyor MSQ Plus, ThermoFisher Scientific, Darmstadt, Germany), using a Multospher 120 RP 18 HP-3µ columns (60 mm × 2 mm; CS Chromatographie, Langerwehe, Germany) and a flow rate of 0.2 ml/min with 50% acetonitrile (needle voltage = 4 kV, temperature = 400 °C, cone voltage = 100 V, negative mode). The product from the GT assay was treated (37 °C, 24 h) with a α3-fucosidase (NEB, Frankfurt, Germany) according to the manufacturer’s instructions to determine the position and glycosidic bond type of fucose. The reaction was analysed via HPLC as described above.

### Genome mining for BGCs

Putative specialized metabolite BGCs were discovered by mining the draft genomes of novel taxa within the collection using antiSMASH 5.0 (ref. ^132^). The function of individual genes within BGCs was further validated by manual curation using BLAST^103^ against the NCBI nr database to identify core genes for biosynthesis. The sequence alignment of the sactipeptide precursor peptide was performed using Clustal Omega^133^ and visualized by Weblogo^134^. Clustal Omega was also use for phylogenetic analyses based on 16S rRNA gene, SCIFF, and rSAM sequences.

### Metagenome-assembled genomes

Genomes were reconstructed using shotgun metagenomic reads from the literature previously used for establishing the first reference gene catalogue of the pig microbiome^18^. All codes are publicly available at https://github.com/strowig-lab/PIBAC (ref. ^50^), referenced under 10.5281/zenodo.4075065. Briefly, the bioinformatic pipeline removed host-derived reads with BBMap using the corresponding Ensembl masked genome from pig and phiX. Megahit^135^ with specific parameters (-kmin 5 -k 27,37,47,57,67,77,87,97) was then applied using a SGI-UV2000 cluster with 256 cores and 2 TB shared memory for an all-in-one assembly of all metagenomic libraries. Contigs >1000 bp were used as reference to map all libraries using BWA^119^ with default settings. Binning was done with MetaBAT (version 0.32) using -verysensitive -pB 20 -B 100 -minclustersize 200000. Clusters were evaluated with CheckM^50,123^. Minimum quality thresholds were >50% completeness and <10% contamination, whilst >90% completeness and <5% contamination were indicators for high-quality MAGs. Their identity was tested against GTDB (release 04-RS89)^53^ and the 38 new taxa from the present study using ANI values <95% as species-level delineation. dRep^136^ was used for genome dereplication (95% Mash ANI). For depicting evolutionary relationships between taxa, analyses were conducted in MEGA7^137^ using the Neighbour-Joining method^138^ and bacteria marker gene alignment from GTDB-Tk. The evolutionary distances were computed using the Poisson correction method^139^ and expressed in the units of the number of amino acid substitutions per site. The analysis involved 609 amino acid sequences. All ambiguous positions were removed for each sequence pair. There were a total of 5040 positions in the final dataset. The occurrence (prevalence and relative abundance) of the 38 new taxa in PiBAC was estimated using bbmap^140^ by transforming mapped reads from the queried genomes to TPM (transcripts per million) values with adjustment to the fraction of unmapped reads for each library. Details and codes are provided here: https://github.com/strowig-lab/PIBAC.

### Prediction of simplified community composition

The same pig gut metagenomes as in the previous section^18^ were expressed into binary vectors (presence/absence) of PFAMs^58^. Iterative search against a local database of the isolates’ genomes returned those isolates that best matched each of the original profiles, considering matches and mismatches to the given metagenome. The number of species picked within minimal communities was determined in a sample-specific manner based on knee-points of cumulative metagenomic PFAM coverage curves using the ‘inflection’ package in R. The prevalence of picked species across the entire pig cohort and their median picking rank were then reported.

### Transcriptomics

*C. scindens* DSM 100975 was cultivated in PYG medium, induced with 0.1 mM CA or DCA and mRNAs were isolated, enriched, sequenced, and analysed as in our previously published studies^72,141^. Raw data were submitted to the NCBI and are available under the BioSample accession numbers SAMN13016626-31.

### Taxonomic descriptions

Thresholds of 98.7%, 94.5%, and 86.5% 16S rRNA gene sequence identities were considered as indications for novel species, genera, and families, respectively^142^. dDDH values <70% and ANI values <95% were considered as an indication for separate species. As within-species differences in the genome-based G+C content of DNA are almost exclusively <1%, larger differences also supported the status of distinct species^143^. POCP values <50% were considered as an indication for separate genera^104^. The classification of isolates based on genomes was also assessed using GTDB-Tk^53^ and by constructing phylogenomic trees to either confirm or deny novelty and genus delineation.

Description of *Anaerococcus porci sp. nov*.: *Anaerococcus porci* (por’ci. L. gen. n. *porci* of a pig). The closest phylogenetic neighbour within family *Peptoniphilaceae* (phylum Firmicutes) is *Anaerococcus vaginalis*, which shares a 16S rRNA gene sequence identity of 96.7%. The dDDH and ANI values of 22.2% and 81%, respectively, between the two genomes confirm the separate species status. Values between the isolate and *Anaerococcus prevotii*, the type species of the genus, are 22.5% and <80.0%. Cells grow as diplococci, are strictly anaerobic and stain Gram-positive. The genome encodes transporter systems for phosphate, alkanesulfonate, proline, monosaccharides, biotin, zinc, and d-methionine. Starch, trehalose, *N*-acetylmuramate, cellobiose, maltose, arbutin, salicin, and sucrose were identified as potential substrates. However, only starch, maltose, and trehalose enter glycolysis whilst the others undergo initial conversion and no subsequent reactions. Myo-inositiol can be converted into acetyl-CoA and enter the TCA cycle via a multistep pathway including conversion to scyllo-inosose (EC: 1.1.1.18) and malonic semialdehyde (EC: 4.2.1.44, 3.7.1.22, 5.3.1.30, 2.7.1.92), which is finally converted into acetyl-CoA (EC: 1.2.1.18). After 48 h at 37 °C in modified YCFA medium under anaerobic conditions, the species consumed glucose (10.2 ± 0.1 mM) and acetate (9.6 ± 0.9 mM) and produced 16.8 ± 0.8 mM butyrate, 9.3 ± 1.5 mM formate, 5.5 ± 0.7 mM lactate, and 2.1 ± 0.1 mM propionate. The predominant cellular fatty acids are C_16:0_ (20%), iso-C_17:1_/anteiso-C_17:1_ (20%), C_18:1 ω9c_ (14%), and C_14:0_ (12%). Other fatty acids are C_16:1 ω7c_/iso-C_15:0 2-OH_ (4.4%), C_12:0_ (3.3%), and C_18:1 ω7c_ (3.3%). The type strain is WCA-380-WT-2B^T^ (=DSM 101005^T^). It was isolated from faeces of a German Landrace pig in Freising, Germany. Its G+C content of genomic DNA is 29.9 mol%.

Description of *Anaerovibrio slackiae sp. nov*.: *Anaerovibrio slackiae* (slack’i.ae. N.L. gen. n. *slackiae* named in honour of Prof. Emma Slack from the ETH in Zurich, Switzerland, for her scientific contribution to the field of immunology and oral vaccines in pigs). According to 16S rRNA gene-based phylogeny, the species is placed into the family *Selenomonadaceae* (phylum Firmicutes). The closest phylogenetic neighbour is *Anaerovibrio lipolyticus*, which shares 94.7% sequence identity (1504 bp) and a POCP value of 54.6%, suggesting that the two species belong to the same genus, which was confirmed using GTDB-Tk. The ANI and dDDH values of <80% and 22.8%, respectively, together with a difference in the G+C content of genomic DNA of 5.3 mol% between the two genomes clearly confirm the status of a new taxon and the need to create a novel species to accommodate the isolate. It grows as short, motile rods that are slightly pear-shaped and curved. Occurs as single cells or in pairs. Passage intervals longer than 2–3 days result in strong reduction or cessation of growth in WCA and BHI broth. Within the genome, five transporters were identified along with ABC transporters for phosphate, alkanesulfonate, teichoic acid, d-methionine, cobalt, and biotin. Arbutin, salicin, d-glucose, sucrose, starch, cellobiose, cellulose, trehalose, and maltose were all indicated to be carbohydrate sources. The detection of genes encoding for FliA/C/D/F/G/M/N/P/S, FlhA/B, FlgB/C/D/E/G/K/L, and MotB indicate the presence of flagella. The RpfC/G two-component system along with the biofilm dispersal gene *manA* suggest that this species is able to form biofilms. This is further supported by the presence of the glycogen biosynthesis genes, *glgA/C/P*, along with its regulator, *csrA*. After 48 h at 37 °C in YCFA medium under anaerobic conditions, the species consumed glucose (11.3 ± 1.0 mM) and produced 19.3 ± 1.4 mM propionate, 11.0 ± 1.4 mM acetate, and smaller amounts of lactate (1.1 ± 0.1 mM). Major cellular fatty acids are C_15:0_ (20%), C_13:0 3OH_/C_15:1 i I/H_ (15%), and C_17:1 ω8c_ (13%). Lower amounts of the following fatty acids were detected: C_17:0_ (7.5%), C_15:1 ω8c_ (7.4%), C_16:1 ω9c_ (6.7%), C_11:0_ (5.4%), C_13:0_ (4.9%), C_14:0 3OH_/iso-C_16:1_ (4.7%), C_16:0_ (4.2%), and iso-C_18:1_ (3.8%). The type strain is WCA-693-APC-5D-A^T^ (=DSM 108025^T^). It was isolated from faeces of an APC^1311/+^ pig in Freising, Germany. Its G+C content of genomic DNA is 48.7 mol%.

Description of *Baileyella gen. nov*.: *Baileyella* (Bai.ley.el’la. N.L. dim. fem. n. *Baileyella* named in honour of Prof. Mick Bailey from the University of Bristol, UK, for his contribution to the field of immunology using pig models). Based on 16S rRNA gene sequence analysis, the genus is phylogenetically placed into the yet undescribed family ‘*Mogibacteriaceae*’ (phylum Firmicutes). Phylogenomic analysis via GTDB-Tk classifies the isolate within the genus *Mogibacterium* and the POCP value to *Mogibacterium pumilum*, the type species of the genus, is 51.2%, which is nearing the genus-level threshold of 50%. However, we consider it not sound to propose a novel species within this genus to accommodate the isolate because its closest 16S rRNA gene-based phylogenetic neighbour *M. pumilum* shares only 92.3% 16S rRNA gene sequence identity (1430 bp), which is markedly below established genera delineation thresholds. The difference in the G+C content of genomic DNA between the two species is 5.4 mol%. The corresponding ANI and dDDH values are <80% and 20.1%, respectively. These findings clearly demonstrate the status of a novel taxon. Taken together, we propose the creation of a novel genus, for which the name *Baileyella* is proposed. Members of the genus are strictly anaerobic. The type species is *Baileyella intestinalis*.

Description of *Baileyella intestinalis sp. nov*.: *Baileyella intestinalis* (in.tes.ti.na’lis. N.L. fem. adj. *intestinalis* pertaining to the gut). The species has all the features of the genus. It forms short, straight rods (ca. 1 µm) that grow as single cells. Within the genome, nine transporters were identified along with ABC transporter systems for phosphate, osmoprotectants, and zinc. Starch was the only predicted carbohydrate source potentially utilized. Folate biosynthesis was identified from serine via conversion into 5,10-methylene-THMPT (EC: 2.1.2.1, 1.5.1.3). Serine production from hydroxy-pyruvate was identified via 2-phospho-d-glycerate and phosphoserine (EC: 1.1.1.29, 2.7.1.165, 5.4.2.12, 1.1.1.95, 2.6.1.52, 3.1.3.3). Serine can also be directly converted into l-tryptophane (EC: 4.2.1.20). The species is positive for lipase production using the Rhodamine B agar assay. The predominant cellular fatty acids are iso-C_19:1_ (19%), C_18:0_ (18%), C_18:1 ω9c_ (14%), C_18:1 ω6c_ (11%), and C_16:0_ (11%). Other fatty acids measured at lower amounts included iso/anteiso-C_17:1_ (7.7%), C_17:1 ω9c_ (7.3%), C_12:0_ (3.3%), and C_14:1 ω5c_ (2.3%). The type strain is RF-744-FAT-WT-3^T^ (=DSM 106896^T^). It was isolated from faeces of an Aachen minipig^83^ in Germany. Its G+C content of genomic DNA is 29.9 mol%.

Description of *Berryella gen. nov*.: *Berryella* (Ber.ry.el’la. N.L. dim. fem. n. *Berryella* named in honour of Prof. David Berry from the University of Vienna, Austria, for his contribution to the field of microbial ecology). The 16S rRNA gene sequence of this isolate (1389 bp) shows highest identity with species of the genus *Adlercreutzia*, with values ranging from 92.6% for *Adlercreutzia caecimuris* to 94.1% for *Adlercreutzia equolifaciens*, the type species of the genus. The isolate is thus placed into the family *Eggerthellaceae* within the phylum Actinobacteria. POCP values between its genome and the genome of any of the *Adlercreutzia* spp. range from 48.5% to 51.9% and thus oscillate around the proposed genus-delineation threshold of 50%. GTDB-Tk analysis confirmed the status of novel genus within the family *Eggerthellaceae*. Of note, one strain designated Marseille-P7992 shares 99.8% 16S rRNA gene sequence identity as well as ANI, dDDH, and POCP values of 97.0%, 77.0%, and 84.6% with our isolate, respectively. This indicates that the two isolates belong to the same species. However, strain Marseille-P7992 has no official standing in the nomenclature. The type species of our proposed genus is *Berryella intestinalis*.

Description of *Berryella intestinalis sp. nov*.: *Berryella intestinalis* (in.tes.ti.na’lis. N.L. fem. adj. *intestinalis* pertaining to the gut). As many members of the family *Eggerthellaceae*, the species forms small rods that occur as single cells or in pairs, stain Gram-positive, and show low turbidity after growth in broth. Within the genome, 24 transporters along with ABC transporter systems for phosphate, d-methionine, zinc, biotin, aspartate, glutamate, and glutamine were detected. No carbohydrate sources were identified to be utilized by this species. Formate production from CO_2_ (EC: 1.17.1.10) and subsequent conversion into pyruvate via serine may be one method by which this species produces energy (EC: 6.3.4.3, 1.5.1.5/3.5.4.9, 2.1.2.1, 4.3.1.17). Ammonia was able to be converted into l-glutamine (EC: 6.3.1.2), which in-turn can be utilized to form l-glutamate (EC: 1.4.1.13). Glutamate can be used to produce ornithine (EC: 2.3.1.1, 2.7.2.8, 1.2.1.38, 2.6.1.11, 2.3.1.35), which may be converted into citruline (EC: 2.1.3.3) and then to arginine (EC: 3.5.3.6). The co-factor siroheme was identified to be produced from glutamate (EC: 6.1.1.17, 1.2.1.70, 5.4.3.8, 4.2.1.24, 2.5.1.61, 2.1.1.107, 1.3.1.76, 4.99.1.4). The main cellular fatty acids are C_18:1 ω9c_ (30%), C_16:0_ (19%), and C_18:0_ (13%). Fatty acids with lower proportions include C_14:0_ (9.1%), C_13:0 3OH_/C_15:1 i I/H_ (6.0%), and iso/anteiso-C_17:1_ (3.8%). The type strain is 68-1-3^T^ (=DSM 104960^T^). It was isolated from the ileal content of a pig in Ames, Iowa, USA. Its G+C content of genomic DNA is 63.6 mol%.

Description of *Bilifractor gen. nov*.: *Bilifractor* (Bi.li.frac’tor. L. fem. n. *bilis* bile; L. masc. n. *fractor* breaker; N.L. masc. n. *Bilifractor* breaker of bile, pertaining to the strong BSH activity of the type strain of the type species on agar plates). Based on 16S rRNA gene-based phylogeny, the genus falls into the family *Lachnospiraceae* (phylum Firmicutes). The closest phylogenetic neighbours are *Syntrophococcus sucromutans*, *Eubacterium cellulosolvens*, and *Blautia coccoides*, which share 94.3%, 92.4%, and 91.7% sequence identity, respectively (1501 bp). The POCP values between the genome of our isolate and that of *E. cellulosolvens* and *B. coccoides* are 21.6% and 12.8%, respectively, suggesting a separate genus status. The corresponding dDDH values of 27.4% and 23.3% confirm that the isolate represents a different taxon. As no genome is yet available for *S. sucromutans*, corresponding analysis could not be performed. The type species is *Bilifractor porci*.

Description of *Bilifractor porci sp. nov*.: *Bilifractor porci* (por’ci. L. gen. n. *porci* of a pig). The species grows under anaerobic conditions as diplococci that can form long, irregular chains. They stain Gram-negative. A single transporter was identified within the genome along with ABC transporter systems for phosphate, cystine, teichoic acid, biotin, and the RbsA/B/C/D transport system for ribose, autoinducer 2 (AI-2) and d-xylose. AI-2-based quorum signalling is likely to be utilized by this species due to the presence of LuxS and LsrA/B/D. Starch and trehalose were identified to be potential carbohydrate sources. Indole biosynthesis from shikimate may occur via conversion of shikimate to chorismate (EC: 2.7.1.71, 2.5.1.19, 4.2.3.5) and choristmate to indole (EC: 4.1.3.27, 2.4.2.18, 5.3.1.24, 4.1.1.48, 4.2.1.20). Biofilm formation may be favoured via the production of glycogen, as suggested by the presence of the GlgA/C/P genes. The species is considered to deconjugate primary bile acids as determined in vitro by cleavage of taurine residues on WCA agar medium containing TDCA. It is also positive for lipase production using the Rhodamine B agar assay. After 48 h at 37 °C in modified YCFA medium under anaerobic conditions, the species did not use glucose and produced only small amounts of lactate (0.4 ± 0.1 mM). The most dominant cellular fatty acid is iso/anteiso-C_17:1_ (44%). Other fatty acids include C_16:0_ (19.1%), C_16:0 N alcohol_ (5.8%), C_18:0_ (4.2%), and C_13:0 3OH_/C_15:1 I I/H_ (3.3%). The type strain is Oil+RF-744-WCA-WT-13^T^ (=DSM 106898^T^). It was isolated from faeces of an Aachen minipig^83^ in Germany. Its G+C content of genomic DNA is 50.4 mol%.

Description of *Bullifex gen. nov*.: *Bullifex* (Bul’li.fex. L. fem. n. *bulla* bubble; L. suff. -*fex* (from L. v. *facere* to make); N.L. masc. n. *Bullifex* a producer of bubbles, pertaining to the very peculiar cell morphology of the type strain of the type species). This new taxon belongs to the family *Spirochaetaceae* (phylum Spirochaetes). Whilst members of the phylum Spirochaetes are usually helically shaped, this isolate is coccoidal to spherical under the growth conditions tested. It shares 88.4% 16S rRNA gene sequence identity (1499 bp) with *Sphaerochaeta pleomorpha* and 87.7% with *Sphaerochaeta globosa*, the type species of the genus *Sphaerochaeta*. The corresponding ANI/dDDH values of <80%/17.8% and <80%/19.4% between the genome of our isolate and that of the two species aforementioned confirm the discovery of a new taxon. The POCP values of 38.5% and 38.7% justify the creation of a new genus to accommodate this isolate. This is supported by the GTDB-Tk classification within family ‘*Sphaerochaetaceae*’ without further information at the genus level. The type species is *Bullifex porci*.

Description of *Bullifex porci sp. nov*.: *Bullifex porci* (por’ci. L. gen. n. *porci* of a pig). Cells are coccoidal to spherical with a size that varies between 1 and 10 µm. After several days of growth, they tend to form inflated round cells with an opaque, half-moon-shaped compartment on one side of the cell that stains positive with DAPI. The isolate is characterized by slow growth that can be improved by the addition of rumen fluid to the culture medium. Growth and cell morphology are not affected by β-lactam antibiotics (penicillin and ampiciliin; both tested up to 100 U/ml). Within the genome, three transporters were identified along with ABC transporter systems for phosphate, cystine, d-methionine, arabino-oligosaccharide, methyl-galactoside, and biotin. Sucrose and starch were the only carbohydrates sources predicted to be utilized. Glutamate biosynthesis may occur from ammonia (EC: 6.3.1.2, 1.4.7.1/1.4.1.13), which itself is produced from nitrite (EC: 1.7.1.15). Glutamate can then be used for the biosynthesis of ornithine (EC: 2.3.1.1, 2.7.2.8, 1.2.1.38, 2.6.1.11, 2.3.1.35). l-serine can be converted into *O*-acetyl-l-serine (EC: 2.3.1.30) and combined with sulfide to form l-cysteine (EC: 2.5.1.47). The absence of the flagella genes *flgK* and *flgC* within the genome corroborates the non-motile behaviour of the species as observed in vitro. The major cellular fatty acids (11–14%) are C_18:0_, C_12:0_, C_16:0_, and C_18:1 ω9c_. Other fatty acids include iso/anteiso-C_17:1_ (8.8%), iso-C_19:1_ (7.3%), C_18:1 ω6c_ (4.8%), C_20:1 ω9c_ (4.7%), C_18:2 ω6,9c_/anteiso-C_18:0_ (4.2%), C_18:1 ω7c_ (3.9%), C_14:0_ (3.7%), and anteiso-C_15:0_ (3.0%). The type strain is NM-380-WT-3C1^T^ (=DSM 105750^T^). It was isolated from faeces of a German Landrace pig in Freising, Germany. Its G+C content of genomic DNA is 35.9 mol%.

Descriptin of *Clostridium porci sp. nov*.: *Clostridium porci* (por’ci. L. gen. n. *porci* of a pig). According to 16S rRNA gene sequence analysis, the closest neighbours of this isolate are *Clostridium clostridioforme, Clostridium aldenense*, and *Clostridium citroniae* with 95.9%, 95.6%, and 95.5% sequence identity, respectively (1468 bp). All three species are placed into family *Lachnospiraceae* (phylum Firmicutes), apart from the type species of the genus *Clostridium*, *Clostridium butyricum*. ANI values between the genome of the three species aforementioned and that of the isolate were all <80%. The corresponding dDDH values were 22.8%, 22.3%, and 21.0%, respectively. These data clearly indicate the status of a separate species. Cells are rod-shaped and often occur in pairs. They grow only under strictly anoxic conditions. The genome encodes transporter systems for alkanesulfonate, arabinooligosaccharides, monosaccharides, methyl-galactoside, phosphate, aspartate, d-methionine, oligopeptide, zinc, cobalt, biotin, and teichoic acid. *N*-acetylmuramate and *N*-acetyl-d-glucosamine can be converted into *N*-acetyl-d-glucosamine-6-phosphate (EC: 2.7.1.192, 4.2.1.126 or 2.7.1.193), which can in-turn be converted into fructose-6-phosphate and enter glycolysis (EC: 3.5.1.25, 3.5.99.6). Cellobiose can also be utilized via conversion into cellobiose-6-phosphate (EC: 2.7.1.205) and subsequent conversion into d-glucose (EC: 3.2.1.86) or direct conversion into d-glucose (EC: 2.4.1.20) for use in glycolysis. Starch can also be utilized as a carbon source (EC: 2.4.1.1). The motility of this species as observed by light microscopy may be explained by the presence of many proteins involved in flagella assembly (MotB, FlhA/B, FlgB/C/G/K, FliC/D/F/G/M/N/P). After 48 h at 37 °C in modified YCFA medium under anaerobic conditions, the species consumed glucose (10.2 ± 0.6 mM) and produced 12.5 ± 0.7 mM acetate, 7.0 ± 1.1 mM formate, and trace amounts of isobutyrate (0.5 ± 0.2 mM). The major cellular fatty acids are C_16:0_ (31%) and C_18:1 ω9c_ (21%). Other fatty acids detected at lower proportions include C_14:0_ (8.7%), C_18:0_ (8.3%), C_18:1 ω7c_ (8.2%), C_16:1 ω7c_/iso-C_15:1 2OH_ (5.6%), iso-C_17:1_/anteiso B-C_17:1_ (5.0%), and anteiso-C_18:0_/C_18:2 ω6,9c_ (3.3%). The type strain is WCA-389-WT-23D1^T^ (=DSM 100959^T^). It was isolated from faeces of a German Landrace pig in Freising, Germany. Its G+C content of genomic DNA is 46.2 mol%.

Description of *Cutibacterium porci sp. nov*.: *Cutibacterium porci* (por’ci. L. gen. n. *porci* of a pig). According to 16S rRNA gene-based phylogeny, the isolate is placed within family *Propionibacteriaceae* (phylum Actinobacteria). Its closest relative is *Cutibacterium namnetense*^144^, which shares a 16S rRNA gene sequence identity of 99.0%. The status of separate species is suggested by ANI and dDDH values between the two genomes of 82.6% and 25.6%, respectively. Moreover, the difference in G+C content of the genomic DNA is 1.2%. The isolate shares 98.4% sequence identity with *Cutibacterium acnes*, the type species of the genus *Cutibacterium*. Six transporters were identified within its genome along with ABC transporter systems for phosphate, maltose/maltodextrin, d-methionine, glutathione, ribose/AI-2/D-xylose, cobalt, and biotin. Arbutin, salicin, d-glucose, sucrose, trehalose, maltose, and starch were identified as potential carbohydrate sources. Metabolism of l-aspartate into fumarate via adenylo-succinate facilitates its entry into the citrate cycle (EC: 6.3.4.4, 4.3.2.2). Conversion of sulfide into l-cysteine was identified, utilizing *O*-acetyl-l-serine produced from l-serine (EC: 2.3.1.30, 2.5.1.47). Production of riboflavin from GTP and ribulose-5-phosphate was identified (EC: 3.5.4.25, 3.5.4.26, 1.1.1.193, 3.1.3.104, 4.1.99.12, 2.5.1.78, 2.5.1.9). After 48 h at 37 °C in modified YCFA medium under anaerobic conditions, the species consumed 11.4 ± 0.8 mM glucose and produced 24.4 ± 0.3 mM propionate, 10.0 ± 0.4 mM acetate, and trace amounts of formate (0.6 ± 0.1 mM). The predominant cellular fatty acid is iso-C_15:0_ (42%). Other fatty acids included C_15:0_ (15%), iso-C_14:0 3-OH_ (10%), anteiso-C_15:0_ (7.4%), iso-C_16:0_/C_14:0 3-OH_ (7.0%), and iso-C_17:0_ (4.5%). The type strain is WCA-380-WT-3A^T^ (=DSM 101006^T^). It was isolated from faeces of a German Landrace pig in Freising, Germany. Its G+C content of genomic DNA is 58.8 mol%.

Description of *Desulfovibrio porci sp. nov*.: *Desulfovibrio porci* (por’ci. L. gen. n. *porci* of a pig). The closest phylogenetic neighbour within family *Desulfovibrionaceae* (phylum Proteobacteria, class *Deltaproteobacteria*) is *Desulfovibrio desulfuricans*, which shares a 16S rRNA gene sequence identity of 96.7%. The dDDH value of 21.4%, ANI value <80%, and difference in the G+C content of DNA of 4.2% between the two genomes confirmed the separate species status. Cells are rod-shaped and motile. A total of 52 ABC transporters were detected within the genome, including those for teichoic acid, lipoprotein, alkanesulfonate, phosphate, and d-methionine. Genes coding for the HydH/G zinc/lead two-component system were detected, suggesting a resistance to high environmental zinc and lead levels. Starch utilization may occur via conversion into *alpha*-d-glucose-1P (EC: 2.4.1.1), which can directly enter glycolysis or be converted into UDP-glucose (EC: 2.7.7.9) and then amylose (EC: 2.4.1.11). The complete dissimilatory sulfate reduction and oxidation reaction was detected (EC: 2.7.7.4, 1.8.99.2, 1.8.99.5). l-cysteine formation was identified using either sulfide (EC: 2.5.1.47) or l-serine via *O*-acetyl-l-serine (EC: 2.3.1.20, 2.5.1.47). Siroheme biosynthesis from glutamate (EC: 6..1.1.17, 1.2.1.70, 5.4.3.8, 4.2.1.24, 2.4.1.61, 2.1.1.107, 1.3.1.76) suggests that siroheme may act as a cofactor during both sulfur and nitrogen reduction. The predominant cellular fatty acids are C_15:0 ISO_ (44%) and iso-_17:1 ω9c_ (21%). Other fatty acids include iso-C_17:0_ (7.4%), C_16:0_ (7.0%), iso-C_14:0 3OH_ (3.5%), cyclo-C_17:0_ (3.0%), anteiso-C_15:0_ (2.6%), and C_18:1 ω9c_ (2.1%). The type strain is PG-178-WT-4^T^ (=DSM 105247^T^). It was isolated from faeces of a German Landrace pig in Freising, Germany. Its G+C content of genomic DNA is 61.4 mol%.

Description of *Eisenbergiella porci sp. nov*.: *Eisenbergiella porci* (por’ci. L. gen. n. *porci* of a pig). The 16S rRNA gene sequence of this species (1432 bp) shares 97.8% identity with the sequence of *Eisenbergiella tayi*, the type species of the genus *Eisenbergiella* within family *Lachnospiraceae* (phylum Firmicutes). Difference in the G+C content of genomic DNA between the two species is 1.6%. The ANI value <95% and dDDH value of 22.9% clearly favour the creation of a novel species to accommodate the isolate. Of note, a strain previously isolated by others and tentatively named ‘*Eisenbergiella massiliensis*’ shares 99.4% 16S rRNA gene sequence identity, an ANI value of 96.7% and dDDH value of 76.5% with the isolate, indicating that they belong to the same species. However, to the best of our knowledge until the time of submission of the present manuscript, ‘*E. massiliensis*’ is not a valid name. Cells grow under anaerobic conditions and form 2–5-µm-long, straight, thick rods that often grow as long chains. A single transporter was identified within the genome along with ABC transporter systems for phosphate, teichoic acid, bacitracin, osmoprotectants, maltose, d-methionine, oligo-peptides, glutathione, arabino-oligosaccharide, zinc, methyl-galactoside, cobalt, and biotin. Two-component systems for phosphate and potassium were identified along with both the BceA/B and VraD/E bacitracin efflux systems. Trehalose, starch, fructan, cellobiose, and d-glucose were identified as potential carbohydrate sources. Folate biosynthesis was identified from serine via conversion into 5,10-methylene-THMPT (EC: 2.1.2.1, 1.5.1.3). Indole biosynthesis from shikimate was identified via conversion of shikimate to chorismate (EC: 2.7.1.71, 2.5.1.19, 4.2.3.5) and choristmate to indole (EC: 4.1.3.27, 2.4.2.18, 5.3.1.24, 4.1.1.48, 4.2.1.20). Ammonia may be converted into l-glutamine (EC: 6.3.1.2) which in-turn can be utilized to form l-glutamate (EC: 1.4.1.13). The co-factor siroheme was identified to be produced from glutamate (EC: 6.1.1.17, 1.2.1.70, 5.4.3.8, 4.2.1.24, 2.5.1.61, 2.1.1.107, 1.3.1.76, 4.99.1.4). After 48 h at 37 °C in modified YCFA medium under anaerobic conditions, the species consumed 11.6 ± 0.2 mM glucose and trace amounts of propionate (0.8 ± 0.5 mM) and produced 26.3 ± 1.7 mM formate and 10.4 ± 0.1 mM butyrate. Smaller amounts of acetate (6.0 ± 4.2 mM) and traces of isobutyrate (0.5 ± 0.1 mM) were also detected. The predominant cellular fatty acid is C_18:1 ω9c_ (46%). Additional fatty acids are C_16:0_ (17.0%), C_14:0_ (11.8%), C_18:1 ω7c_ (8.9%), and C_17:1 ω9c_ (6.7%). The type strain is WCA-389-WT-23B^T^ (=DSM 101007^T^). It was isolated from faeces of a German Landrace pig in Freising, Germany. Its G+C content of genomic DNA is 48.4 mol%.

Description of *Floccifex gen. nov*.: *Floccifex* (Floc.ci.fex. L. masc. n. *floccus* a flock; L. suff. -*fex* (from L. v. facere to make); N.L. masc. n. *Floccifex* a producer of flakes, pertaining to the growth behaviour of the type strain of the type species in liquid medium). The isolate is phylogenetically placed into the family *Erysipelotrichaceae* (phylum Firmicutes). Based on 16S rRNA gene sequence analysis, it clusters apart from *Catenisphaera adipataccumulans*, with which it shares 90.5% sequence identity. The species with the closest gene sequence identity is *Faecalitalea pleomorphus* (93.0%), which is characterized by a POCP value of 53.1% to the genome of the isolate. In contrast, GTDB-Tk analysis classifies the isolate within the genus *Holdemanella*. There is no genome of *C. adipataccumulans* available to date, preventing further analyses. Considering these discrepancies based on genomes and the aforementioned 16S rRNA gene sequence identities clearly below genus delineation thresholds, we propose the creation of a novel genus, *Floccifex*, to accommodate this isolate. The type species is *Floccifex porci*.

Description of *Floccifex porci sp. nov*.: *Floccifex porci* (por’ci. L. gen. n. *porci* of a pig). Cells are irregular rods of ca. 1 µm in length that form short to long chains. After 7–14 days of growth in BHI broth at 37 °C under anaerobic conditions, the species forms characteristic amalgams of cells that appear as flakes floating in the liquid medium. Within the genome, 18 transporters were detected along with ABC transporter systems for phosphate, teichoic acid, d-methionine, oligopeptides, and biotin. The CssS/R, HtrA two-component system for detection and removal of misfolded proteins was present. d-glucose, arbutin, salicin, fructan, starch, cellobiose, maltose, and trehalose were all identified as potential carbohydrate substrates for this species. Production of ornithine, valine, and leucine was identified from glutamate, pyruvate, and 2-oxoisocaproate, respectively. Metabolism of l-aspartate into fumarate allowed for entry into the citrate cycle. Glutamate biosynthesis was identified from ammonia (EC: 6.3.1.2, 1.4.7.1), which itself was produced from nitrile (EC: 3.5.5.1). Main cellular fatty acids are C_18:1 ω9c_ (23%), iso-anteiso-C_17:1_ (18%), and C_16:0_ (16.3%). Other fatty acids were detected in lower proportions: C_18:2 ω6,9c_/anteiso-C_18:0_ (9.4%), C_18:0_ (8.5%), and iso-C_17:0 3-OH_ (8.1%). The type strain is LKV-178-WT-2G^T^ (=DSM 104670^T^). It was isolated from faeces of a German Landrace pig in Freising, Germany. Its G+C content of genomic DNA is 33.5 mol%.

Description of *Hallerella gen. nov*.: *Hallerella* (Hal.ler.el’la. N.L. dim. fem. n. *Hallerella* named in honour of Prof. Dirk Haller from the Technical University of Munich, Germany, for his contribution to the field of microbe–host interactions in the gut). The nearest phylogenetic neighbour of these strictly anaerobic bacteria within the family *Fibrobacteriaceae* (phylum *Fibrobacteres*) is *Fibrobacter intestinalis*. The POCP values >70% between the two isolated strains and *F. intestinalis* clearly indicate the status of these three bacteria as members of the same genus. The POCP value between the genome of *F. intestinalis* and that of *Fibrobacter succinogenes*, the type species of the genus *Fibrobacter*, is 52.1%, which is nearing the genus-specific delineation threshold. Although the isolates showed POCP values of 54.7 and 55.8% to *F. succinogenes*, GTDB-Tk proposed a genus distinct from *Fibrobacter*. Moreover, 16S rRNA gene sequence identities between the two isolates and *F. succinogenes* are <92%, well below established genus delineation thresholds. According to these findings, we suggest that a novel genus distinct from *Fibrobacter* must be created to accommodate the two isolated strains and that *F. intestinalis* may have to be re-classified within this genus, for which the name *Hallerella* is proposed. The type species is *Hallerella porci*.

Description of *Hallerella porci sp. nov*.: *Hallerella porci* (por’ci. L. gen. n. *porci* of a pig). The species shares 94.2% 16S rRNA gene sequence identity with *F. intestinalis* and <91% identity with *F. succinogenes*, the type species of the genus *Fibrobacter*. The species grows as straight rods of 1–2 µm in length that occur as single cells. It grows best on solid media. Within the genome, 11 transporters were identified along with ABC transporter systems for phosphate, alkanesulfonate, teichoic acid, and lipoproteins. Starch and cellulose were identified as potential carbohydrate substrates. Both the complete pathway from pyruvate to valine and to leucine via 2-oxoisovalerate were identified. Conversion of folate to and from both 7,8-dihydrofolate and 5,6,7,8-tetrahydrofolate was also identified (EC: 1.5.1.3). After 96 h at 37 °C in modified YCFA medium under anaerobic conditions, the species consumed glucose (13.1 ± 0.4 mM) and produced succinate (10.4 ± 0.5 mM), acetate (5.3 ± 0.6 mM), formate (2.5 ± 0.3 mM), and traces of isobutyrate (0.7 ± 0.1 mM). The main cellular fatty acid is C_16:0_ (40%). Other cellular fatty acids include C_14:0 2-OH_ (17.9%), C_15:0_ (12.4%), iso-C_13:0 3-OH_ (10.6%), and iso-C_16:0_ (4.8%). The type strain is UWS4^T^ (=DSM 104699^T^). It was isolated from the caecum of a pig in Madison, Wisconsin, USA. Its G+C content of genomic DNA is 44.8 mol%.

Description of *Hallerella succinigenes sp. nov*.: *Hallerella succinigenes* (suc.ci.ni’ge.nes. N.L. neut. n. *acidum succinum* succinic acid; N.L. suff. -*genes* (from Gr. v. *gennaiô* to produce) producing; N.L. part. adj. *succinigenes* succinic acid-producing). The species shares 97.0% 16S rRNA gene sequence identity with *F. intestinalis*, 95.0% with *H. porci*, the type species of the proposed genus *Hallerella*, and 91.5% with *F. succinogenes*, the type species of the genus *Fibrobacter*. The dDDH and ANI values of 22.8% and 80.2% to *H. porci* as well as 21.8% and <80% to *F. intestinalis* confirm the status of the isolate as a separate species. Cells are tiny rods that grow only under strictly anoxic conditions. Within the genome, 14 transporters along with transporter systems for alkanesulfonate, zinc, phosphonate, phosphate, teichoic acid, Na^+^, and lipoprotein were found. Cellulose was predicted to be converted into d-glucose and utilized in glycolysis via conversion into cellobiose (EC: 3.2.1.4) and then d-glucose (EC: 2.4.1.20). Starch may also be utilized as a carbon source (EC: 2.4.1.1). Pyruvate may be utilized to produce either valine (EC: 2.2.1.6, 1.1.1.86, 4.2.1.9, 2.6.1.42) or leucine (EC: 2.2.1.6, 1.1.1.86, 4.2.1.9, 2.3.3.13, 4.2.1.33, 1.1.1.85, 2.6.1.42). DAP-type peptidoglycan production was predicted to occur from UDP-*N*-acetyl-*alpha*-d-glucosamine (EC: 2.5.1.7, 1.3.1.98, 6.3.2.8, 6.3.2.9, 6.3.2.13, 6.3.2.10, 2.7.8.13, 2.4.1.227, 2.4.1.129, 3.4.16.4). Propanoate production was identified to be formed from propanoyl-CoA via propanoyl-phosphate (EC: 2.3.1.8, 2.7.2.15) or propionyl-adenylate (EC: 6.2.1.1). After 48 h at 37 °C in modified YCFA medium under anaerobic conditions, the species consumed glucose (11.5 ± 0.5 mM) and produced succinate (8.2 ± 1.9 mM), acetate (3.2 ± 1.9 mM), and small amounts of formate (0.9 ± 0.1 mM). The cellular fatty acids profile consists of iso-C_13:0 3-OH_ (24%), C_14:0 2-OH_ (24%) and C_15:0_ (18%), C_16:0_ (13%), iso-C_16:0_ (6.2%), and anteiso-C_15:1_ (5.9%). The type strain is UWS3^T^ (=DSM 104698^T^). It was isolated from the caecum of a pig in Madison, Wisconsin, USA. Its G+C content of genomic DNA is 49.2 mol%.

Description of *Holdemanella porci sp. nov*.: *Holdemanella porci* (por’ci. L. gen. n. *porci* of a pig). The closest phylogenetic neighbour within family *Erysipelotrichaceae* (phylum Firmicutes) is *Holdemanella biformis*, the type species of the genus, which shares a 16S rRNA gene sequence identity of 98.4%. The dDDH and ANI value of 39.8% and 90.1%, respectively, confirm the separate species status. The species is strictly anaerobic and grows as bacilli that stain Gram-positive and form chains of up to 10 µm. A total of 18 transporters along with transporter systems for phosphate, d-methionine, and teichoic acid were detected in the genome. The two-component system CssRS for removal of misfolded proteins along with the HtrA serine protease were also detected. Cellobiose was suggested to be an energy source via conversion into cellobiose-6-phosphate (EC: 2.7.1.205) and subsequent conversion into d-glucose (EC: 3.2.1.86). Extracellular trehalose can also be utilized via conversion to trehalose-6-phosphate (EC: 2.7.1.201) and then to d-glucose-6-phosphate (EC: 3.2.1.122). Arbutin and salicin can be converted into *beta*-d-glucose-6-phosphate (EC: 2.7.1.-, 3.2.1.86) which then enters glycolysis via conversion into *beta*-d-fructose-6-phosphate (EC: 5.3.1.9). Starch can also be utilized as a carbon source (EC: 2.4.1.1). Ammonia was identified to be utilized for production of l-glutamate (EC: 1.4.1.13, 1.4.7.1) via l-glutamine (EC: 6.3.1.2). Based on the analysis of a large catalogue of metagenome-reconstructed genomes, the species is proposed to be a prevalent member of the intestinal microbiome also in human. The cellular fatty acid profile comprises C_18:1 ω9c_ (35%), C_16:0_ (20%), C_18:0_ (15%) as major components. Minor fatty acids included C_18:2 ω6,9c_/anteiso-C_18:0_ (6.9%), iso-C_17:1_/anteiso-C_17:1_ (4.0%), C_16:1 ω6,9c_/iso-C_15:0 2OH_ (3.5%), and C_18:1 ω7c_ (3.2%). The type strain is LKV-472-APC-3^T^ (=DSM 105256^T^). It was isolated from faeces of an APC^1311/+^ pig in Freising, Germany. Its G+C content of genomic DNA is 34.1 mol%.

Description of *Hornefia gen. nov*.: *Hornefia* (Hor.nef“i.a. N.L. fem. n. *Hornefia* named in honour of Prof. Mathias Hornef from the RWTH University Hospital in Aachen, Germany, for his contribution to the field of enteric infections in neonates). Based on 16S rRNA gene sequence analysis, the closest phylogenetic neighbour for both isolates is *Eubacterium nodatum* (<93.5% sequence identity). They are placed within the yet undescribed family ‘*Mogibacteriaceae*’, distant from the type species of the type genus within family *Eubacteriaceae*, *Eubacterium limosum*. With POCP values of ca. 24 and 53% against the genome of *E. limosum* and *E. nodatum* together with a POCP value of 36–38% against *Mogibacterium pumilum*, the type species of the genus *Mogibacterium*, the isolates can be considered to represent a novel genus, which was confirmed by GTDB-Tk classification. Both isolates grow only under strictly anaerobic conditions. Genome features that are consistent across species of this genus include: (i) the production of *O*-acetyl-l-homoserine and *O*-succinyl-l-homoserine from l-aspartate; (ii) the conversion of CO_2_ into formate; (iii) starch was identified as the sole consistent carbohydrate source; (iv) ABC transport systems for phosphate, teichoic acid, osmoprotectants, cobalt, and biotin; (v) the AtoA/B/C/D/E/S two-component system which detects acetoacetate and leads to SCFA metabolism; (vi) the SCFAs butyric, acetic, and propionic acid, were all identified as products; (vii) propionic acid production occurs from acryloyl-CoA (EC: 1.3.8.1, 2.3.1.8/2.3.1.222, 2.7.2.1), acetic acid from acetyl-CoA (EC: 2.3.1.8, 2.7.2.1) and butyric acid from butanal (EC: 1.2.1.10, 2.8.3.8). The genus name *Hornefia* is proposed to accommodate both isolates. The type species is *Hornefia butyriciproducens*.

Description of *Hornefia butyriciproducens sp. nov*.: *Hornefia butyriciproducens* (bu.ty.ri.ci.pro.du’cens. N.L. neut. n. *acidum butyricum* butyric acid; L. pres. part. *producens* producing; N.L. part. adj. *butyriciproducens* producing butyric acid). This species shares all the functional features associated with the genus. Cells are irregular rods with a length of 1–3 µm. Whilst both species of the genus are capable of producing *O*-acetyl-l-homoserine and *O*-succinyl-l-homoserine from l-aspartate, only *H. butyriciproducens* is able to convert l-aspartate into 2-ocobutanoate using l-homoserine as an intermediate (EC: 2.7.2.4, 1.2.1.11, 1.1.1.3, 2.3.1.46, 2.5.1.48), which can then be utilized for production of propionic acid (EC: 2.3.1.54, 2.3.1.8/2.3.1.222, 2.7.2.1). *H. butyriciproducens* is also able to utilize sucrose. The species is considered to deconjugate primary bile acids as determined in vitro by cleavage of taurine residues on WCA agar medium containing TDCA. After 48 h at 37 °C in modified YCFA medium under anaerobic conditions, the species consumed traces of acetate (1.4 ± 1.1 mM) and produced 6.0 ± 1.8 mM butyrate and small amounts of formate (1.3 ± 0.4 mM) and succinate (0.4 ± 0.2 mM). Cellular fatty acids mostly consist of C_16:0_ (26%), iso-C_17:1_/anteiso-C_17:0_ (17%), iso-C_19:1_ (16%), and C_16:1 ω7c_/iso-C_15:0 2OH_ (10%). Minor fatty acids include C_14:0_ (8.6%), C_18:1 ω7c_ (4.8%), C_18:0_ (4.1%), and C_18:1 ω7c_ (3.7%). The type strain is WCA-MUC-591-APC-3H^T^ (=DSM 104962^T^). It was isolated from faeces of an APC^1311/+^ pig in Freising, Germany. Its G+C content of genomic DNA is 51.6 mol%.

Description of *Hornefia porci sp. nov*.: *Hornefia porci* (por’ci. L. gen. n. *porci* of a pig). This species shares 99.3% sequence identity with the type species of the genus, *H. butyriciproducens*, according to their the 16S rRNA genes. However, their dDDH and ANI values of 49.4% and 92.8% indicate a separate species status, whilst the POCP value of 81.5% confirms their classification within the same genus. This species possesses all functional features of the genus. It is able to utilize CO_2_ for the production of both CO (EC: 1.2.7.4) and formate (EC: 1.17.1.10). The species is considered to deconjugate primary bile acids as determined in vitro by cleavage of taurine residues on WCA agar medium containing TDCA. After 48 h at 37 °C in modified YCFA medium under anaerobic conditions, the species consumed traces of acetate (1.9 ± 0.7 mM) and produced 5.6 ± 0.4 mM butyrate and 2.2 ± 0.3 mM formate. Compared with *H. butyriciproducens*, cells of *H. porci* contain higher amounts of the cellular fatty acid iso-C_17:1_/anteiso-C_17:0_ (31%) at the expense of iso-C_19:1_ (8.7%), C_14:0_ (5.4%), and C_16:1 ω7c_/iso-C_15:0 2OH_ (4.34%). C_17:1 ω8c_ (3.7%) was detected only in *H. porci*. The type strain is 68-3-10^T^ (=DSM 104948^T^). It was isolated from the colon content of a pig in Ames, Iowa, USA. Its G+C content of genomic DNA is 52.3 mol%.

Description of* Inconstantimicrobium gen. nov*.: *Inconstantimicrobium* (In.con.stan.ti.mi.cro’bi.um. L. masc. adj. *inconstans* changeable, inconsistent; N.L. neut. n. *microbium* a microbe; N.L. neut. n. *Inconstantimicrobium* a microbe with variable growth behaviour). The genus is phylogenetically placed into the family *Clostridiaceae* (phylum Firmicutes) based on 16S rRNA gene analysis. *Clostridium sardiniense* is the closest relative, with a sequence identity of 94.4%. The isolate clusters away from *Clostridium butyricum*, the type species of the genus *Clostridium*, with which it shares 93.4% sequence identity. There is no genome available for *C. sardiniense*, which prevents further analysis. The POCP values of 45.6% between the genome of the isolate and that of *C. butyricum* confirm a genus status distinct from *Clostridium*, for which the name *Inconstantimicrobium* is proposed. The type species is *Inconstantimicrobium porci*.

Description of *Inconstantimicrobium porci sp. nov*.: *Inconstantimicrobium porci* (por’ci. L. gen. n. *porci* of a pig). The species has all the features of the genus. Cells are motile, thick rods with a length of 2–10 µm. They have a very characteristic swarming growth behaviour on solid media. They grow only under strictly anoxic conditions and stain Gram-positive. Within the genome, a single transporter was identified along with ABC transporter systems for phosphate, Na+, bacitracin, d-methionine, zinc, and biotin. Sucrose, cellobiose, maltose, trehalose, starch, arbutin, salicin, and d-glucose were identified as potential carbohydrate sources. Motility may be conferred by flagella as indicated by the presence of the following genome-encoded proteins: FliA/C/D/E/F/G/M/N/S/P, FlhA/B, FlgB/C/E/G/K, and MotB. Motility direction is predicted to be governed via the CheA/B/C/D/R/V/W/Y/X and MCP genes. Glycogen production via GlgA/C/P along with quorum sensing genes including LuxS may indicate that this species is able to form biofilm. The major cellular fatty acid is C_18:1 ω9c_ (86%). Smaller proportions of C_18:1 ω7c_ (6.0%) and C_18:1_ (3.5%) were detected. The type strain is WCA-383-APC-5B^T^ (=DSM 108839^T^). It was isolated from faeces of an APC^1311/+^ pig in Freising, Germany. Its G+C content of genomic DNA is 31.4 mol%.

Description of *Mobiluncus porci sp. nov*.: *Mobiluncus porci* (por’ci. L. gen. n. *porci* of a pig). The closest phylogenetic neighbour within family *Actinomycetaceae* (phylum Actinobacteria) is *Mobiluncus curtisii*, the type species of the genus *Mobiluncus*, which shares a 16S rRNA gene sequence identity of 98.6%. The dDDH value of 20.6% and ANI value <80% between the two genomes indicate that *M. porci* is a novel species. Cells are motile, rod-shaped, curved, and 2–4 µm long. Twenty transporters along with transporter systems for d-methionine, phosphate, and teichoic acid were detected in the genome. The phosphate detection and assimilation two-component system SenX3/RegX3 along with the *phoA* and *pstS* genes were also detected. Starch seems to be the only extracellular carbon source (EC: 2.4.1.1) utilized by this species, apart from d-glucose, both of which enter glycolysis. Sulfide and l-serine can be converted into l-cysteine (EC: 2.3.1.30, 2.5.1.47) or converted into sulfur, releasing hydrogen (EC: 1.8.2.3). Propanoate production was identified to occur via the utilization of propanoyl-CoA via propanoyl-phosphate (EC: 2.3.1.8, 2.7.2.1). The motility of this species may be due to the presence of flagella as multiple flagella assembly genes were identified within the genome (*motB, flhA/B, flgB/C/E/K, fliA/C/F/G/N/M/S/P*). The major cellular fatty acids are C_16:0_ (22%), C_18:0_ (15%), and C_18:1 ω9c_ (14%). Other fatty acids included C_12:0_ (8.5%), iso I-C_19:1_ (4.3%), C_18:1 ω6c_ (3.9%), C_14:0_ (3.7%), C_18:2 ω6,9c_/anteiso-C_18:0_ (3.2%), C_17:1 ω9c_ (2.9%), C_16:1 ω7c_/iso-C_15:0 2OH_ (2.8%), iso I/anteiso B-C_17:1_ (2.4%), C_13:0 3OH_/C_15:1 i I/H_ (2.4%), and C_10:0_ (2.2%). The type strain is RF-GAM-744-WT-7^T^ (=DSM 108840^T^). It was isolated from faeces of an Aachen minipig^83^ in Germany. Its G+C content of genomic DNA is 56.3 mol%.

Description of *Mogibacterium kristiansenii sp. nov*.: *Mogibacterium kristiansenii* (kris.ti.an.se’ni.i. N.L. gen. n. *kristiansenii* named in honour of Prof. Karsten Kristiansen from the University of Copenhagen, Denmark, for his contribution to the field of microbiome research and metagenomics). Based on 16S rRNA gene sequence analysis, the nearest phylogenetic neighbour is *Mogibacterium diversum*, a member of the yet undescribed family ‘*Mogibacteriaceae*’ within the phylum Firmicutes (order *Clostridiales*), which shares 94.4% sequence identity. Analysis of the two genomes revealed a POCP value of 52.0% and GTDB-Tk classified the isolate as member of the genus *Mogibacterium*. The difference in G+C content of genomic DNA of 9.1 mol% together with ANI and dDDH values of <80% and 20.4%, respectively, clearly indicate the status of a separate species. Cells are 1–1.5 µm long, thin rods that grow only under strictly anoxic conditions and stain Gram-positive. Within the genome, four transporters were identified along with ABC transporter systems for phosphate, teichoic acid, and biotin. No carbohydrate sources were identified to be used by this species. However, glucose-6-phosphate utilization within glycolysis was present, suggesting an alternative method for the production of energy. Formate production from CO_2_ (EC: 1.17.1.10) and subsequent conversion into pyruvate via serine may be one way by which this species produces energy (EC: 6.3.4.3, 1.5.1.5/3.5.4.9, 2.1.2.1, 4.3.1.17). The most dominant cellular fatty acid is C_18:1 ω9c_ (64%). Lower proportions of other fatty acids were measured: C_16:0_ (10.2%), C_18:1 ω6c_ (5.7%), C_18:0_ (5.2%), and C_17:1 ω9c_ (4.1%) could be detected as well. The type strain is WCA-MUC-591-APC-4B^T^ (=DSM 106282^T^). It was isolated from faeces of an APC^1311/+^ pig in Freising, Germany. Its G+C content of genomic DNA is 50.0 mol%.

Description of *Oliverpabstia gen. nov*.: *Oliverpabstia* (O.li.ver.pabst’i.a. N.L. fem. n. *Oliverpabstia* named in honour of Prof. Oliver Pabst from the RWTH University in Aachen, Germany, for his contribution to the field of mucosal immunology). The genus is phylogenetically placed into the family *Lachnospiraceae* (phylum Firmicutes). Based on 16S rRNA sequence identity, *Blautia stercoris* is the closest phylogenetic neighbour (94.3%). The corresponding ANI and dDDH values of <80% and 24.5% confirm that the isolate represents a different taxon. The POCP value between the two genomes is 54.9%. However, GTDB-Tk places the isolates into a new genus within the *Lachnospiraceae* and the 16S rRNA gene sequence identity to *Blautia coccoides*, the type species of this genus, is 92.7%. Hence, we propose the name *Oliverpabstia* for this novel genus. The type species is *Oliverpabstia intestinalis*.

Description of *Oliverpabstia intestinalis sp. nov*.: *Oliverpabstia intestinalis* (in.tes.ti.na’lis. N.L. fem. adj. *intestinalis* pertaining to the gut). Cells are strictly anaerobic coccobacilli with conic ends that stain Gram-positive and often grow in pairs. The genome encodes two transporters along with ABC transporter systems for phosphate, teichoic acid, arabino-oligosaccharide, and biotin. The RpfC/G, Clp two-component system for biofilm production was detected within the genome, along with LuxS for quorum signalling. Indole biosynthesis from shikimate was identified via conversion of shikimate to chorismate (EC: 2.7.1.71, 2.5.1.19, 4.2.3.5) and choristmate to indole (EC: 4.1.3.27, 2.4.2.18, 5.3.1.24, 4.1.1.48, 4.2.1.20). The species is considered to deconjugate primary bile acids as determined in vitro by cleavage of taurine residues on WCA agar medium containing TDCA. The major cellular fatty acid is C_16:0_ (34%). Other fatty acids are C_14:0_ (16.6%), C_18:1 ω7c_ (15.6%), iso/anteiso-C_17:1_ (8.8%), C_13:0 3OH_/C1_5:1 I I/H_ (4.0%), and C_16:1 ω7C_/iso-C_15:1 2OH_ (3.3%). The type strain is BSM-380-WT-5A ^T^ (=DSM 106162^T^). It was isolated from faeces of a German Landrace pig in Freising, Germany. Its G+C content of genomic DNA is 44.1 mol%.

Description of *Olsenella porci sp. nov*.: *Olsenella porci* (por’ci. L. gen. n. *porci* of a pig). The closest phylogenetic neighbour within family *Atopobiaceae* (phylum Actinobacteria) is *Olsenella uli*, the type species of the genus *Olsenella*, which shares a 16S rRNA gene sequence identity of 96.7% (1463 bp). The dDDH value of 20.8% and ANI value <80% between the two genomes confirm that *O. porci* is a novel species. The bacterium grows as irregular rods with a length of 0.5–1 µm that may form chains. Eight transporters along with transporter systems for phosphate, aspartate/glutamate/glutamine, and cystine were present in the genomes. Starch, cellobiose, maltose, arbutin, salicin, and d-glucose were identified as potential substrates. l-cysteine may be converted into pyruvate (EC: 4.4.1.13), potentially acting as a source of energy. l-cysteine can also be produced internally using sulfide and acetyl-l-serine (EC: 2.5.1.47). Additionally, l-aspartate may also enter the TCA cycle via conversion into oxaloacetate (EC: 2.6.1.1) or to fumarate using either adenylo-succinate (EC: 6.3.4.4, 4.3.2.2) or l-arginino-succinate (EC: 6.3.4.5, 4.3.2.1) as intermediates. The TCA cycle itself is incomplete with the steps from 2-oxo-glutarate to fumarate and the conversion of malate to oxaloacetate missing. The species is considered to deconjugate primary bile acids as determined in vitro by cleavage of taurine residues on WCA agar medium containing TDCA. After 96 h at 37 °C in modified YCFA medium under anaerobic conditions, no glucose was utilized and only small amounts of acetate (1.9 ± 0.2 mM) were detected. The predominant cellular fatty acid is C_18:1 ω9c_ (33%). Other fatty acids include C_12:0_ (11.2%), iso-C_19:1_ (10.6%), C_16:0_ (9.6%), C_18:2 ω6/9c_/anteiso-C_18:0_ (6.6%), C_18:0_ (6.4%), C_18:1 ω7c_ (5.8%), C_17:1 ω9c_ (3.7%), iso/anteiso-C_17:1_ (3.3%), and C_16:1 ω7c_/iso-C_15:0 2-OH_ (2.3%). The type strain is CA-Schmier-601-WT-1^T^ (=DSM 105246^T^). It was isolated from faeces of a German Landrace pig (minipig breed) in Freising, Germany. Its G+C content of genomic DNA is 64.9 mol%.

Description of *Peptoniphilus porci sp. nov*.: *Peptoniphilus porci* (por’ci. L. gen. n. *porci* of a pig). Based on 16S rRNA gene sequence analysis, the closest phylogenetic neighbour with a valid name is *Peptoniphilus gorbachii* within family *Peptoniphilaceae* (phylum Firmicutes), with a sequence identity of 97.1% (1412 bp). There is no genome available for *P. gorbachii*, preventing further analysis. Sequence identity to the next relative, *Peptoniphilus harei*, is 96.0% and to *Peptoniphilus asaccharolyticus*, the type species of the genus, 88.5%. The latter species is characterized by a POCP value of 55.2 to the isolate, suggesting that they indeed belong to the same genus despite the low 16S rRNA gene sequence identity outlined above. The ANI/dDDH values of 81.1%/20.6% (isolate to *P. harei*) and <80%/33.1% (isolate to *P. asaccharolyticus*) together with corresponding differences in the G+C content of genomic DNA of 0.9 mol% and 3.2 mol% confirm the status of our isolate as a new species. Of note, due to an ANI value >95%, the isolate is proposed to belong to the same species as strain ph5 (= DSM 25475), previously isolated by others from human faeces^145^. However, by the time of submission of this manuscript, the proposed name of the latter isolate, ‘Peptinophilus grossensis’, is still not valid. Moreover, the two isolates share only 96.9% 16S rRNA gene sequence identity. Hence, we think it is appropriate to propose a novel species to accommodate this pig isolate. Altogether, *Peptoniphilus* appears to be a phylogenetically and phylogenomically heterogeneous genus that may require taxonomic rearrangement in the future. Cells of this new species are cocci that stain Gram-positive and grow single or in pairs. Within the genome, a single transporter was identified; however, transporter systems for osmoprotectants, zinc, biotin, d-methionine, and phosphate were present. A two-component system for phosphate limitation was detected, which consists of PhoR, PhoB, PhoA, and PhoP. A distinct lack of carbohydrate metabolism genes was noticed with no extracellular carbohydrate sources being utilized. Alternatively, it appeared that both d-serine and l-serine may act as an energy source via conversion into pyruvate and ammonia (EC: 4.3.1.18 and 4.2.1.17, respectively). Pyruvate can then be converted into acetyl-CoA (EC: 1.2.7.11) that can be used to produce acetate (EC: 1.2.1.10, 1.2.1.3). Propanoate production was also identified from acryloyl-CoA via conversion to propanoyl-CoA (EC: 1.3.8.1), propanoyl phosphate (EC: 2.3.1.8, 2.3.1.222) and then propanoate (EC: 2.7.2.1). After 48 h at 37 °C in modified YCFA medium under anaerobic conditions, the species did not consume glucose and produced 9.5 ± 0.5 mM acetate, 3.5 ± 0.2 mM butyrate, 3.0 ± 0.5 mM propionate, and smaller amounts of formate (0.6 ± 0.2 mM). The dominant cellular fatty acids are: C_16:0_ (28%), C_18:1 ω7c_ (15%), C_14:0_ (13%), iso/anteiso-C_17:1_, and C_16:1 ω7c_/iso-C_15:1 2OH_ (12% each). Lower proportions of the following fatty acids were detected: C_16:1 ω9c_ (5.7%), C_18:0_ (2.2%), and C_16:1 ω5c_ (2.1%). The type strain is 35-6-1^T^ (=DSM 104947^T^). It was isolated from the colonic mucosa of a pig in Ames, Iowa, USA. Its G+C content of genomic DNA is 31.2 mol%.

Description of *Peptostreptococcus porci sp. nov*.: *Peptostreptococcus porci* (por’ci. L. gen. n. *porci* of a pig). The closest phylogenetic neighbours within family *Peptostreptococcaceae* (phylum Firmicutes) are *Peptostreptococcus anaerobius*, the type species of the genus *Peptostreptococcus*, which shares a 16S rRNA gene sequence identity of 98.4% and *Peptostreptococcus canis* (97.9% sequence identity). Whilst there is no genome available for *P. canis*, the dDDH value of 22.6%, ANI value <80%, and difference in the G+C content of genomic DNA (2.4%) between the genomes of our isolate and *P. anaerobius* support a separate species. Cells are irregular bacilli with a length of 0.5–1 µm. They stain Gram-positive and are strictly anaerobic. Within the genome, two transporters were identified along with ABC transporter systems for phosphate, osmoprotectants, zinc, biotin, cobalt, and d-methionine. Both the *arlS* and *arlP* genes were detected, suggesting a potential two-component system for adhesion. Starch was identified as the sole carbohydrate source. Alternatively, l-serine and sulfide may act as an energy source via conversion into acetate and l-cysteine (EC: 2.5.1.47). l-serine may first be converted into *O*-acetyl-l-serine (EC: 2.5.1.47). Acetate can be converted into acetyl-CoA (EC: 2.7.2.1, 2.3.1.8), which can be used for fatty acid synthesis. Propanoate production was also identified from acryloyl-CoA via conversion to propanoyl-CoA (EC: 1.3.8.1), propanoyl phosphate (EC: 2.3.1.8, 2.3.1.222) and then propanoate (EC: 2.7.2.1). Whilst production of butanoyl-P and butanoate was not identified, the ability to convert one to the other was present (EC: 2.7.2.7). After 48 h at 37 °C in modified YCFA medium under anaerobic conditions, the species consumed glucose (2.2 ± 4.9 mM) and propionate (1.5 ± 2.1 mM) and produced 15.4 ± 2.0 mM acetate, and smaller amounts of isobutyrate (3.1 ± 0.8 mM), isovalerate (3.0 ± 1.3 mM), formate (1.7 ± 0.6 mM), and lactate (1.7 ± 0.3 mM). The cellular fatty acid composition of *P. porci* is dominated by iso-C_16:0_ (20%) and iso-C_14:0_ (10%). Other fatty acids include iso-C_17:1_/anteiso-C_17:1_ (10.3%), iso-C_12:0_ (6.5%), iso-C_18:0_ (5.4%), iso-C_13:0 3-OH_ (4.0%), and iso-C_17:0_ (3.6%). The type strain is WCA-SAB-591-4A-A^T^ (=DSM 106284^T^). It was isolated from faeces of a German Landrace pig (minipig breed) in Freising, Germany. Its G+C content of genomic DNA is 33.3 mol%.

Description of *Porcincola gen. nov*.: *Porcincola* (Porc.in’co.la. L. masc. n. *porcus* a pig; L. masc. n. *incola* inhabitant, dweller; N.L. masc. n. *Porcincola* a dweller of pigs, pertaining to the origin of the type species). Phylogenetically, the isolate falls into family *Lachnospiraceae* (phylum Firmicutes). The closest neighbour based on the comparison of partial 16S rRNA gene sequences (1438 bp) is *Blautia producta*, which shares 90.0% sequence identity. The isolate also shares 89.2% identity with *Marvinbryanta formatexigens*, the type species of the genus *Marvinbryanta* within the same family. The POCP values between the genome of the isolate and that of these two related species are 29.3% and 36.3%, respectively, which confirms its separate genus status. Cells grow only under strictly anaerobic conditions. The type species is *Porcincola intestinalis*.

Description of *Porcincola intestinalis sp. nov*.: *Porcincola intestinalis* (in.tes.ti.na’lis. N.L. masc. adj. *intestinalis* pertaining to the gut). Cells are coccobacilli that grow in pairs and can form chains up to 10 µm in length. They stain Gram-positive and are non-motile. Two transporters were identified within the genome along with ABC transporter systems for phosphate, cystine, teichoic acid, d-methionine, oligopeptide, arabino-oligosaccharide, ribose, AI-2, d-xylose, methyl-galactoside, biotin, and zinc. Presence of the RbsA/B/C ABC transporter for AI-2 and LuxB/D/S suggests this species may utilize AI-2 as a quorum sensing molecule. Starch and cellulose were identified to be the only carbohydrate sources utilized by this species. An alternative pathway for energy production is conversion of glutamate into ornithine (EC: 2.3.1.1, 2.7.2.8, 1.2.1.38, 2.6.1.11, 2.3.1.35) that can enter the urea cycle, thereby producing fumarate (EC: 2.1.3.3, 6.3.4.5, 4.3.2.1). The species is considered to deconjugate primary bile acids as determined in vitro by cleavage of taurine residues on WCA agar medium containing TDCA. It is also positive for lipase production using the Rhodamine B agar assay. The predominant cellular fatty is C_18:1 ω9c_ (65%). Lower amounts of C_16:0_ (13.7%), C_18:1 ω7c_ (7.9%), and iso/anteiso-C_17:1_ (3.5%) were detected. The type strain is Oil+RF-744-WCA-WT-11^T^ (=DSM 106895^T^). It was isolated from faeces of an Aachen minipig^83^ in Germany. Its G+C content of genomic DNA is 52.0 mol%.

Description of *Prevotella mizrahii sp. nov*.: *Prevotella mizrahii* (miz.rah’i.i. N.L. gen. n. *Mizrahia* named in honour of Prof. Itzhak Mizrahi from the Ben Gurion University of the Negev, Israel, for his contribution to the field of rumen microbiology). According to 16S rRNA gene-based phylogeny, the isolate falls into the family *Prevotellaceae* (phylum Bacteroidetes). The neighbouring species with the highest sequence identity is *Prevotella scopos* (91.5%; 1493 bp). Identity to *Prevotella melaninogenica*, the type species of the genus *Prevotella*, is 90.9%. Additionally, the isolate branches together with *Hallella seregens*, the type species of this genus, which shares 91.2% identity and was previously proposed to be reclassified within the genus *Prevotella*^146^; however, this proposal still has no standing in the nomenclature. The POCP values between the genome of our strain and that of the species aforementioned are 47.8% (*P. scopos*), 50.8% (*P. melaninogenica*), and 55.4% (*H. seregens*), which oscillate around the proposed genus-level threshold of 50%. GTDB-Tk places the isolate within the genus *Prevotella*. Considering that: (i) these findings on the genus delineation of this isolate are altogether somewhat contradictory (e.g. 16S rRNA gene-based phylogeny and phylogenomic analysis clearly place the isolate within the *Prevotella* spp. cluster as per current validity of taxonomic names; however, proposing a novel species within an existing genus when 16S rRNA gene sequence identities are ≤91.5% seems not appropriate); and (ii) *Prevotella* appears to be a phylogenetically and phylogenomically heterogeneous genus that will require taxonomic rearrangement in the near future, we propose the creation of a novel species only, named *Prevotella mizrahii*, to avoid generating even more instability within this genus. Cells are thick rods (0.5–1.5 µm) that can be slightly curved. Growth is good (high turbidity) in anoxic WCA but the strain cannot be maintained in culture unless passaged every second day. Within the genome, one transporter along with ABC transporter systems for phosphate and lipoproteins were identified. Fructan, cellulose, starch, and sucrose are potential carbohydrate substrates. Riboflavin biosynthesis was predicted from GTP and ribulose-5-phosphate (EC: 3.5.4.25, 3.5.4.26, 1.1.1.193, 3.1.3.104, 4.1.99.12, 2.5.1.78, 2.5.1.9). Extracellular nitrite and nitrate were identified to be transported internally via Nrt and then converted into ammonia (EC: 1.7.2.2) and further to l-glutamate (EC: 6.3.1.2, 1.4.7.1, 1.4.1.13). Based on metagenome-based functional predictions, this species is proposed to be a key functional member of pig microbiomes. It is considered to deconjugate primary bile acids as determined in vitro by cleavage of taurine residues on WCA agar medium containing TDCA. After 48 h at 37 °C in modified YCFA medium under anaerobic conditions, the species consumed glucose (10.1 ± 0.3 mM) and produced 16.8 ± 1.4 mM succinate and 10.3 ± 0.6 mM acetate. Small amounts of formate (3.0 ± 0.3 mM) and isobutyrate (0.6 ± 0.1 mM) were also detected. The most abundant cellular fatty acid is anteiso-C_15:0_ (24.4%). Other fatty acids are C_16:0 3-OH_ (12.5%), C_16:0_ (11.4%), iso-C_17:0 3-OH_ (11.4%), iso-C_15:0_ (8.9%), iso/anteiso-C_17:1_ (8.9%), iso-C_17:0_ (3.9%), anteiso-C_17:0_ (3.2%), and iso-C_16:0_ (3.1%). The type strain is LKV-178-WT-2A^T^ (=DSM 108495^T^). It was isolated from faeces of a German Landrace pig in Freising, Germany. Its G+C content of genomic DNA is 49.9 mol%.

Description of *Pseudoramibacter porci sp. nov*.: *Pseudoramibacter* (por’ci. L. gen. n. *porci* of a pig). Anaerobic bacterium that is phylogenetically placed into the family *Eubacteriaceae* (phylum Firmicutes) based on 16S rRNA sequence analysis. *Pseudoramibacter alactolyticus*, the type species of the genus, is the closest phylogenetic neighbour with a sequence identity of 94.3% (1473 bp). The POCP value between the two genomes is 60.8%, which indicates that these species belong to the same genus. The corresponding ANI value <80% and dDDH value of 18.9% confirm that the isolate represents a different species. Cells grow as coccobacilli that can form small chains and stain Gram-positive. Within the genome, six transporters were identified along with ABC transporter systems for phosphate, cobalt, and biotin. Trehalose, starch, and cellulose were identified as potential carbohydrate substrates. Additionally, l-lactate and d-lactate can be converted into pyruvate (EC: 1.1.1.27). Whilst l-aspartate can be converted into fumarate (EC: 6.3.4.4/6.3.4.4, 4.3.2.2/4.3.2.1) and enter the citrate cycle, only the initial conversion into (S)-malate was identified within the genome. Ammonia may be converted into l-glutamine (EC:6.3.1.2), which can then be utilized to form l-glutamate (EC: 1.4.7.1, 1.4.1.13). Production of ornithine, valine, and leucine was identified from glutamate, pyruvate, and 2-oxoisocaproate, respectively. The most dominant cellular fatty acids are C_14:0_ (32%) and iso/anteiso-C_17:1_ (29%). Other fatty acids include C_16:0_ (14.0%), iso-C_19:1_ (5.2%), and C_18:0_ (4.1%). The type strain is RF-744-FAT-4^T^ (=DSM 106894^T^). It was isolated from faeces of an Aachen minipig^83^ in Germany. Its G+C content of genomic DNA is 49.3 mol%.

Description of *Pyramidobacter porci sp. nov*.: *Pyramidobacter porci* (por’ci. L. gen. n. *porci* of a pig). The closest phylogenetic neighbour within family *Synergistaceae* (phylum Synergistetes) is *Pyramidobacter piscolens*, the type species of the genus *Pyramidobacter*, which shares a 16S rRNA gene sequence identity of 95.6%. The dDDH value of 46.7% and ANI value of 92.4% indicate that the isolate represents the second cultured bacterium within this genus. Cells are bacilli with a length of ~0.5 µm. They stain Gram-negative and are strictly anaerobic. Within the genome, a single transporter was identified; however, ABC transporter systems for alkanesulfonate, glycine betaine/proline, biotin, zinc, oligopeptides, d-methionine, *S*-methylcysteine, and phosphate were present. Starch was identified as the sole carbohydrate source. Alternatively, ammonia can be utilized for production of l-glutamine (EC: 6.3.1.2), which can be converted into d-fructose-6-phosphate using d-glucosamine-6-phosphate as an intermediate (EC: 2.6.1.16) and enter glycolysis. Butanoate production was identified to occur via butanoyl-CoA as an intermediate from 3-ocohexanoyl-CoA (EC: 2.3.1.9, 2.8.3.8). After 96 h at 37 °C in modified YCFA medium under anaerobic conditions, the species consumed small amounts of succinate (1.1 ± 0.1 mM) and produced 10.2 ± 0.4 mM acetate and smaller amounts of isovalerate (2.0 ± 0.1 mM), propionate (1.5 ± 0.0 mM), lactate (1.0 ± 0.0 mM), and isobutyrate (0.5 ± 0.0 mM). The major cellular fatty acid is C_18:1 ω9c_ (19%). Other fatty acids include C_14:0 3-OH_/iso-C_16:1_ (9.9%), C_13:0_ (8.7%), C_14:0_ (8.6%), iso-C_13:0_ (6.6%), C_18:0_ (5.9%), C_17:1 ω9c_ (4.7%), C_16:0_ (4.0%), iso-C_15:0 3-OH_ (3.9%), 10Me-C_18:0_ (3.3%), iso-C_15:0_ (3.0%), and C_12:0_ (2.9%). The type strain is SM-530-WT-4B^T^ (=DSM 105193^T^). It was isolated from faeces of a German Landrace pig (minipig breed) in Freising, Germany. Its G+C content of genomic DNA is 59.6 mol%.

Description of *Roseburia porci sp. nov*.: *Roseburia porci* (por’ci. L. gen. n. *porci* of a pig). Phylogenetically (16S rRNA gene-based analysis), this species falls into the family *Lachnospiraceae* (phylum Firmicutes). Its closest neighbours are *Roseburia faecis* and *Roseburia intestinalis* with 95.8% and 95.2% sequence identity, respectively (1432 bp). Any other cultured bacterium with a valid name is characterized by a sequence identity <95%. Of note, the isolate branches close to *Agathobacter* spp., including the well-known butyrate producing species formerly named *Eubacterium rectale*. Despite the urgent need to revisit the taxonomy of *Eubacterium* spp., the placement of *E. rectale* within the genus *Agathobacter* and the status of this genus in general have been disputed, despite being still a valid name^147–150^. Hence, it is sound to propose that the isolate belongs to the genus *Roseburia*. The status of a novel species within this genus is supported by ANI values <80% and dDDH values of 20.6% and 24.0% between its genome and that of its closest relatives *R. faecis* and *R. intestinalis*. Corresponding differences in the G+C content of genomic DNA are 1.3 mol% and 1.0 mol%, which are equal or above the proposed species-delineation threshold of 1 mol%. The strain grows as motile rods that are 1 µm long on average and slightly curved. Growth only occurs under strictly anoxic conditions. A total of 13 transporters along with transporter systems for phosphate, aspartate/glutamate/glutamine, zinc, Na^+^, teichoic acid, bacitracin, d-methionine, biotin, and cystine were identified within the genome. The presence of BceA/B and VraD/E suggests the expression of two bacitracin efflux systems. The DegS/U two-component system for salt stress was also identified. A complete two-component system for bacterial chemotaxis and stimulation of flagella action was identified based on MCP and CheW/A/R/Y/V/B. The presence of flagella is further supported by the identification of the following flagellar assembly proteins: FlhA/B, FliA/C/D/F/G/M/N/S/P, and FlgB/C/E/G/L/K. Cellobiose, starch, cellulose, and d-glucose were identified to be potential carbohydrate substrates. Both Spo0F and Spo0A were identified within the genome, suggesting this species may have the ability to produce spores. Whilst no complete quorum sensing pathway was detected, the presence of both the *luxQ* and *luxS* genes suggests this species may utilize AI-2-based quorum sensing. After 48 h at 37 °C in modified YCFA medium under anaerobic conditions, the species consumed glucose (11.4 ± 0.3 mM) and acetate (5.1 ± 0.7 mM) and produced 12.3 ± 0.2 mM butyrate, 8.8 ± 0.8 mM lactate, and 5.1 ± 0.6 mM formate. The major cellular fatty acids are C_18:0_ (38%), C_13:0 3-OH_/C_15:1 i I/H_, and iso/anteiso-C_17:1_ (18% each). Other fatty acids are C_13:1_ (7.3%) and C_14:0_ (5.2%). The type strain is MUC/MUC-530-WT-4D^T^ (=DSM 107448^T^). It was isolated from faeces of a German Landrace pig (minipig breed) in Freising, Germany. Its G+C content of genomic DNA is 41.7 mol%.

Description of *Scrofimicrobium gen. nov*.: *Scrofimicrobium* (Scro.fi.mi.cro’bi.um. L. fem. n. *scrofa* a breeding sow and the specific epithet of *Sus scrofa* the wild boar; N.L. neut. n. *microbium* a microbe; N.L. neut. n. *Scrofimicrobium* a microbe isolated from a wild boar). The closest phylogenetic neighbours within family *Actinomycetaceae* (phylum Actinobacteria) are *Actinomyces hyovaginalis* and *Actinomyces suimastitidis*, which share 91.8% and 90.6% 16S rRNA gene sequence identity, respectively. POCP and dDDH values against the genome of *S. suimastitidis* are 46.5% and 24.1%, respectively, and 38.2% and 22.6% against *Actinomyces bovis*, the type species of the genus *Actinomyces*. The respective differences in G+C contents of the genomic DNA are 2.9% and 10.6%. Of note, no genome is available for *S. hyovaginalis*. All values aforementioned clearly speak in favour of creating a new genus to accommodate strain WB03_NA08, for which the name *Scrofimicrobium* is proposed. The type species is *Scrofimicrobium canadense*.

Description of *Scrofimicrobium canadense sp. nov*.: *Scrofimicrobium canadense* (ca.na.den’se. N.L. neut. adj. *canadense* Canadian, referring to the country where the type strain was isolated). Cells are rod-shaped that strain Gram-positive and have a length of 1-2 µm. The species is a facultative anaerobe. Transporter systems for arabino-oligosaccharides, monosaccharides, zinc, glutathione, d-methionine, cysteine, phosphate, and glutamate were identified within the genome. The SecYEG system for protein export was also detected. A two-component system for phosphate limitation was present, which consisted of SenX3, RegX3, PhoA, and PstS. A complete TCA cycle was identified with singular input from acetyl-CoA. Acetyl-CoA can be produced from both acetate or glucose using a modified glycolysis pathway. Due to the lack of enzymes for the production or utilization of d-fructose-1,6-bisphosphate, glucose-6-phosphate was identified to be converted into glucono-1,5-lactone-6-phosphate (EC: 1.1.1.49), which is converted into gluconate-6-phosphate (EC: 3.1.1.31). This then becomes ribulose-5-phosphate (EC: 1.1.1.44), which converts to d-xyulose-5-phosphate (EC: 5.1.3.1). d-xyulose-5-phophate is then converted into d-glyceraldehyde-3-phosphate and re-enters glycolysis. After 48 h at 37 °C in modified YCFA medium under anaerobic conditions, the species consumed glucose (8.0 ± 1.6 mM) and produced 9.6 ± 2.0 mM succinate, 3.8 ± 0.5 mM acetate, 3.0 ± 0.3 mM formate, and 1.6 ± 1.0 mM lactate. Major cellular fatty acids are C_16:0_ (32%), C_14:0_ (32%), and C_18:1 ω9c_ (10%). Other fatty acids include C_12:0_ (6.4%), C_18:1 ω7c_ (6.2%), C_20:1 ω9c_ (4.7%), and C_18:0_ (4.2%). The type strain is WB03_NA08^T^ (=DSM 105338^T^). It was isolated from faeces of a farmed wild boar in Moserville, Ontario, Canada. Its G+C content of genomic DNA is 53.6 mol%.

Description of *Selenomonas montiformis sp. nov*.: *Selenomonas montiformis* (mon.ti.for’mis. L. masc. n. *mons* mountain; L. fem. n. *forma* shape; N.L. fem. adj. *montiformis* forming mountain-shaped colonies on agar). Its closest relative based on 16S rRNA gene sequence identity (96.0%) is *Selenomonas bovis* within family *Selenomonadaceae* (phylum Firmicutes). The dDDH value of 23.8% and ANI value <80% between the two genomes indicate that the two bacteria represent separate species. Cells are motile, slightly curved rods (1–2 µm) that form colonies resembling mini-mountains on WCA agar under anaerobic conditions. Eight transporters were identified within the genome along with transport systems for phosphate, alkanesulfonate, d-methionine, ribose/AI-2/d-zylose, cobalt, and biotin. The DegS/U two-component system for salt stress was also identified. A complete two-component system for bacterial chemotaxis and stimulation of flagella action was identified based on the detection of genes ecnoding MCP and CheW/A/R/Y/V/B. The presence of flagella is further supported by the identification of the following flagellar assembly proteins: FlhA/B, FliA/C/D/F/G/M/N/S/P, FlgB/C/E/G/L/K, and MotB. This isolate was predicted to utilize a wide range of carbohydrate substrates, including sucrose, cellobiose, cellulose, starch, trehalose, maltose, arbutin, salicin, and d-glucose. Biosynthesis of glycogen via GlgA/C/P may suggest a role for this isolate in biofilm production which is supported by the presence of CsgD, termed the ‘master regulator’ for biofilm production. Indole was identified to be produced from shikimate using chorismate as an intermediate (EC: 2.7.1.71, 2.5.1.19, 4.2.3.5, 4.1.3.27, 2.4.2.18, 5.3.1.24, 4.1.1.48, 4.2.1.20). After 48 h at 37 °C in modified YCFA medium under anaerobic conditions, the species consumed glucose (10.4 ± 0.1 mM) and trace amounts of succinate (0.6 ± 0.3 mM) and valerate (0.4 ± 0.2 mM) and produced 22.9 ± 0.8 mM lactate, 8.9 ± 0.9 mM propionate, 5.7 ± 0.5 mM acetate, and smaller amounts of isobutyrate (0.6 ± 0.1 mM). The major cellular fatty acids are C_13:0 3OH_/C_15:1 i I/H_ (20%), C_15:0_ (13%), C_15:1 ω8c_ (13%), and C_16:1 ω9c_ (10%). Lower amounts of C_17:1 ω8c_ (9.8%), C_11:0_ (9.3%), C_13:0_ (5.8%), and iso-C_18:1_ (4.7%) were detected. The type strain is WCA-380-WT-3B3^T^ (=DSM 106892^T^). It was isolated from faeces of a German Landrace pig in Freising, Germany. Its G+C content of genomic DNA is 52.6 mol%.

Description of *Sharpea porci sp. nov*.: *Sharpea porci* (por’ci. L. gen. n. *porci* of a pig). The closest phylogenetic neighbour is *Sharpea azabuensis* within the family *Erysipelotrichaceae* (phylum Firmicutes), which shares 98.4% 16S rRNA gene sequence identity. The dDDH and ANI values of 36.4% and 88.9% between the two genomes confirm that the isolate represents a novel species within the genus *Sharpea*. The species grows as thick rods with a length of 2–8 µm that often forms chains. A single transporter was identified within the genome along with transporter systems for phosphate, cysteine, teichoic acid, d-methionine, oligopeptides, biotin, and zinc. Cellubiose, starch, maltose, trehalose, sucrose, arbutin, salicin, and d-glucose were predicted to be potential carbohydrate substrates. Indole was identified to be produced from shikimate using chorismate as an intermediate (EC: 2.7.1.71, 2.5.1.19, 4.2.3.5, 4.1.3.27, 2.4.2.18, 5.3.1.24, 4.1.1.48, 4.2.1.20). l-cysteine can also be produced internally using sulfide and acetyl-l-serine (EC:2.5.1.47). The major cellular fatty acids are C_16:0_ (24%), C_18:0_ (13%), and C_12:0_ (12%). Lower amounts of C_18:1 ω9c_ (6.6%), C_19:1 ISO I_ (5.7%), C_14:0_ (4.8%), C_16:1 ω7c_/iso-C_15:1 2OH_ (4.7%), iso I/anteiso B-C_17:1_ (4.7%), C_10:0_ (3.8%), C_17:0 ISO 3OH_ (3.7%), C_17:1 ω9c_ (3.1%), C_18:1 ω7c_ (2.9%), C_18:2 ω6,9c_/anteiso-C_18:0_ (2.8%), and C_14:1 ω5c_ (2.1%) were detected. The type strain is CA-Schmier-601-WT-3^T^ (=DSM 108165^T^). It was isolated from faeces of a German Landrace pig (minipig breed) in Freising, Germany. Its G+C content of genomic DNA is 30.6 mol%.

Description of *Sodaliphilus gen. nov*.: *Sodaliphilus* (So.da.li’phi.lus. L. masc. or fem. n. *sodalis* companion; N.L. masc. adj. *philus* (from Gr. masc. adj. philos) *loving*; N.L. masc. n. *Sodaliphilus* an organism that loves a companion). The isolate is phylogenetically placed into the family *Muribaculaceae* (phylum Bacteroidetes). Based on 16S rRNA gene sequence identity, *Muribaculum intestinale* is the closest phylogenetic neighbour (86.4%). Comparison with members of the neighbouring family *Porphyromonadaceae* (*Barnesiella* and *Coprobacter* spp.) revealed 16S rRNA sequence identity values <85.7%. The difference in the G+C content of genomic DNA between the isolate and *M. intestinale* is 4.31%. The POCP value between the two genomes is 42%, which clearly demonstrates a separate genus status. Compared to members of the three genera aforementioned, *Coprobacter secundus* shows the highest dDDH value with 42.3% (ANI value < 75%) and *Barnesiella viscericola* the highest ANI value with 76.6% (dDDH value = 17.0%). These values are clearly below thresholds justifying separate taxa. The type species is *Sodaliphilus pleomorphus*.

Description of *Sodaliphilus pleomorphus sp. nov*.: *Sodaliphilus pleomorphus* (ple.o.mor’phus. Gr. adv. *pleon* more; Gr. fem. n. *morphe* form, shape; N.L. masc. adj. *pleomorphus* having multiple shapes). Cells grow slowly and only under strictly anaerobic conditions and on solid medium. After 1 week of incubation, cells are irregular rods that can be very long (up to 50 µm). Longer incubation times result in shortening and thickening of cells. Growth of the species was observed to be boosted when co-incubated on plate with other isolates, including *Tissierella pigra* sp. nov., *Hornefia butyriciproducens* gen. nov., sp. nov., and *Anaerobutyricum soehngenii*. Three transporters were identified within the genome along with transport systems for phosphate and lipoproteins. A two-component system (PhoA/B/R) was also identified, which responds to limited phosphate by increasing its assimilation. Starch, amylose, dextrin, and fructan may be utilized by this strain. The glycolysis pathway lacked multiple proteins, suggesting an inability to directly convert phosphoenolpyruvate to pyruvate, instead phosphenolpyruvate can be utilized for production of chorismate (EC: 2.5.1.54, 4.2.3.4, 4.2.1.10, 1.1.1.25, 2.7.1.71, 2.5.1.19, 4.2.3.5), indole, and l-tryptophan (EC: 4.1.3.27, 2.4.2.18, 5.3.1.24, 4.1.1.48, 4.2.1.20). Lipid IV (A) can be produced from lipid X (EC: 2.4.1.182, 2.7.1.130), a hallmark functional feature of *Muribaculaceae*. Lipid IV (A) can also be converted into lauroyl-KD02-lipid IV (A) (EC: 2.4.99.12, 2.4.99.13, 2.3.1.241). Based on metagenomic prediction, this species is proposed to be a key functional member of pig microbiomes. The biomass collected from three agar plates produced 1.6 ± 0.7 mM formate and traces of isobutyrate (0.5 ± 0.1 mM) after 48 h at 37 °C in modified YCFA medium under anaerobic conditions. The type strain is Oil-RF-744-WCA-WT-10^T^ (=DSM 108610^T^). It was isolated from faeces of an Aachen minipig^83^ in Germany. Its G+C content of genomic DNA is 54.4 mol%.

Description of *Stecheria gen. nov*.: *Stecheria* (Ste.che’ri.a. N.L. fem. n. *Stecheria* named in honour of Prof. Bärbel Stecher from the LMU Munich, Germany, for her contribution to the field of enteric infection by *Salmonella* and interactions of enteric pathogens with commensal microbes). Based on 16S rRNA gene phylogeny, the genus is placed into the family *Erysipelotrichaceae* (phylum Firmicutes). It shares 90.4% sequence identity with both *Solobacterium moorei* and *Bulleidia extructa* (1487 bp). The POCP value between the genome of the isolate and the respective genome of these phylogenetic neighbours is 40.0% and 37.1%. This confirms a separate genus status, for which the name *Stecheria* is proposed. The type species is *Stecheria intestinalis*.

Description of *Stecheria intestinalis sp. nov*.: *Stecheria intestinalis* (in.tes.ti.na’lis. N.L. fem. adj. *intestinalis* pertaining to the gut). The species grows as pear-shaped, coccoidal cells that may form pairs or small chains of 2–5 µm in length. They stain Gram-positive and grow only under strictly anaerobic conditions. One single transporter was identified within the genome along with ABC transporter systems for phosphate, teichoic acid, d-methionine, oligopeptides, and methyl-galactoside. Serine protease activity was identified to be modulated by the CssS/R-HtrA two-component system for removal of misfolded proteins. Arbutin, salicin, d-glucose, sucrose, trehalose, maltose, cellobiose, and starch were identified as potential carbohydrate substrates. Propionic acid production was identified to occur from acryloyl-CoA (EC: 1.3.8.1, 2.3.1.8/2.3.1.222, 2.7.2.1) and 2-ocobutanoate (EC: 2.3.1.54, 2.3.1.8, 2.7.2.1). The species is considered to deconjugate primary bile acids as determined in vitro by cleavage of taurine residues on WCA agar medium containing TDCA. The main cellular fatty acid is C_18:1 ω9c_ (70%). C_18:1 ω7c_ (21.8%) and C_18:0_ (5.4%) were detected in lower amounts. The type strain is Oil+RF-744-GAM-WT-6^T^ (=DSM 109718^T^). It was isolated from faeces of an Aachen minipig^83^ in Germany. Its G+C content of genomic DNA is 49.4 mol%.

Description of *Suipraoptans gen. nov*.: *Suipraoptans* (Su.i.prae.op’tans. L. masc. n. *sus* a pig; L. pres. part. *praeoptans* preferring; N.L. masc. n. *Suipraeoptans* an organism preferring pigs, referring to its higher occurrence in the intestine of pigs vs. humans and mice). Based on 16S rRNA gene sequences, the nearest phylogenetic relatives of this strictly anaerobic bacterium are *Ruminococcus lactaris*, *Faecalicatena fissicatena*, and *Dorea longicatena* (<93% sequence identity). The corresponding POCP values are 41.0%, 44.9%, and 52.3%. *Dorea formicigenerans*, the type species of this genus, shares 91.4% 16S rRNA gene sequence identity and a POCP value of 48.5% with the isolate, confirming its status as a distinct genus, for which the name *Suipraeoptans* is proposed. The type species is *Suipraeoptans intestinalis*.

Description of *Suipraoptans intestinalis sp. nov*.: *Suipraoptans intestinalis* (in.tes.ti.na’lis. N.L. masc. adj. *intestinalis* pertaining to the gut). The species forms short (0.5 µm) straight rods that grow as single cells or in pairs and stain Gram-positive. Within the genome, 13 transporters were identified along with transporter systems for phosphate, *S*-methylcysteine, bacitracin, d-methionine, zinc, cobalt, biotin, and d-xylose. Both starch and trehalose were identified as potential substrates. l-tryptophane production from hydroxypyruvate may occur via serine (EC: 1.1.1.29, 2.7.1.165, 5.4.2.12, 1.1.1.95, 2.6.1.52, 3.1.3.3, 4.2.1.20). l-serine could also be converted into *O*-acetyl-l-serine (EC: 2.3.1.30) and combined with sulfide to form l-cysteine (EC: 2.5.1.47). Anaerobic conversion of Mg-protoporphyrin IX 13-monomethyl ester to divinyl-protochlorophyllide was identified; however, no downstream or upstream production pathways were present. Both cobalamin biosynthesis from cobinamide (CobA/P/Q, CbiB) and production of folic acid from 7,8-dihydrofolate (EC: 1.5.1.3) were identified. After 48 h at 37 °C in modified YCFA medium under anaerobic conditions, the species consumed glucose (10.2 ± 0.4 mM) and produced 10.9 ± 1.6 mM acetate and traces of formate (0.7 ± 0.0 mM). The major fatty acid is C_16:0_ (48%). Other fatty acids are C_18:0_ (11.8%), iso-C_17:1_/anteiso-C_17:1_ (10.5%), C_14:0_ (4.8%), and iso-C_19:1_ (4.2%). The type strain is 68-1-5^T^ (=DSM 104945^T^). It was isolated from the ileal content of a pig in Ames, Iowa, USA. Its G+C content of genomic DNA is 47.7 mol%.

Description of *Tissierella pigra sp. nov*.: (pi’gra. L. fem. adj. *pigra* lazy, pertaining to the very slow growth of the type strain in liquid medium compared with good growth on agar). The closest phylogenetic relatives within family *Tissierellaceae* (phylum Firmicutes) are *Tissierella praeacuta* (97.8% 16S rRNA gene sequence identity), the type species of the genus *Tissierella*, and *Tissierella carlieri* (96.3%). The dDDH/ANI values of 22.4%/80.0% and 24.6%/81.1%, respectively, confirm the status of the isolate as a novel species. Cells are rod-shaped with a size of 1.5–2 µm but they can also form long filaments of up to 20 µm. They grow under strictly anaerobic conditions. Only two transporters were identified within the genome with additional transporter systems for phosphate, alkanesulfonate, *S*-methylcysteine, d-methionine, oligopeptides, glutathione, zinc, cobalt, and biotin. The SecYEG system for protein export was also present. A two-component system for SCFA metabolism based on the detection of acetoacetate was identified (AtoA/B/C/D/S). Another complete two-component system for bacterial chemotaxis and stimulation of flagella action was identified based on MCP and CheW/A/R/Y/V/B. The presence of flagella is further supported by the identification of the assembly proteins FlhA/B, FlgB/C/D/E/G/K, FliA/C/D/E/F/G/M/N/S/P, and MotB, and was confirmed by electron microscopy (Supplementary Fig. 7). Propanoate may be produced from acryloyl-CoA via conversion to propanoyl-CoA (EC: 1.3.8.1), propanoyl phosphate (EC: 2.3.1.8, 2.3.1.222) and then propanoate (EC: 2.7.2.1). Cellobiose was the only carbohydrate source identified to be used by this isolate (EC: 2.7.1.205). The presence of genes for the utilization of multiple sulfur sources including sulfate (*cysPUWA*), taurine (*tauACB*), and alkanesulfonate (*ssuACB*) may suggest a sulfur-centric metabolic system. After 48 h at 37 °C in modified YCFA medium under anaerobic conditions, the species did not consume glucose and produced 16.7 ± 0.6 mM acetate, 10.5 ± 1.0 mM isovalerate, 4.6 ± 0.4 mM butyrate, and 3.0 ± 0.6 mM isobutyrate. The major cellular fatty acid is iso-C_15:0_ (40%). Other fatty acids include C_14:0_ (8.5%), C_16:0_ (7.1%), iso-C_17:1 ω9c_ (6.3%), iso-C_13:0_ (4.7%), C_12:0_ (4.1%), iso-C_15:1_ (4.0%), C_18:0_ (3.7%), anteiso-C_15:0_ (3.6%), and C_18:1 ω9c_ (3.3%). The type strain is WCA3-693-APC-4α^T^ (= DSM 105185^T^). It was isolated from faeces of an APC^1311/+^ pig in Freising, Germany. Its G+C content of genomic DNA is 36.9 mol%.

Description of *Velocimicrobium gen. nov*.: *Velocimicrobium* (Ve.lo.ci.mi.cro’bi.um. L. masc. adj. *velox* swift, rapid; N.L. neut. n. *microbium* a microbe; N.L. neut. n. *Velocimicrobium* a fast-growing and motile microbe). Strictly anaerobic bacterium that is phylogenetically placed into the family *Lachnospiraceae* (phylum Firmicutes). Based on 16S rRNA sequence identity, the closest phylogenetic neighbour is *Acetivibrio ethanolgignens*, which shares 94.2% sequence identity (1484 bp). The isolate is also distantly related to *Anaerocolumna cellulosilytica*, the types species of this genus (91.5% sequence identity). Whilst no genome is available for *A. cellulosilytica*, the POCP value between the genome of our isolate and that of *A. ethanolgignens* is 49.0%, which highlights the need to create a new genus. The corresponding ANI and dDDH values of 78.5% and 21.5% confirm that the isolate represents a different taxon. The type species is *Velocimicrobium porci*.

Description of *Velocimicrobium porci sp. nov*.: *Velocimicrobium porci* (por’ci. L. gen. n. *porci* of a pig). The species grows as straight, thick rods of 2–3 µm in length. They stain Gram-positive. A single transporter was identified within the genome along with ABC transporter systems for phosphate, cystine, teichoic acid, bacitracin, d-methionine, zinc, cobalt, biotin, and methyl-galactoside. Starch, maltose, trehalose, arbutin, salicin, and d-glucose are potential carbohydrate substrates. The motility of this species as observed under light microscopy is confirmed by the presence of the flagella-related genes *fliA/C/D/F/G/M/N/P/S*, *flhA/B*, *flgB/C/D/E/G/K/L*, and *motB*. Additionally, this species may be a biofilm producer as the *glgA/C/P* glycogen biosynthesis genes and the CsgD biofilm regulator were detected. The presence of both the ArcB and OxyR biofilm regulator genes suggest oxidative stress may be an inducer for biofilm production. After 48 h at 37 °C in modified YCFA medium under anaerobic conditions, the species consumed glucose (11.1 ± 0.6 mM) and produced 12.6 ± 2.7 mM acetate, 10.2 ± 1.3 mM formate, and small amounts of propionate (1.1 ± 0.9 mM) and isobutyrate (0.9 ± 0.2 mM). The predominant cellular fatty acid is C_18:1 ω9c_ (67%). Other fatty acids are C_18:1 ω7c_ (11.9%), C_18:0_ (4.8%), C_20:0_ (4.3%), iso-C_19:0_ (4.1%), and C_16:0_ (3.8%). The type strain is WCA-693-APC-MOT-I^T^ (=DSM 107250^T^). It was isolated from faeces of an APC^1311/+^ pig in Freising, Germany. Its G+C content of genomic DNA is 34.9 mol%.

Description of *Victivallis lenta sp. nov*.: *Victivallis lenta* (L. fem. adj. *lenta* slow). The 16S rRNA gene sequence of this species (1471 bp) shares 95.1% identity with the sequence of *Victivallis vadensis*, the type species of the genus *Victivallis* within family *Victivallaceae* (phylum Lentisphaerae). The ANI and dDDH values are 80.6% and 24.1%, respectively, which support the creation of a novel species to accommodate the isolate. Of note, one strain (CCUG 44730) without a valid name previously isolated by others from the blood of an 80-year-old woman share ANI and dDDH values of 98.8% and 93.3%, respectively, showing that it belongs to the same species as our own isolate. Within the genome, two transporters were identified along with ABC transporter systems for phosphate, glycine betaine, proline, teichoic acid, ribose, AI-2, and d-xylose. Starch, cellulose, and frunctan were identified as potential carbohydrate substrates. Coenzyme-A biosynthesis was identified from pantothenol (EC: 2.7.1.33, 6.3.2.5, 4.1.1.36, 2.7.7.3, 3.6.1.9, 2.7.1.24) and l-aspartate (EC: 4.1.1.11, 6.3.2.1) or l-valine (EC: 2.6.1.42, 2.1.2.11, 1.1.1.169, 6.3.2.1). Ammonia can be converted into l-glutamine (EC: 6.3.1.2), which in turn can be utilized to form l-glutamate (EC: 1.4.1.13). l-aspartate may be converted into fumarate via either l-arginino-succinate (EC: 6.3.4.5, 4.3.2.1) or adenylo-succinate (EC: 6.3.4.4, 4.3.2.2). The HighA-1 antitoxin was identified, which counteracts the effect of the HigB-1 toxin. After 48 h at 37 °C in modified YCFA medium under anaerobic conditions, the species consumed glucose (10.2 ± 0.2 mM) and produced acetate (4.5 ± 0.9 mM), lactate (1.3 ± 0.1 mM), and low amounts of formate (0.5 ± 0.1 mM). The main cellular fatty acids are C_18:3 ω6c_ (30%), iso-C_16:0_ (22%), and anteiso-C_15:0_ (18%). Other fatty acids are C_18:0_ (6.6%), C_16:0_ (5.6%), iso-C_18:0_ (4.7%), iso-C_14:0_ (3.7%), and anteiso-C_17:0_ (3.5%).The type strain is BBE-744-WT-12^T^ (=DSM 107290^T^). It was isolated from faeces of an Aachen minipig^83^ in Germany. Its G+C content of genomic DNA is 59.3 mol%.

Description of *Waltera gen. nov*.: *Waltera* (Wal’te.ra, N.L. fem. n. *Waltera* named in honour of Prof. Jens Walter from APC Microbiome Ireland, School of Microbiology and Department of Medicine, University College Cork, National University of Ireland, for his contribution to the field of gut microbiology). This isolate is phylogenetically placed into the family *Lachnospiraceae* (phylum Firmicutes). It is a strictly anaerobic bacterium that is distantly related to *Kineothrix alysoides*, the type species of the genus *Kineothrix*, with which it shares 92.6% 16S rRNA gene sequence identity (1470 bp). The difference in the G+C content of genomic DNA between the two genomes is 3.6%. The ANI value <80% as well as POCP and dDDH values of 43.8% and 18.7%, respectively, clearly demonstrate that the isolate represents a new taxon, for which the creation of a novel genus is required. The type species is *Waltera intestinalis*.

Description of *Waltera intestinalis sp. nov*.: *Waltera intestinalis* (in.tes.ti.na’lis. N.L. fem. adj. *intestinalis* pertaining to the gut). Cells are motile, stain Gram-negative, and grow as wavy rods of 2–4 µm in length. Within the genome, four transporters were identified along with ABC transporter systems for phosphate, alkanesulfonate, teichoic acid, Na^+^, d-methionine, oligopeptides, methyl-galactoside, biotin and the Peb1A/B/C system for aspartate, glutamate, and glutamine. The presence of both the PhoB/R/P and SenX3-RegX3 two-component systems for detecting phosphate limitation leading to phosphate assimilation indicates this species is sensitive to environmental phosphate levels. The MCP, CheA/B/R/Y/V/W two-component system leading to flagella assembly was also detected within the genome along with the MotB, FliA/C/D/F/G//M/N/S/P, FlgB/C/D/E/G/K/L, and FlhA/B flagella proteins. Cellulose, starch, trehalose, maltose, arbutin, salicin, and d-glucose were all identified as potential carbohydrate substrates. Based on the analysis of a large catalogue of metagenome-reconstructed genomes, the species is proposed to be a prevalent member of the intestinal microbiome also in human. The major cellular fatty acid is C_10:0_ (33%). Lower proportions of the following fatty acids were detected: C_16:0_ (14.4%), C_18:0_ (11.0%), alde-C_12:0_ (8.7%), C_12:0_ (7.4%), and C_18:1 ω9c_ (5.9%). The type strain is WCA3-601-WT-6H^T^ (=DSM 108985^T^). It was isolated from faeces of a German Landrace pig (minipig breed) in Freising, Germany. Its G+C content of genomic DNA is 46.3 mol%.

### Statistics and reproducibility

Bacterial occurrences (median relative abundances) presented in Fig. 4c were tested statistically using a Wilcoxon Rank Sum test with Benjamini–Hochberg correction (adj. *p*-value <0.05). Transcriptomic analysis as presented in Fig. 6e, f was performed in R using the packages edgeR and limma, including Bonferroni correction of *p*-values. Data in Supplementary Fig. 13 were tested by ANOVA on Rank in Rhea (https://lagkouvardos.github.io/Rhea). Cell morphological characteristics detected via light and fluorescence microscopy could be repeatedly (>3 replica) observed in independent biological replicates (Figs. 2a, b, 6b and Supplementary Figs. 4a–v and 10). Cell architectures of *Bullifex proci*, *Pseudoramibacter porci*, *Stecheria intestinalis*, and *Tissierella pigra* analysed by TEM or SEM (Fig. 2b and Supplementary Figs. 5a, b, 6a, b, 7a, b, 8a, b) were identical between different preparation methods as stated in the ‘Methods’ sections ‘Bacterial cultures for further microscopy analyses’ and ‘Transmission electron microscopy’. In case of *Bullifex porci*, two independent laboratories with varying procedures demonstrated the same structural pattern using transmission electron microscopy.

### Reporting summary

Further information on research design is available in the Nature Research Reporting Summary linked to this article.

## Supplementary information

Supplementary Information

Peer Review File

Description of Additional Supplementary Files

Supplementary Data 1

Supplementary Data 2

Supplementary Data 3

Reporting Summary

## Data Availability

All 16S rRNA genes sequences and genomes generated in the present study are available via GenBank using the individual accession numbers provided in Supplementary Data 1. Metagenome-assembled genomes are publicly available at https://github.com/strowig-lab/PIBAC, referenced under 10.5281/zenodo.4075024. The 16S rRNA gene amplicon data from pig faeces, the transcriptomic data from in vitro cultures, and all other data from this study are acccessible in NCBI via project PRJNA561470.

## Code availability

The codes for metagenomic analyses are publicly available at https://github.com/strowig-lab/PIBAC, referenced under 10.5281/zenodo.4075065.

## References

[1] Engber D (2018). What models eat. Nat. Med..

[2] Gehrig JL (2019). Effects of microbiota-directed foods in gnotobiotic animals and undernourished children. Science.

[3] Gonzalez LM, Moeser AJ, Blikslager AT (2015). Porcine models of digestive disease: the future of large animal translational research. Transl. Res..

[4] Flisikowska T (2012). A porcine model of familial adenomatous polyposis. Gastroenterology.

[5] Blutke A (2017). The Munich MIDY Pig Biobank - a unique resource for studying organ crosstalk in diabetes. Mol. Metab..

[6] Arthur JC (2014). Microbial genomic analysis reveals the essential role of inflammation in bacteria-induced colorectal cancer. Nat. Commun..

[7] Coleman, O. I. et al. Activated ATF6 induces intestinal dysbiosis and innate immune response to promote colorectal tumorigenesis. *Gastroenterology* **155**, 1539–1552.e12 (2018). 10.1053/j.gastro.2018.07.028 30063920

[8] Zitvogel L, Ma Y, Raoult D, Kroemer G, Gajewski TF (2018). The microbiome in cancer immunotherapy: diagnostic tools and therapeutic strategies. Science.

[9] Just S (2018). The gut microbiota drives the impact of bile acids and fat source in diet on mouse metabolism. Microbiome.

[10] Khan MT, Nieuwdorp M, Backhed F (2014). Microbial modulation of insulin sensitivity. Cell Metab..

[11] Plovier H (2017). A purified membrane protein from Akkermansia muciniphila or the pasteurized bacterium improves metabolism in obese and diabetic mice. Nat. Med..

[12] Gaskins HR, Collier CT, Anderson DB (2002). Antibiotics as growth promotants: mode of action. Anim. Biotechnol..

[13] Ramayo-Caldas Y (2016). Phylogenetic network analysis applied to pig gut microbiota identifies an ecosystem structure linked with growth traits. ISME J..

[14] Brugiroux S (2016). Genome-guided design of a defined mouse microbiota that confers colonization resistance against Salmonella enterica serovar Typhimurium. Nat. Microbiol..

[15] Kumar A (2018). Impact of nutrition and rotavirus infection on the infant gut microbiota in a humanized pig model. BMC Gastroenterol..

[16] Lagkouvardos I (2019). Sequence and cultivation study of Muribaculaceae reveals novel species, host preference, and functional potential of this yet undescribed family. Microbiome.

[17] Parks DH (2017). Recovery of nearly 8,000 metagenome-assembled genomes substantially expands the tree of life. Nat. Microbiol..

[18] Xiao, L. et al. A reference gene catalogue of the pig gut microbiome. *Nat. Microbiol.* **1**, 16161 (2016). 10.1038/nmicrobiol.2016.161 27643971

[19] Lagkouvardos I (2016). The mouse intestinal bacterial collection (miBC) provides host-specific insight into cultured diversity and functional potential of the gut microbiota. Nat. Microbiol..

[20] Xiao L (2015). A catalog of the mouse gut metagenome. Nat. Biotechnol..

[21] Browne HP (2016). Culturing of ‘unculturable’ human microbiota reveals novel taxa and extensive sporulation. Nature.

[22] Lagier JC (2018). Culturing the human microbiota and culturomics. Nat. Rev. Microbiol..

[23] Aarestrup F (2012). Sustainable farming: get pigs off antibiotics. Nature.

[24] Rhouma M, Beaudry F, Theriault W, Letellier A (2016). Colistin in pig production: chemistry, mechanism of antibacterial action, microbial resistance emergence, and one health perspectives. Front. Microbiol..

[25] Reimer LC (2019). BacDive in 2019: bacterial phenotypic data for High-throughput biodiversity analysis. Nucleic Acids Res..

[26] Zheng, J. et al. A taxonomic note on the genus Lactobacillus: description of 23 novel genera, emended description of the genus Lactobacillus Beijerinck 1901, and union of Lactobacillaceae and Leuconostocaceae. *Int. J. Syst. Evol. Microbiol.* **70**, 2782–2858 (2020). 10.1099/ijsem.0.004107 32293557

[27] Abt B (2012). Complete genome sequence of the termite hindgut bacterium Spirochaeta coccoides type strain (SPN1(T)), reclassification in the genus Sphaerochaeta as Sphaerochaeta coccoides comb. nov. and emendations of the family Spirochaetaceae and the genus Sphaerochaeta. Stand. Genomic Sci..

[28] Ritalahti KM (2012). Sphaerochaeta globosa gen. nov., sp. nov. and Sphaerochaeta pleomorpha sp. nov., free-living, spherical spirochaetes. Int. J. Syst. Evol. Microbiol..

[29] Troshina O (2015). Sphaerochaeta associata sp. nov., a spherical spirochaete isolated from cultures of Methanosarcina mazei JL01. Int. J. Syst. Evol. Microbiol..

[30] Du S, Lutkenhaus J (2017). Assembly and activation of the Escherichia coli divisome. Mol. Microbiol..

[31] Wagstaff J, Löwe J (2018). Prokaryotic cytoskeletons: protein filaments organizing small cells. Nat. Rev. Microbiol..

[32] Wiegand S (2020). Cultivation and functional characterization of 79 planctomycetes uncovers their unique biology. Nat. Microbiol..

[33] Wagner JK, Galvani CD, Brun YV (2005). Caulobacter crescentus requires RodA and MreB for stalk synthesis and prevention of ectopic pole formation. J. Bacteriol..

[34] Divakaruni AV, Baida C, White CL, Gober JW (2007). The cell shape proteins MreB and MreC control cell morphogenesis by positioning cell wall synthetic complexes. Mol. Microbiol..

[35] Vats P, Shih YL, Rothfield L (2009). Assembly of the MreB-associated cytoskeletal ring of Escherichia coli. Mol. Microbiol..

[36] Rutledge PJ, Challis GL (2015). Discovery of microbial natural products by activation of silent biosynthetic gene clusters. Nat. Rev. Microbiol..

[37] Haft DH, Basu MK (2011). Biological systems discovery in silico: radical S-adenosylmethionine protein families and their target peptides for posttranslational modification. J. Bacteriol..

[38] Babasaki K, Takao T, Shimonishi Y, Kurahashi K, Subtilosin A (1985). a new antibiotic peptide produced by Bacillus subtilis 168: isolation, structural analysis, and biogenesis. J. Biochem..

[39] Lee H, Churey JJ, Worobo RW (2009). Biosynthesis and transcriptional analysis of thurincin H, a tandem repeated bacteriocin genetic locus, produced by Bacillus thuringiensis SF361. FEMS Microbiol. Lett..

[40] Gonzalez-Pastor JE, Hobbs EC, Losick R (2003). Cannibalism by sporulating bacteria. Science.

[41] Rea MC (2010). Thuricin CD, a posttranslationally modified bacteriocin with a narrow spectrum of activity against Clostridium difficile. Proc. Natl Acad. Sci. USA.

[42] Balty C (2019). Ruminococcin C, an anti-clostridial sactipeptide produced by a prominent member of the human microbiota Ruminococcus gnavus. J. Biol. Chem..

[43] Faijes M, Castejon-Vilatersana M, Val-Cid C, Planas A (2019). Enzymatic and cell factory approaches to the production of human milk oligosaccharides. Biotechnol. Adv..

[44] Seshadri R (2018). Cultivation and sequencing of rumen microbiome members from the Hungate1000 Collection. Nat. Biotechnol..

[45] Petschacher B, Nidetzky B (2016). Biotechnological production of fucosylated human milk oligosaccharides: Prokaryotic fucosyltransferases and their use in biocatalytic cascades or whole cell conversion systems. J. Biotechnol..

[46] Jost F, de Vries T, Knegtel RM, Macher BA (2005). Mutation of amino acids in the alpha 1,3-fucosyltransferase motif affects enzyme activity and Km for donor and acceptor substrates. Glycobiology.

[47] Lagkouvardos I (2016). IMNGS: a comprehensive open resource of processed 16S rRNA microbial profiles for ecology and diversity studies. Sci. Rep..

[48] Yilmaz P (2014). The SILVA and “All-species Living Tree Project (LTP)” taxonomic frameworks. Nucleic Acids Res..

[49] Bowers RM (2017). Minimum information about a single amplified genome (MISAG) and a metagenome-assembled genome (MIMAG) of bacteria and archaea. Nat. Biotechnol..

[50] Lesker TR (2020). An integrated metagenome catalog reveals new insights into the murine gut microbiome. Cell Rep..

[51] Pasolli E (2019). Extensive unexplored human microbiome diversity revealed by over 150,000 genomes from metagenomes spanning age, geography, and lifestyle. Cell.

[52] Segata N, Börnigen D, Morgan XC, Huttenhower C (2013). PhyloPhlAn is a new method for improved phylogenetic and taxonomic placement of microbes. Nat. Commun..

[53] Parks DH (2018). A standardized bacterial taxonomy based on genome phylogeny substantially revises the tree of life. Nat. Biotechnol..

[54] Lagkouvardos I, Overmann J, Clavel T (2017). Cultured microbes represent a substantial fraction of the human and mouse gut microbiota. Gut Microbes.

[55] Steen, A. D. et al. High proportions of bacteria and archaea across most biomes remain uncultured. *ISME J* (2019). 10.1038/s41396-019-0484-y PMC6863901 31388130

[56] Becker N, Kunath J, Loh G, Blaut M (2011). Human intestinal microbiota: characterization of a simplified and stable gnotobiotic rat model. Gut Microbes.

[57] Schaedler RW, Dubs R, Costello R (1965). Association of germfree mice with bacteria isolated from normal mice. J. Exp. Med..

[58] El-Gebali S (2019). The Pfam protein families database in 2019. Nucleic Acids Res..

[59] Yang H (2019). Antibiotic application and resistance in swine production in China: current situation and future perspectives. Front. Vet. Sci..

[60] Liu YY (2016). Emergence of plasmid-mediated colistin resistance mechanism MCR-1 in animals and human beings in China: a microbiological and molecular biological study. Lancet Infect. Dis..

[61] Hang S (2019). Bile acid metabolites control TH17 and Treg cell differentiation. Nature.

[62] Jones BV, Begley M, Hill C, Gahan CG, Marchesi JR (2008). Functional and comparative metagenomic analysis of bile salt hydrolase activity in the human gut microbiome. Proc. Natl Acad. Sci. USA.

[63] Mythen, S. M., Devendran, S., Mendez-Garcia, C., Cann, I. & Ridlon, J. M. Targeted synthesis and characterization of a gene cluster encoding NAD(P)H-dependent 3alpha-, 3beta-, and 12alpha-hydroxysteroid dehydrogenases from Eggerthella CAG:298, a gut metagenomic sequence. *Appl. Environ. Microbiol.* **84** (2018). 10.1128/AEM.02475-17 PMC5861830 29330189

[64] Song Z (2019). Taxonomic profiling and populational patterns of bacterial bile salt hydrolase (BSH) genes based on worldwide human gut microbiome. Microbiome.

[65] Wirbel J (2019). Meta-analysis of fecal metagenomes reveals global microbial signatures that are specific for colorectal cancer. Nat. Med..

[66] Wegner K (2017). Rapid analysis of bile acids in different biological matrices using LC-ESI-MS/MS for the investigation of bile acid transformation by mammalian gut bacteria. Anal. Bioanal. Chem..

[67] Mullish, B. H. et al. Microbial bile salt hydrolases mediate the efficacy of faecal microbiota transplant in the treatment of recurrent Clostridioides difficile infection. *Gut* **68**, 1791–1800 (2019). 10.1136/gutjnl-2018-317842 PMC6839797 30816855

[68] Sun X (2018). Microbiota-derived metabolic factors reduce campylobacteriosis in mice. Gastroenterology.

[69] Gu Y (2017). Analyses of gut microbiota and plasma bile acids enable stratification of patients for antidiabetic treatment. Nat. Commun..

[70] Ma C (2018). Gut microbiome-mediated bile acid metabolism regulates liver cancer via NKT cells. Science.

[71] Paramsothy S (2019). Specific bacteria and metabolites associated with response to fecal microbiota transplantation in patients with ulcerative colitis. Gastroenterology.

[72] Devendran S (2019). Clostridium scindens ATCC 35704: integration of nutritional requirements, the complete genome sequence, and global transcriptional responses to bile acids. Appl. Environ. Microbiol..

[73] Devendran S, Mythen SM, Ridlon JM (2018). The desA and desB genes from Clostridium scindens ATCC 35704 encode steroid-17,20-desmolase. J. Lipid Res..

[74] Ridlon JM (2013). Clostridium scindens: a human gut microbe with a high potential to convert glucocorticoids into androgens. J. Lipid Res..

[75] Harris SC (2018). Identification of a gene encoding a flavoprotein involved in bile acid metabolism by the human gut bacterium Clostridium scindens ATCC 35704. Biochim. Biophys. Acta Mol. Cell Biol. Lipids.

[76] Martino ME (2018). Bacterial Adaptation to the host’s diet is a key evolutionary force shaping Drosophila-Lactobacillus symbiosis. Cell Host Microbe.

[77] Zhao S (2019). Adaptive evolution within gut microbiomes of healthy people. Cell Host Microbe.

[78] Bello MGD, Knight R, Gilbert JA, Blaser MJ (2018). Preserving microbial diversity. Science.

[79] Sonnenburg ED (2016). Diet-induced extinctions in the gut microbiota compound over generations. Nature.

[80] Gaulke CA (2018). Ecophylogenetics clarifies the evolutionary association between mammals and their gut microbiota. MBio.

[81] Seedorf H (2014). Bacteria from diverse habitats colonize and compete in the mouse gut. Cell.

[82] Leuchs S (2012). Inactivation and inducible oncogenic mutation of p53 in gene targeted pigs. PLoS ONE.

[83] Pawlowsky K (2017). The Aachen minipig: phenotype, genotype, hematological and biochemical characterization, and comparison to the Göttingen minipig. Eur. Surg. Res..

[84] Neumann AP, McCormick CA, Suen G (2017). Fibrobacter communities in the gastrointestinal tracts of diverse hindgut-fermenting herbivores are distinct from those of the rumen. Environ. Microbiol..

[85] McDonald JA (2013). Evaluation of microbial community reproducibility, stability and composition in a human distal gut chemostat model. J. Microbiol. Methods.

[86] Looft T, Levine UY, Stanton TB (2013). Cloacibacillus porcorum sp. nov., a mucin-degrading bacterium from the swine intestinal tract and emended description of the genus Cloacibacillus. Int. J. Syst. Evol. Microbiol..

[87] Postgate J (1963). Versatile medium for the enumeration of sulfate-reducing bacteria. Appl. Environ. Microbiol..

[88] Arfken AM, Frey JF, Ramsay TG, Summers KL (2019). Yeasts of burden: exploring the mycobiome-bacteriome of the piglet GI tract. Front. Microbiol..

[89] Gresse R, Chaucheyras Durand F, Duniere L, Blanquet-Diot S, Forano E (2019). Microbiota composition and functional profiling throughout the gastrointestinal tract of commercial weaning piglets. Microorganisms.

[90] Kwok KTT, Nieuwenhuijse DF, Phan MVT, Koopmans MPG (2020). Virus metagenomics in farm animals: a systematic review. Viruses.

[91] Norman JM (2015). Disease-specific alterations in the enteric virome in inflammatory bowel disease. Cell.

[92] van Tilburg Bernardes E (2020). Intestinal fungi are causally implicated in microbiome assembly and immune development in mice. Nat. Commun..

[93] Lagier JC (2016). Culture of previously uncultured members of the human gut microbiota by culturomics. Nat. Microbiol..

[94] Poyet M (2019). A library of human gut bacterial isolates paired with longitudinal multiomics data enables mechanistic microbiome research. Nat. Med..

[95] Zou Y (2019). 1,520 reference genomes from cultivated human gut bacteria enable functional microbiome analyses. Nat. Biotechnol..

[96] Costea PI (2017). Subspecies in the global human gut microbiome. Mol. Syst. Biol..

[97] Karcher N (2020). Analysis of 1321 Eubacterium rectale genomes from metagenomes uncovers complex phylogeographic population structure and subspecies functional adaptations. Genome Biol..

[98] Attebery HR, Finegold SM (1969). Combined screw-cap and rubber-stopper closure for Hungate tubes (pre-reduced anaerobically sterilized roll tubes and liquid media). Appl. Microbiol..

[99] Greuter D, Loy A, Horn M, Rattei T (2016). probeBase–an online resource for rRNA-targeted oligonucleotide probes and primers: new features 2016. Nucleic Acids Res..

[100] Yoon SH (2017). Introducing EzBioCloud: a taxonomically united database of 16S rRNA gene sequences and whole-genome assemblies. Int. J. Syst. Evol. Microbiol..

[101] Kumar S, Stecher G, Li M, Knyaz C, Tamura K (2018). MEGA X: molecular evolutionary genetics analysis across computing platforms. Mol. Biol. Evol..

[102] Jain C, Rodriguez RL, Phillippy AM, Konstantinidis KT, Aluru S (2018). High throughput ANI analysis of 90K prokaryotic genomes reveals clear species boundaries. Nat. Commun..

[103] Altschul SF, Gish W, Miller W, Myers EW, Lipman DJ (1990). Basic local alignment search tool. J. Mol. Biol..

[104] Qin QL (2014). A proposed genus boundary for the prokaryotes based on genomic insights. J. Bacteriol..

[105] Streidl, T., Kumar, N., Navaro Suarez, L., Rohn, S. & Clavel, T. In *Bergey’s Manual of Systematic Bacteriology* (John Wiley & Sons, Inc., 2019).

[106] Alvarez L, Hernandez SB, de Pedro MA, Cava F (2016). Ultra-sensitive, high-resolution liquid chromatography methods for the high-throughput quantitative analysis of bacterial cell wall chemistry and structure. Methods Mol. Biol..

[107] Jutras BL (2019). Borrelia burgdorferi peptidoglycan is a persistent antigen in patients with Lyme arthritis. Proc. Natl Acad. Sci. USA.

[108] Hernández SB, Dörr T, Waldor MK, Cava F (2020). Modulation of peptidoglycan synthesis by recycled cell wall tetrapeptides. Cell Rep..

[109] Pruesse E (2007). SILVA: a comprehensive online resource for quality checked and aligned ribosomal RNA sequence data compatible with ARB. Nucleic Acids Res..

[110] Edgar RC (2010). Search and clustering orders of magnitude faster than BLAST. Bioinformatics.

[111] Gonzalez-Prendes R (2019). Modulatory effect of protein and carotene dietary levels on pig gut microbiota. Sci. Rep..

[112] Abbott A (2015). Inside the first pig biobank. Nature.

[113] Reitmeier, S. et al. Arrhythmic gut microbiome signatures predict risk of Type 2 diabetes. *Cell Host Microbe* **28**, 258–272.e6 (2020). 10.1016/j.chom.2020.06.004 32619440

[114] Lagkouvardos I, Fischer S, Kumar N, Clavel T (2017). Rhea: a transparent and modular R pipeline for microbial profiling based on 16S rRNA gene amplicons. PeerJ.

[115] Godon JJ, Zumstein E, Dabert P, Habouzit F, Moletta R (1997). Molecular microbial diversity of an anaerobic digestor as determined by small-subunit rDNA sequence analysis. Appl. Environ. Microbiol..

[116] Berry D, Ben Mahfoudh K, Wagner M, Loy A (2011). Barcoded primers used in multiplex amplicon pyrosequencing bias amplification. Appl. Environ. Microbiol..

[117] Klindworth A (2013). Evaluation of general 16S ribosomal RNA gene PCR primers for classical and next-generation sequencing-based diversity studies. Nucleic Acids Res..

[118] Huptas, C., Scherer, S. & Wenning, M. Optimized Illumina PCR-free library preparation for bacterial whole genome sequencing and analysis of factors influencing de novo assembly. *BMC Res. Notes* **9**, 269 (2016). 10.1186/s13104-016-2072-9 PMC4864918 27176120

[119] Li H, Durbin R (2009). Fast and accurate short read alignment with Burrows-Wheeler transform. Bioinformatics.

[120] Seemann T (2014). Prokka: rapid prokaryotic genome annotation. Bioinformatics.

[121] Bankevich A (2012). SPAdes: a new genome assembly algorithm and its applications to single-cell sequencing. J. Comput. Biol..

[122] Gurevich A, Saveliev V, Vyahhi N, Tesler G (2013). QUAST: quality assessment tool for genome assemblies. Bioinformatics.

[123] Parks DH, Imelfort M, Skennerton CT, Hugenholtz P, Tyson GW (2015). CheckM: assessing the quality of microbial genomes recovered from isolates, single cells, and metagenomes. Genome Res..

[124] Ogata H (1999). KEGG: Kyoto Encyclopedia of Genes and Genomes. Nucleic Acids Res..

[125] McArthur AG (2013). The comprehensive antibiotic resistance database. Antimicrob. Agents Chemother..

[126] Huerta-Cepas J (2017). Fast genome-wide functional annotation through orthology assignment by eggNOG-Mapper. Mol. Biol. Evol..

[127] Emms DM, Kelly S (2015). OrthoFinder: solving fundamental biases in whole genome comparisons dramatically improves orthogroup inference accuracy. Genome Biol..

[128] Huerta-Cepas J (2016). eggNOG 4.5: a hierarchical orthology framework with improved functional annotations for eukaryotic, prokaryotic and viral sequences. Nucleic Acids Res..

[129] Buchfink B, Xie C, Huson DH (2015). Fast and sensitive protein alignment using DIAMOND. Nat. Methods.

[130] Yang J, Zhang Y (2015). I-TASSER server: new development for protein structure and function predictions. Nucleic Acids Res..

[131] Heine V (2019). Identifying efficient Clostridium difficile toxin A binders with a multivalent neo-glycoprotein glycan library. Bioconjugate Chem..

[132] Blin K (2019). antiSMASH 5.0: updates to the secondary metabolite genome mining pipeline. Nucleic Acids Res..

[133] Sievers F, Higgins DG (2014). Clustal Omega, accurate alignment of very large numbers of sequences. Methods Mol. Biol..

[134] Crooks GE, Hon G, Chandonia JM, Brenner SE (2004). WebLogo: a sequence logo generator. Genome Res..

[135] Li D (2016). MEGAHIT v1.0: a fast and scalable metagenome assembler driven by advanced methodologies and community practices. Methods.

[136] Olm MR, Brown CT, Brooks B, Banfield JF (2017). dRep: a tool for fast and accurate genomic comparisons that enables improved genome recovery from metagenomes through de-replication. ISME J..

[137] Kumar S, Stecher G, Tamura K (2016). MEGA7: Molecular Evolutionary Genetics Analysis Version 7.0 for bigger datasets. Mol. Biol. Evol..

[138] Saitou N, Nei M (1987). The neighbor-joining method: a new method for reconstructing phylogenetic trees. Mol. Biol. Evol..

[139] Zuckerkandl E, Pauling L (1965). Molecules as documents of evolutionary history. J. Theor. Biol..

[140] Bushnell, B. BBMap: A Fast, Accurate, Splice-Aware Aligner. No. LBNL-7065E. Ernest Orlando Lawrence Berkeley National Laboratory (CA, United states, 2014).

[141] Ridlon, J. M. et al. The ‘in vivo lifestyle’ of bile acid 7alpha-dehydroxylating bacteria: comparative genomics, metatranscriptomic, and bile acid metabolomics analysis of a defined microbial community in gnotobiotic mice. *Gut Microbes* **11**, 1–24 (2019). 10.1080/19490976.2019.1618173 PMC7524365 31177942

[142] Yarza P (2014). Uniting the classification of cultured and uncultured bacteria and archaea using 16S rRNA gene sequences. Nat. Rev. Microbiol..

[143] Meier-Kolthoff JP, Klenk HP, Goker M (2014). Taxonomic use of DNA G+C content and DNA-DNA hybridization in the genomic age. Int. J. Syst. Evol. Microbiol..

[144] Nouioui I (2018). Genome-based taxonomic classification of the Phylum Actinobacteria. Front. Microbiol..

[145] Mishra AK, Hugon P, Robert C, Raoult D, Fournier PE (2012). Non contiguous-finished genome sequence and description of Peptoniphilus grossensis sp. nov. Stand. Genomic Sci..

[146] Willems A, Collins MD (1995). 16S rRNA gene similarities indicate that Hallella seregens (Moore and Moore) and Mitsuokella dentalis (Haapsalo et al.) are genealogically highly related and are members of the genus Prevotella: emended description of the genus Prevotella (Shah and Collins) and description of Prevotella dentalis comb. nov. Int. J. Syst. Bacteriol..

[147] Rosero JA (2016). Reclassification of Eubacterium rectale (Hauduroy et al. 1937) Prevot 1938 in a new genus Agathobacter gen. nov. as Agathobacter rectalis comb. nov., and description of Agathobacter ruminis sp. nov., isolated from the rumen contents of sheep and cows. Int. J. Syst. Evol. Microbiol..

[148] Rosero, J. A. et al. Reply to the Letter to the Editor by Paul O. Sheridan, Sylvia H. Duncan, Alan W. Walker, Karen P. Scott, Petra Louis and Harry J. Flint, referring to our paper ‘Reclassification of Eubacterium rectale (Prevot et al. 1967) in a new genus Agathobacter gen. nov., as Agathobacter rectalis comb. nov., within the family Lachnospiraceae, and description of Agathobacter ruminis sp. nov., from the rumen’. *Int. J. Syst. Evol. Microbiol.* **66**, 2107 (2016). 10.1099/ijsem.0.000993 26920933

[149] Sheridan PO (2016). Objections to the proposed reclassification of Eubacterium rectale as Agathobacter rectalis. Int. J. Syst. Evol. Microbiol..

[150] Zuo G, Hao B (2016). Whole-genome-based phylogeny supports the objections against the reclassification of Eubacterium rectale to Agathobacter rectalis. Int. J. Syst. Evol. Microbiol..

