# Supplementary material for: A collection of bacterial isolates from the pig intestine reveals functional and taxonomic diversity
Source: Nat Commun. 2020 Dec 15;11:6389. doi: 10.1038/s41467-020-19929-w (PMC7738495; doi:10.1038/s41467-020-19929-w)
Supplement: Supplementary file 1 — Supplementary Information [file 41467_2020_19929_MOESM1_ESM.pdf]

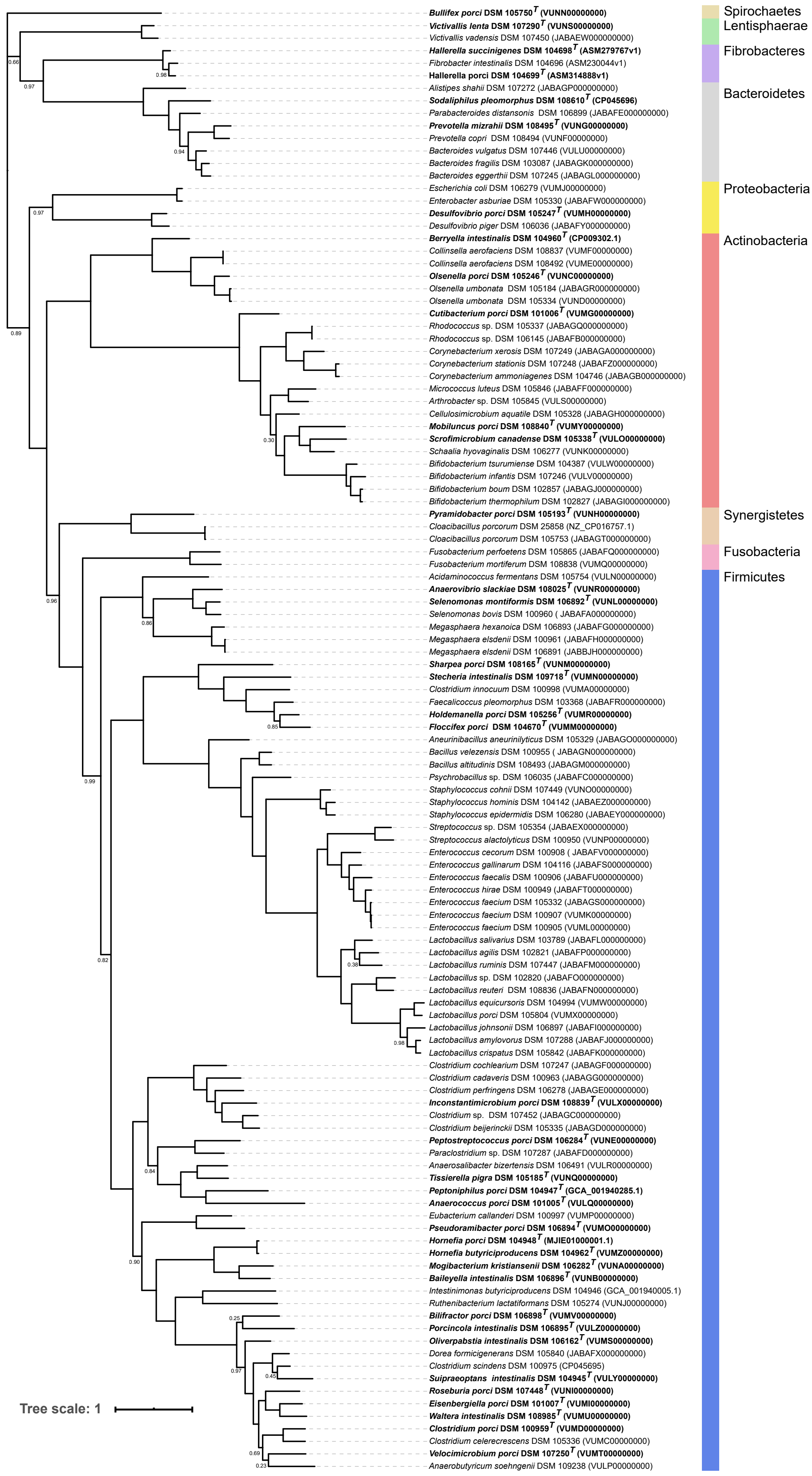

Supplementary Fig. 1 / Phlyogenomic tree of isolates within *PiBAC*.
The tree was constructed based on 400 marker genes in PhyloPhlan (v0.99).
The accession numbers of genomes used for the analysis are indicated in brackets next to species names.
Novel taxa are written in bold letters. Colors indicate phyla.

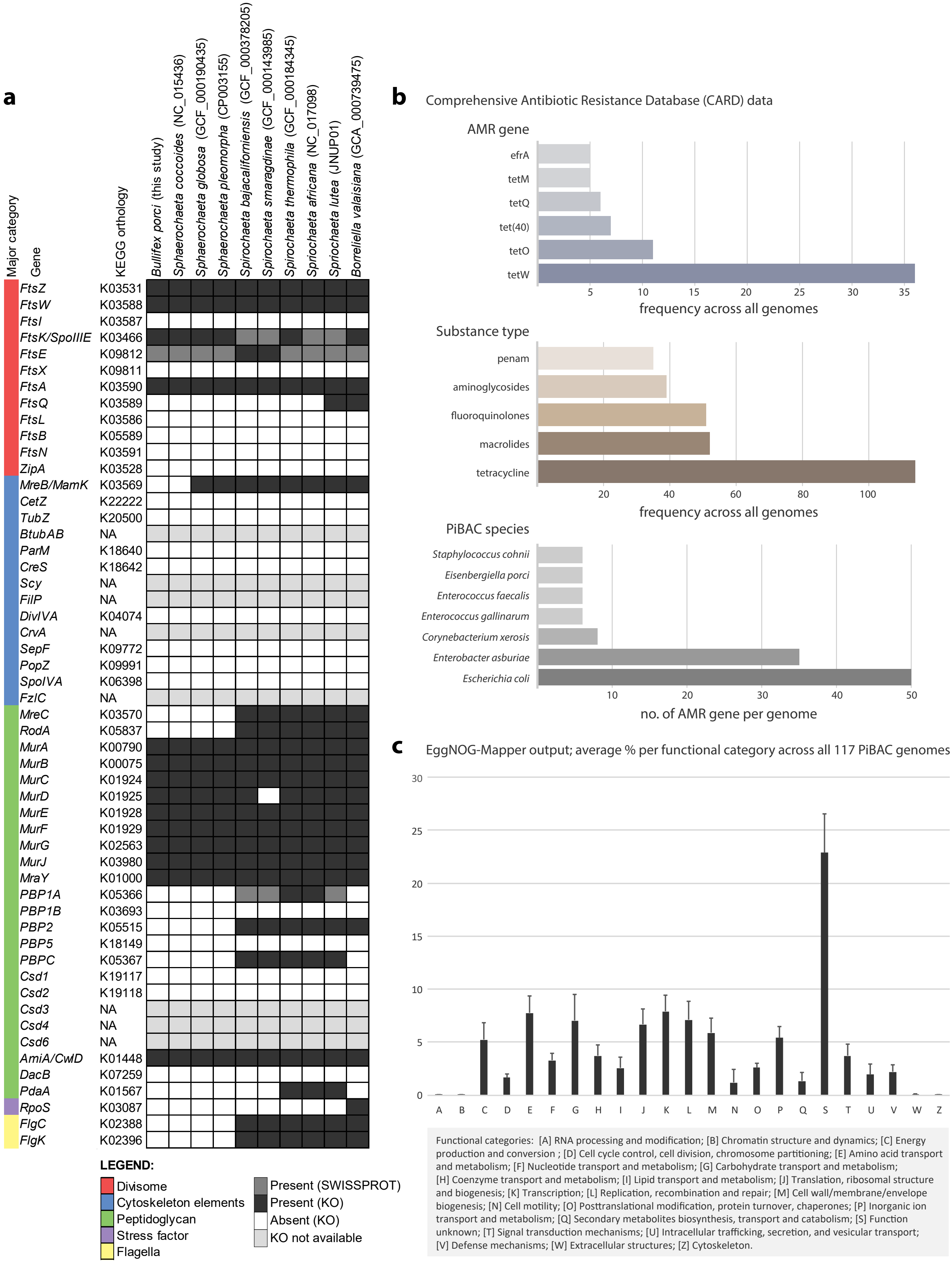

Supplementary Fig. 2 / Genome-based functional inisghts into PiBAC.
(a) Occurrence of genes involved in cell morphology and division, peptidoglycan synthesis, and cell wall formation within the genome of *Bullifex porci* gen. nov. sp. nov. and related species within the family *Spirochaetaceae*.
(b) List of most frequent antimicrobial resistance (AMR) genes (top; bluish bars) and antimicrobial substances (middle; brown) within the entire collection along with PiBAC species with highest numbers of AMR genes (bottom; gray).
(c) Global functional annotation of the 117 strains within PiBAC according to EggNOG categories. The bar chart shows average % per functional category by EggNOG-Mapper; data is presented as mean values ± SD.

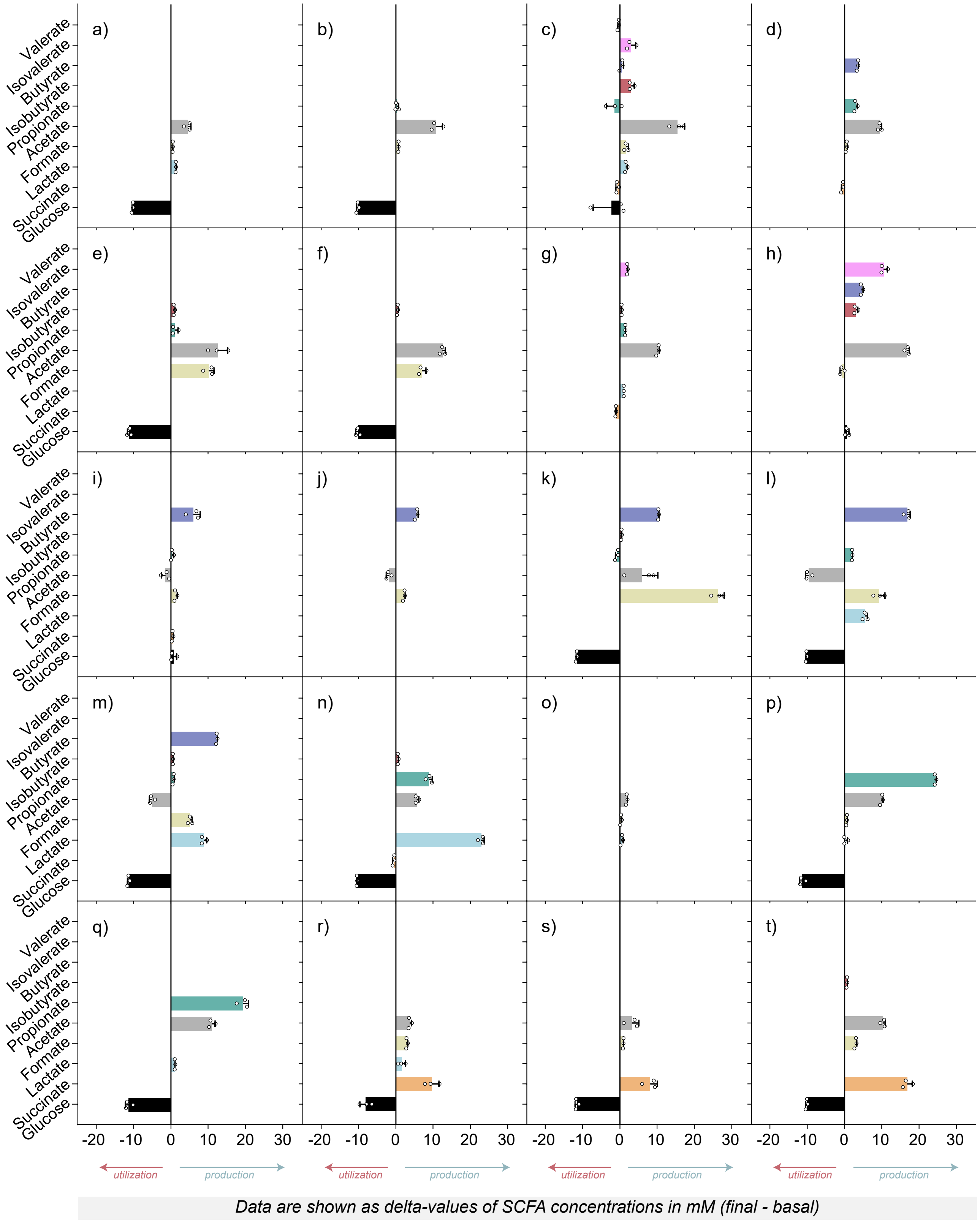

Supplementary Fig. 3 / Short-chain fatty acid (SCFA) profile of new taxa provided in PiBAC. Triplicate cultures of each strain were incubated in modified YCFA broth (DSMZ medium 1611). Unless otherwise stated in brackets, the incubation time was 48 h at 37 °C under anaerobic conditions. The concentrations of SCFA were determined by HPLC-RI as detailed in the Methods. Results are represented as mean with SD whereby delta-values below 0.5 mM (threshold) were not plotted.

Bacteria were: (a) *Victivalis lenta* DSM 107290; (b) *Suipraeoptans intestinalis* DSM 104945; (c) *Peptostreptococcus porci* DSM 106284; (d) *Peptoniphilus porci* DSM 104947; (e) *Velocimicrobium porci* DSM 107250; (f) *Clostridium porci* DSM 100959; (g) *Pyramidobacter porci* DSM 105193 (96 h); (h) *Tissierella pigra* DSM 105185; (i) *Hornefia butyriciproducens* DSM 104962; (j) *Hornefia porci* DSM 104948; (k) *Eisenbergiella porci* DSM 101007; (l) *Anaerococcus porci* DSM 101005; (m) *Roseburia porci* DSM 107448; (n) *Selenomonas montiformis* DSM 106892; (o) *Olsenella porci* DSM 105246 (96 h); (p) *Cutibacterium porci* DSM 101006; (q) *Anaerovibrio slackiae* DSM 108025; (r) *Scrofimicrobium canadense* DSM 105338; (s) *Hallerella succinigenes* DSM 104698; (t) *Prevotella mizrahii* DSM 108495.

Suppl. Fig. 4a 16S rRNA gene-based phylogenetic tree of *Berryella intestinalis* gen. nov., sp. nov. and *Olsenella porci* sp. nov.

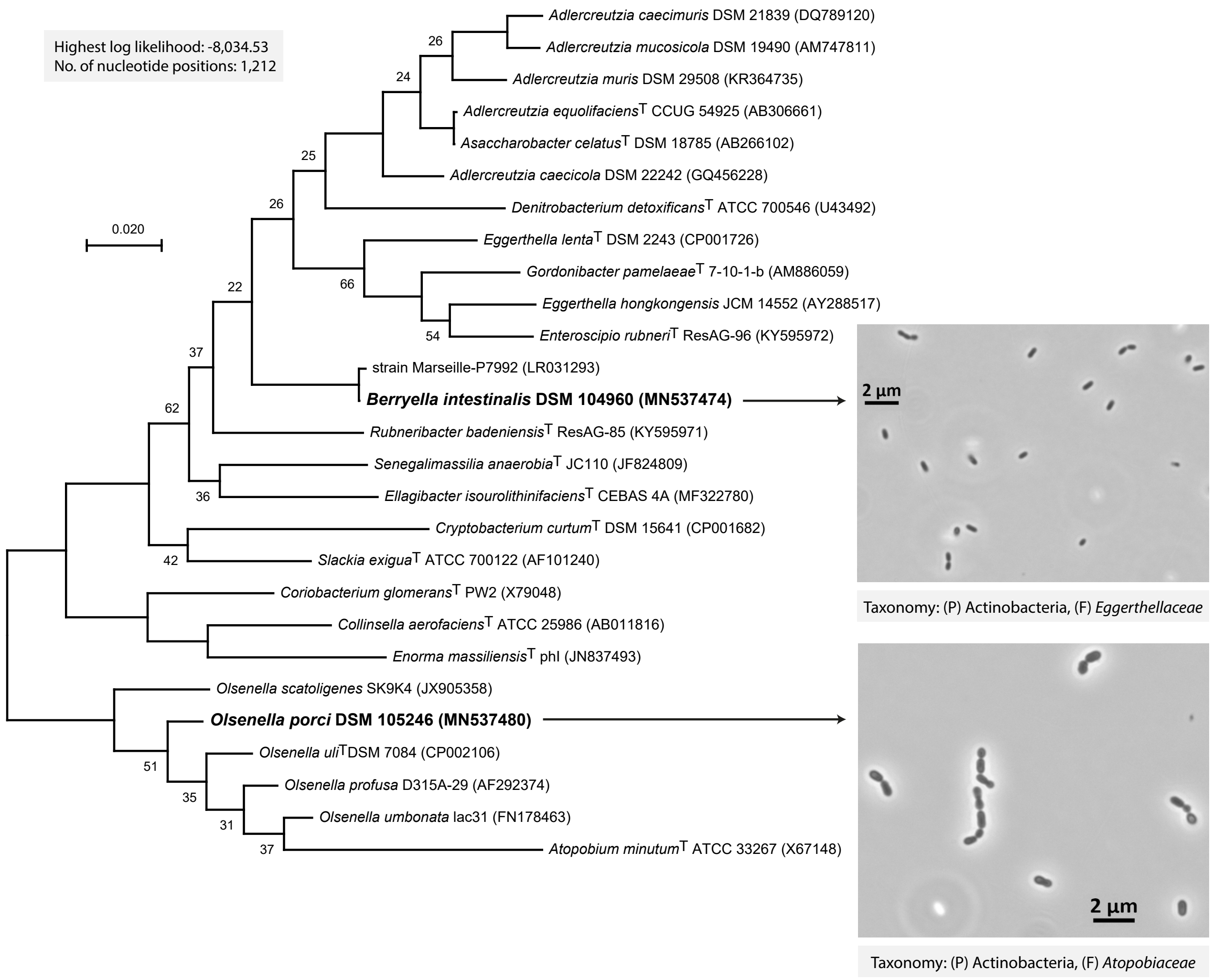

Suppl. Fig. 4b 16S rRNA gene-based phylogenetic tree of *Cutibacterium porci* sp. nov.

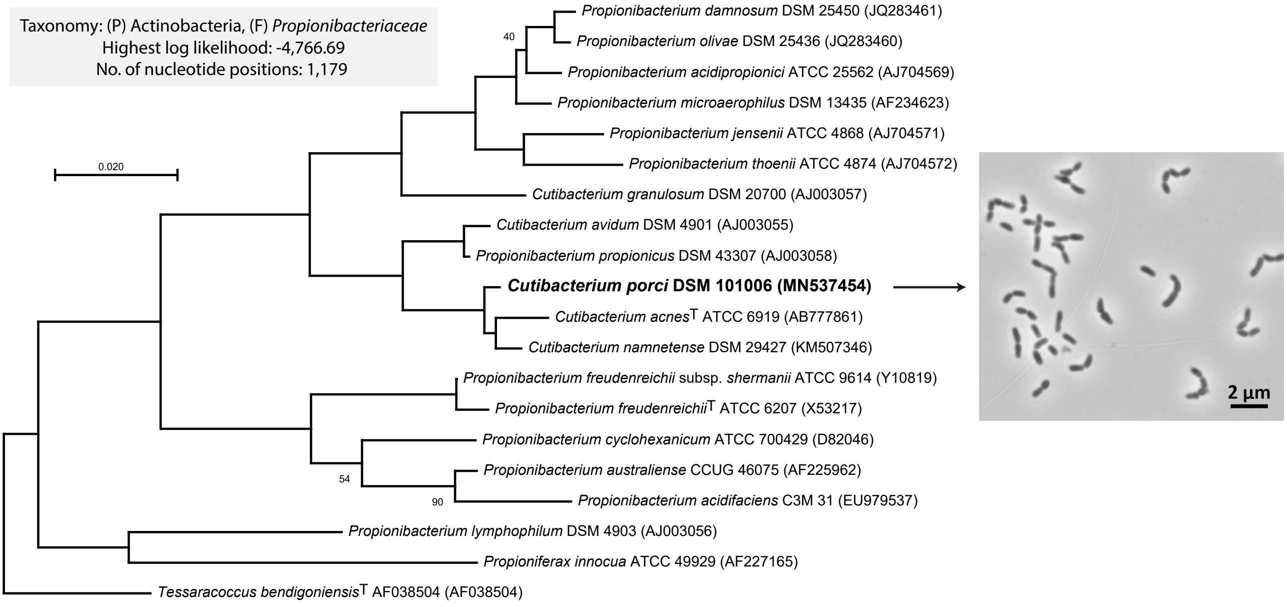

Suppl. Fig. 4c 16S rRNA gene-based phylogenetic tree of *Mobiluncus porci* sp. nov. and *Scrofimicrobium canadense* gen. nov., sp. nov.

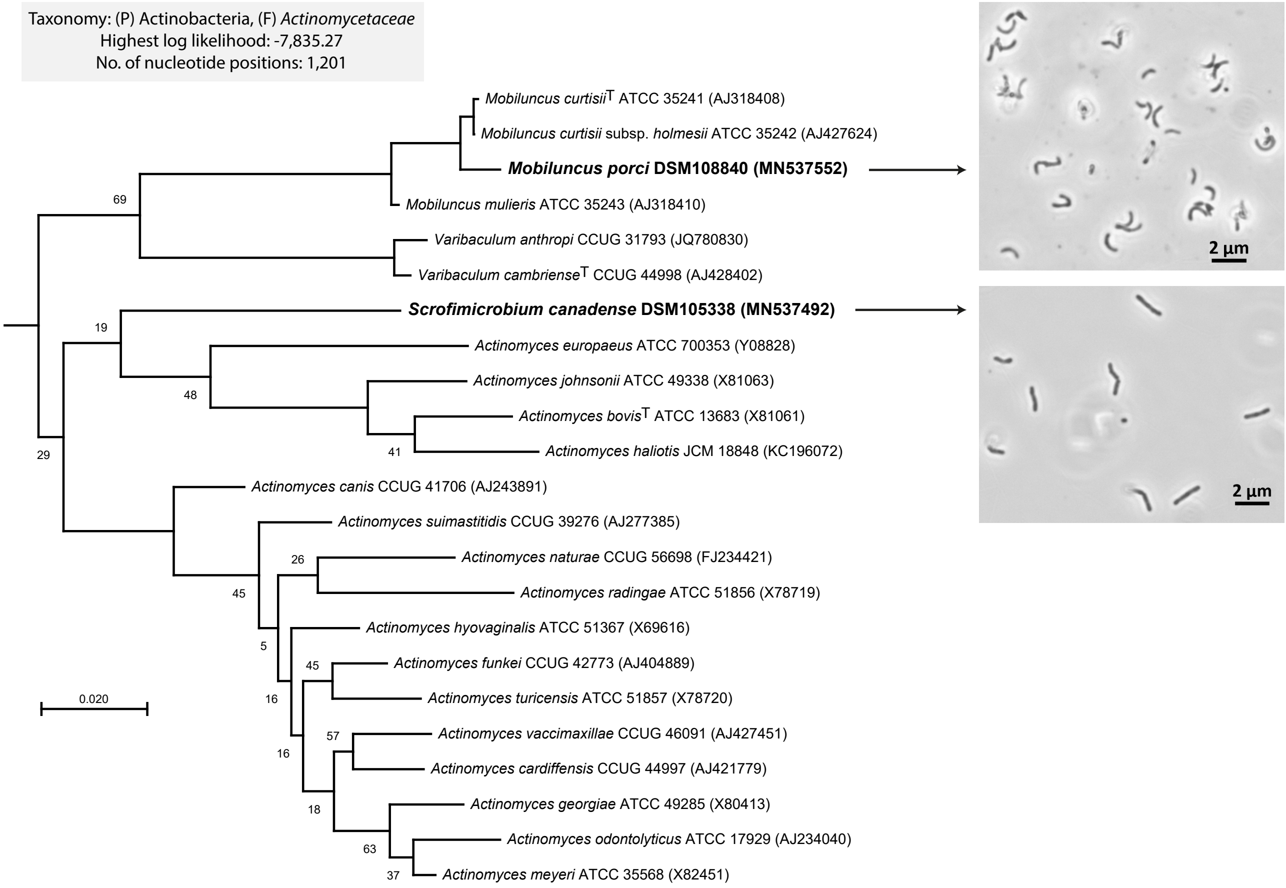

Suppl. Fig. 4d 16S rRNA gene-based phylogenetic tree of *Sodaliphilus pleomorphus* gen. nov., sp. nov.

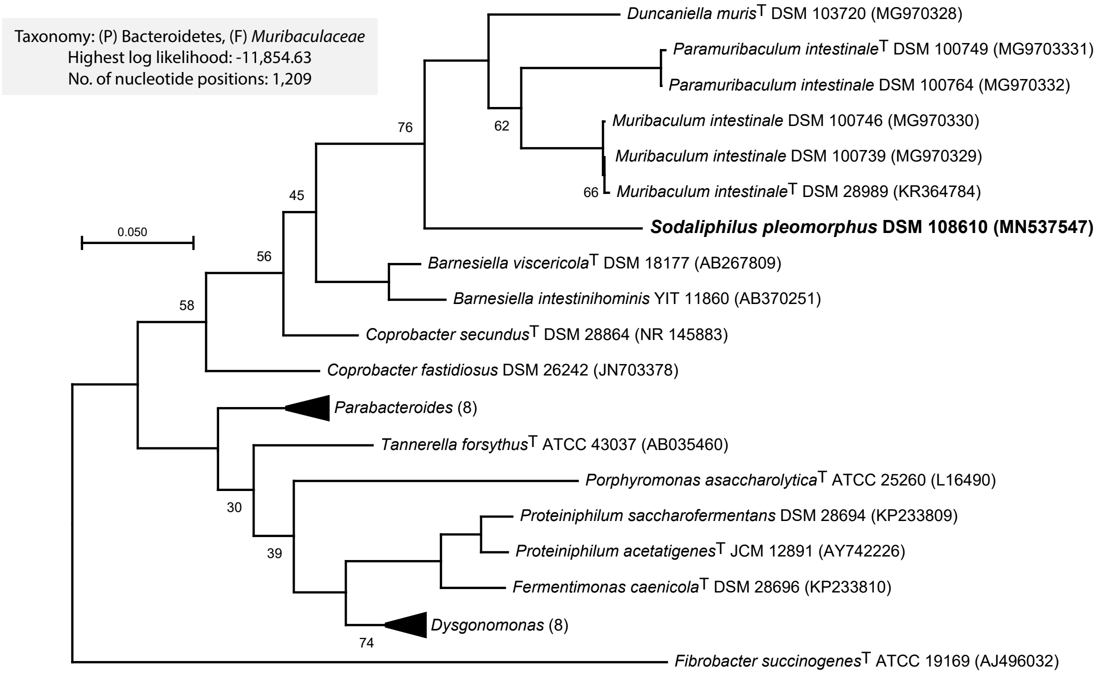

Suppl. Fig. 4e 16S rRNA gene-based phylogenetic tree of *Prevotella mizrahii* sp. nov.

Taxonomy: (P) Bacteroidetes, (F) *Prevotellaceae*
Highest log likelihood: -21,459.21
No. of nucleotide positions: 1,165

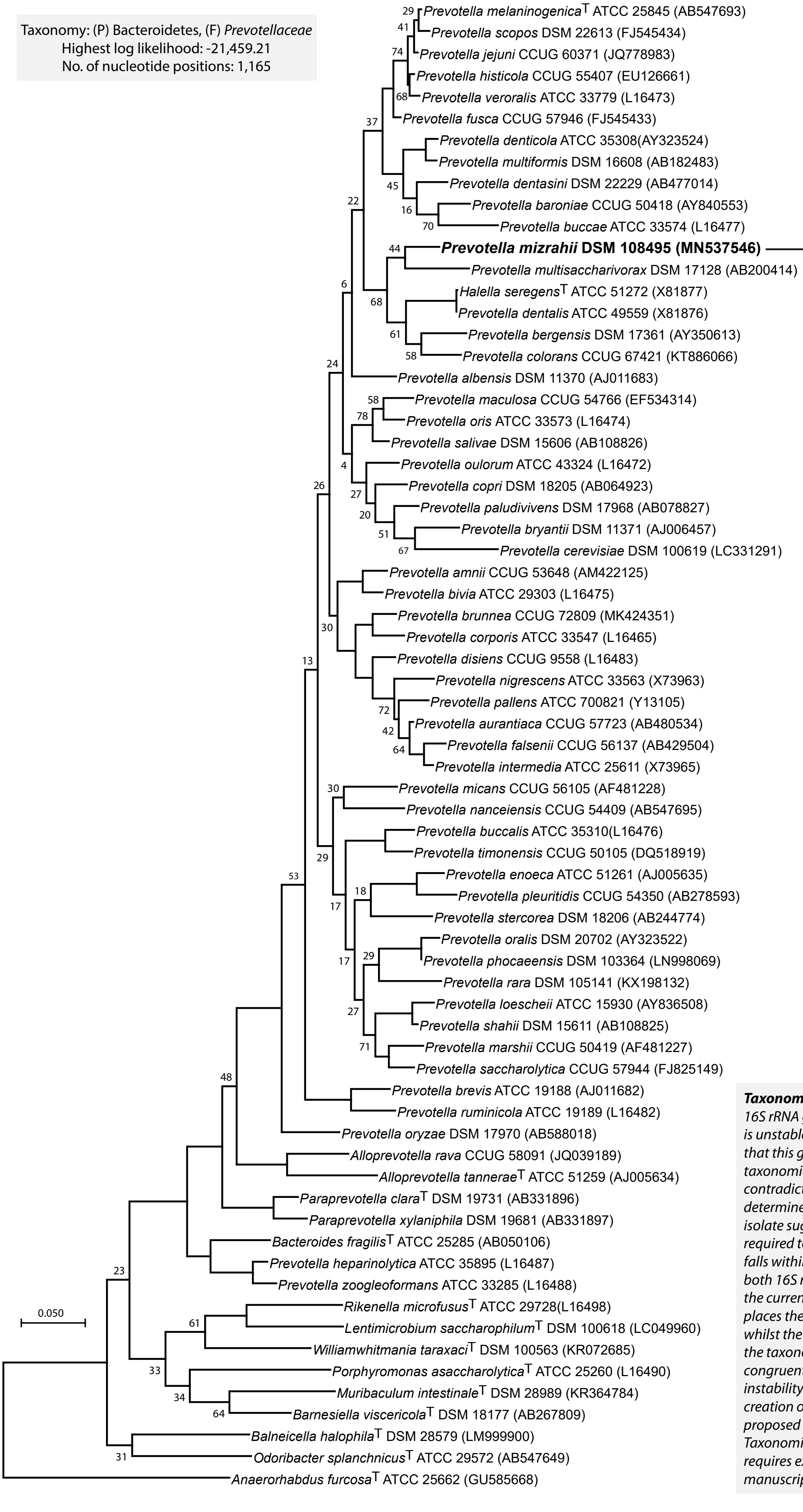

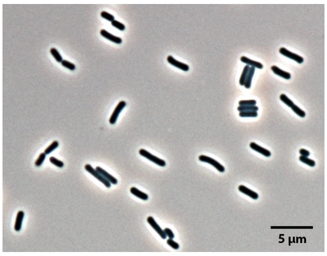

Taxonomic note:
16S rRNA gene-based phylogeny of the genus Prevotella is unstable and phylogenomic analysis clearly shows that this genus is heterogenous and requires extensive taxonomic amendments. Although somewhat contradictory, the various genome-based parameters determined to delineate the status of our isolate suggest that the creation of a novel genus is required to accomodate it. However, the strain clearly falls within the Prevotella genus cluster (according to both 16S rRNA gene-based and genomic trees) as per the current status of valid names, and GTFB-Tk also places the isolate within this genus. Furthermore, whilst the species name Hallella serengens is valid, the taxonomy of this and related species is not congruent. Altogether, to avoid generating further instability within the genus Prevotella, the creation of a novel species, Prevotella mizrahii, is proposed to accomodate strains DSM 108495. Taxonomic reclassifcations within the genus Prevotella requires extensive work. It is out of scope of the present manuscript and will be performed in future studies.

Suppl. Fig. 4f 16S rRNA gene-based phylogenetic tree of *Inconstantimicrobium porci* gen nov., sp. nov.

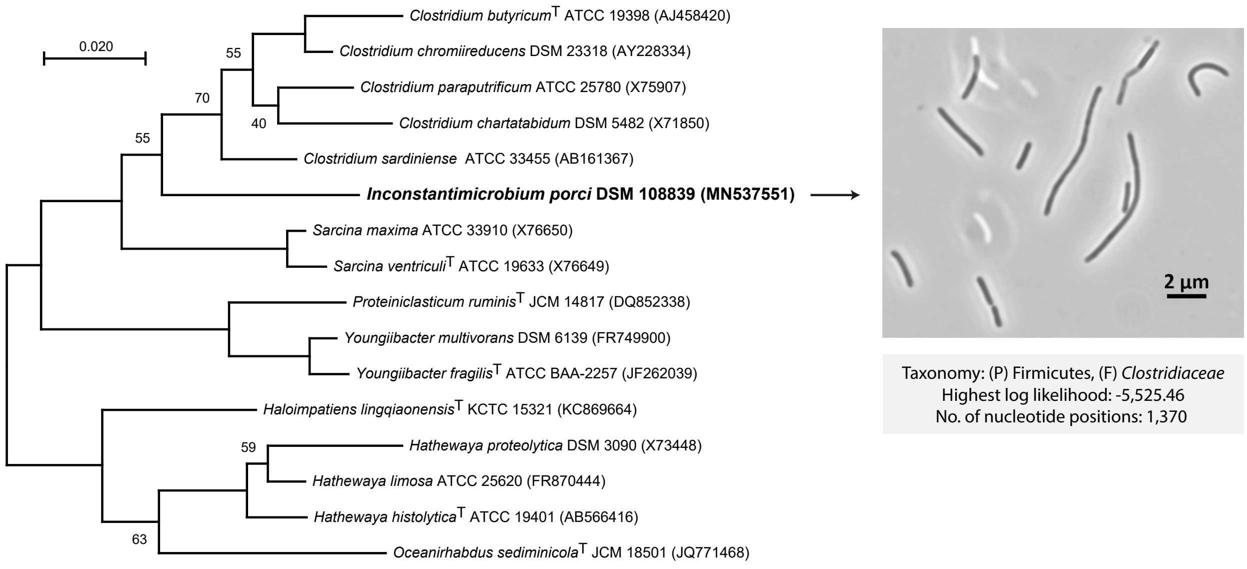

Suppl. Fig. 4g 16S rRNA gene-based phylogenetic tree of *Pseudoramibacter porci* sp. nov.

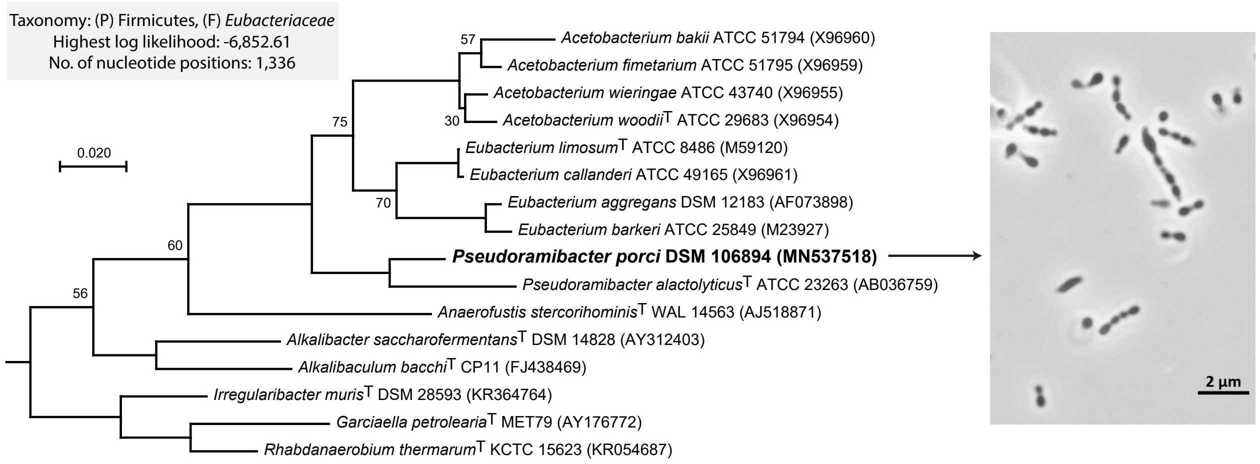

Suppl. Fig. 4h 16S rRNA gene-based phylogenetic tree of *Tissierella pigra* sp. nov.

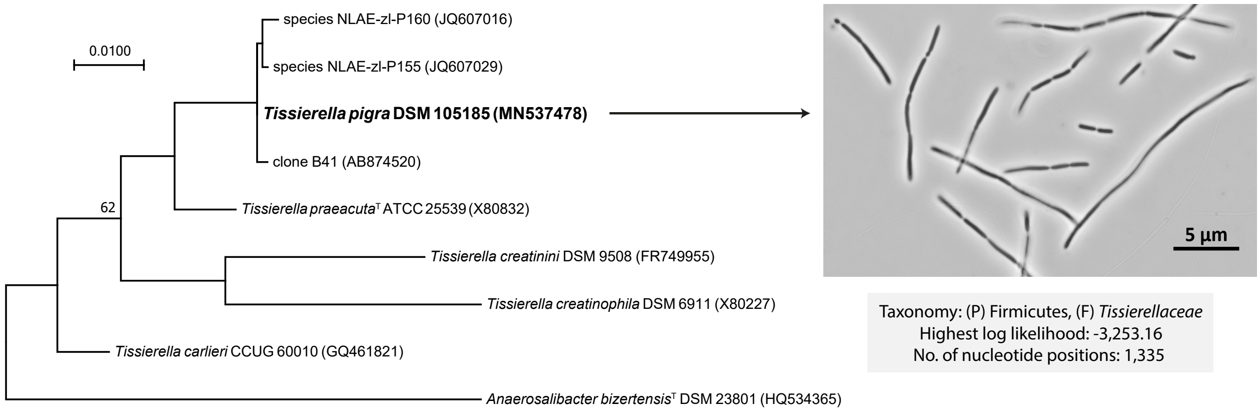

Suppl. Fig. 4i 16S rRNA-based phylogenetic tree of *Floccifex porci* gen. nov., sp. nov., *Holdemanella porci* sp. nov., *Stecheria intestinalis* gen. nov., sp. nov., and *Sharpea porci* sp. nov.

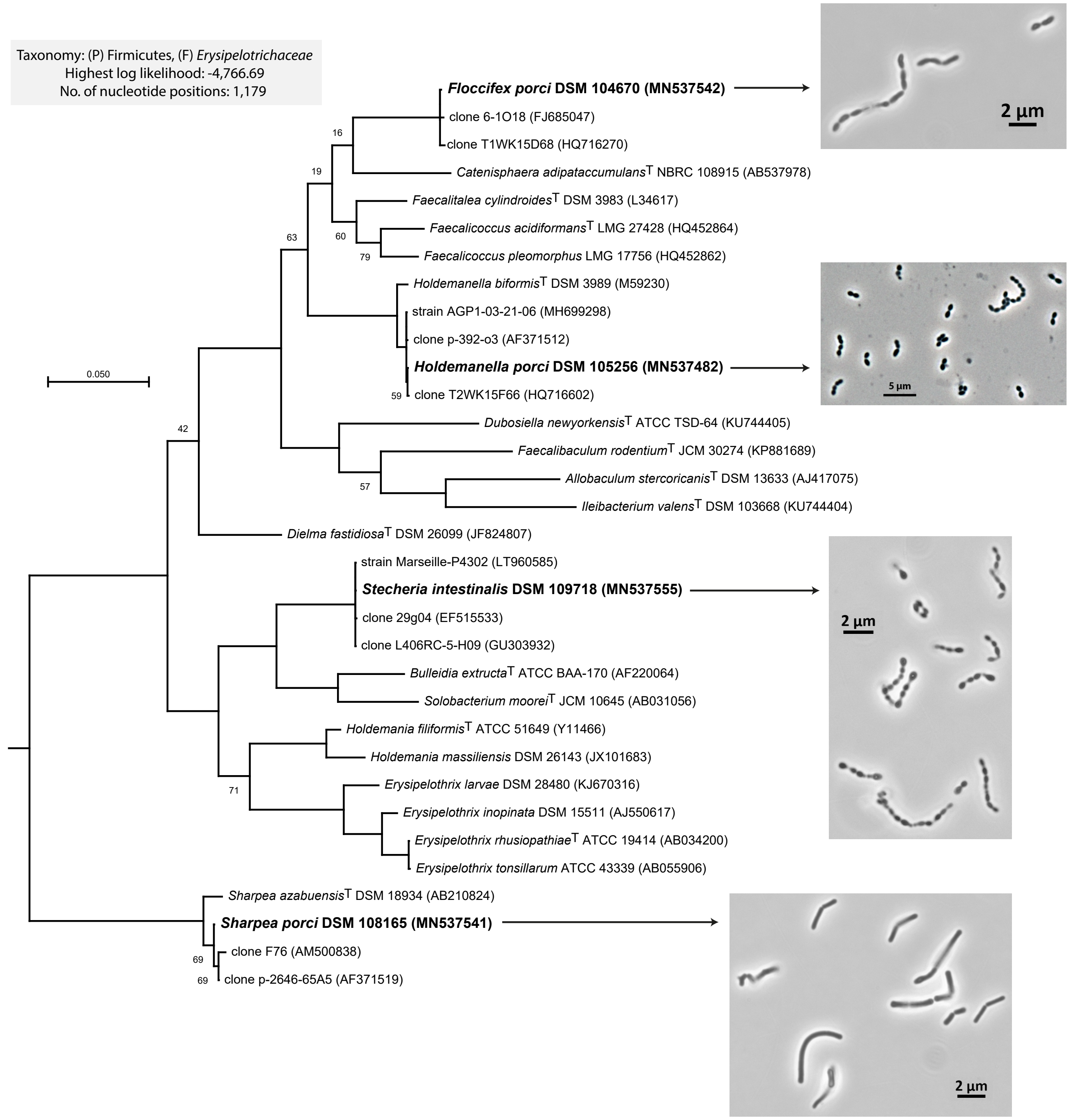

Suppl. Fig. 4j 16S rRNA gene-based phylogenetic tree of *Roseburia porci* sp. nov.

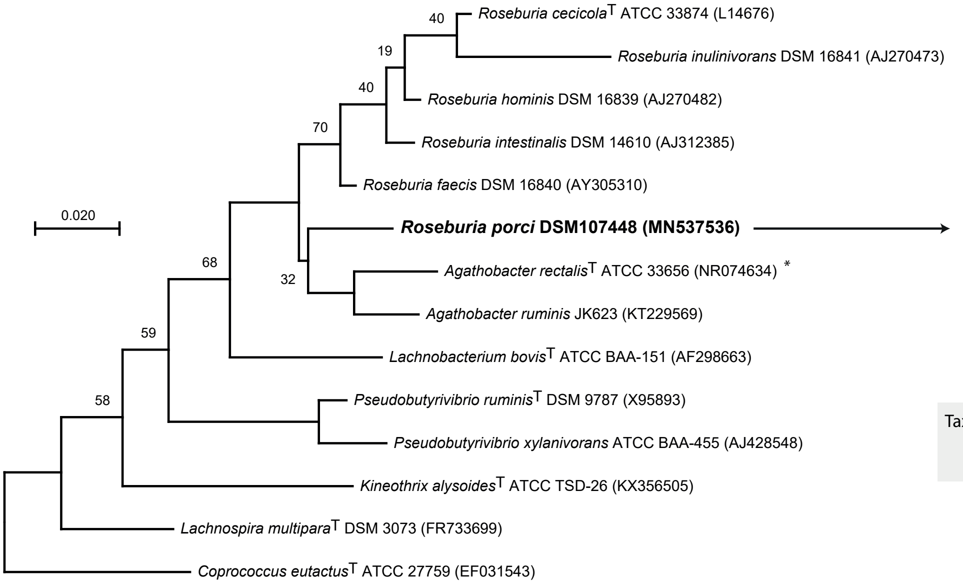

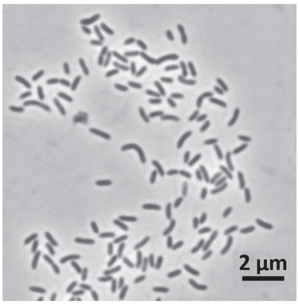

Taxonomy: (P) Firmicutes, (F) *Lachnospiraceae*
Highest log likelihood: -4,672.98
No. of nucleotide positions: 1,240

** The proposition to accomodate Eubacterium rectale into the genus Agathobacter[1] has been thereafter refuted by genome analysis[2], which indicated that the species is located deeply inside the Roseburia cluster. There is no genome available for Agathobacter ruminis, preventing further analysis. The taxonomic status of Agathobacter spp. is thus ambiguous and requires amendment.*

Suppl. Fig. 4k 16S rRNA gene-based phylogenetic tree of *Bilifractor porci* gen. nov., sp. nov., *Porcincola intestinalis* gen. nov., sp. nov. and *Oliverpabstia intestinalis* gen. nov., sp. nov.

Taxonomy: (P) Firmicutes, (F) *Lachnospiraceae*
Highest log likelihood: -7,422.63
No. of nucleotide positions: 1,029

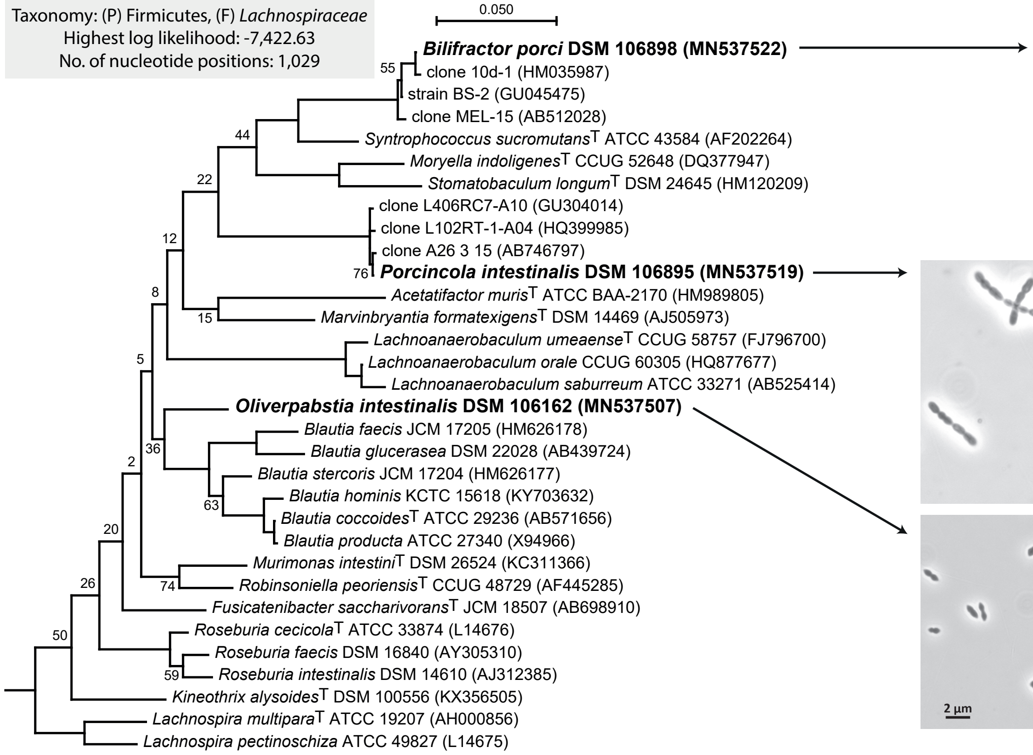

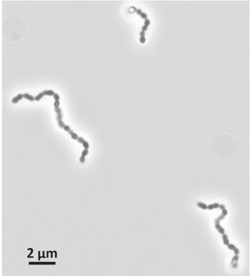

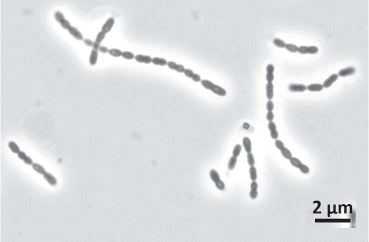

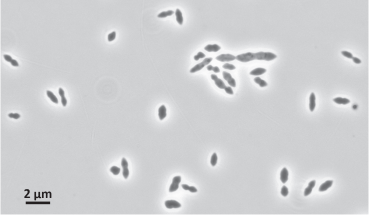

Suppl. Fig. 4l 16S rRNA gene-based phylogenetic tree of *Eisenbergiella porci* sp. nov., *Velocimicrobium porci* gen. nov., sp. nov. and *Waltera intestinalis* gen. nov., sp. nov.

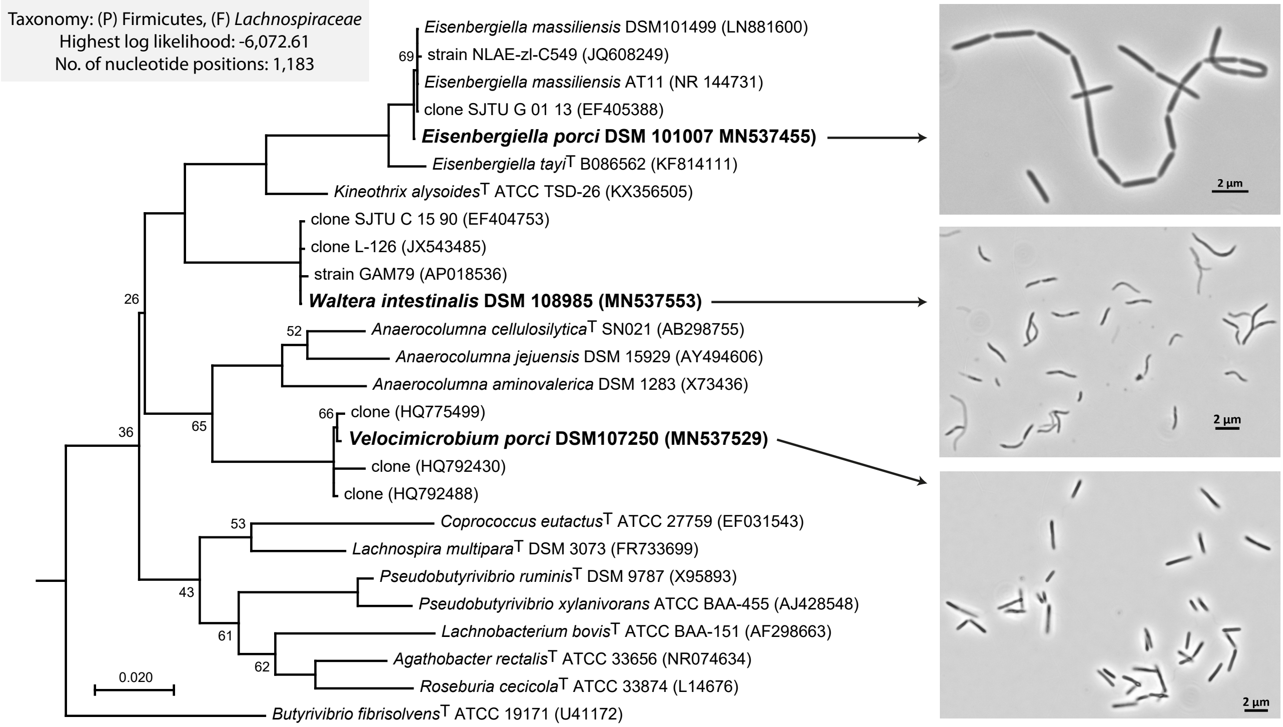

Suppl. Fig. 4m 16S rRNA gene-based phylogenetic tree of *Suipraeoptans intestinalis* gen nov., sp. nov.

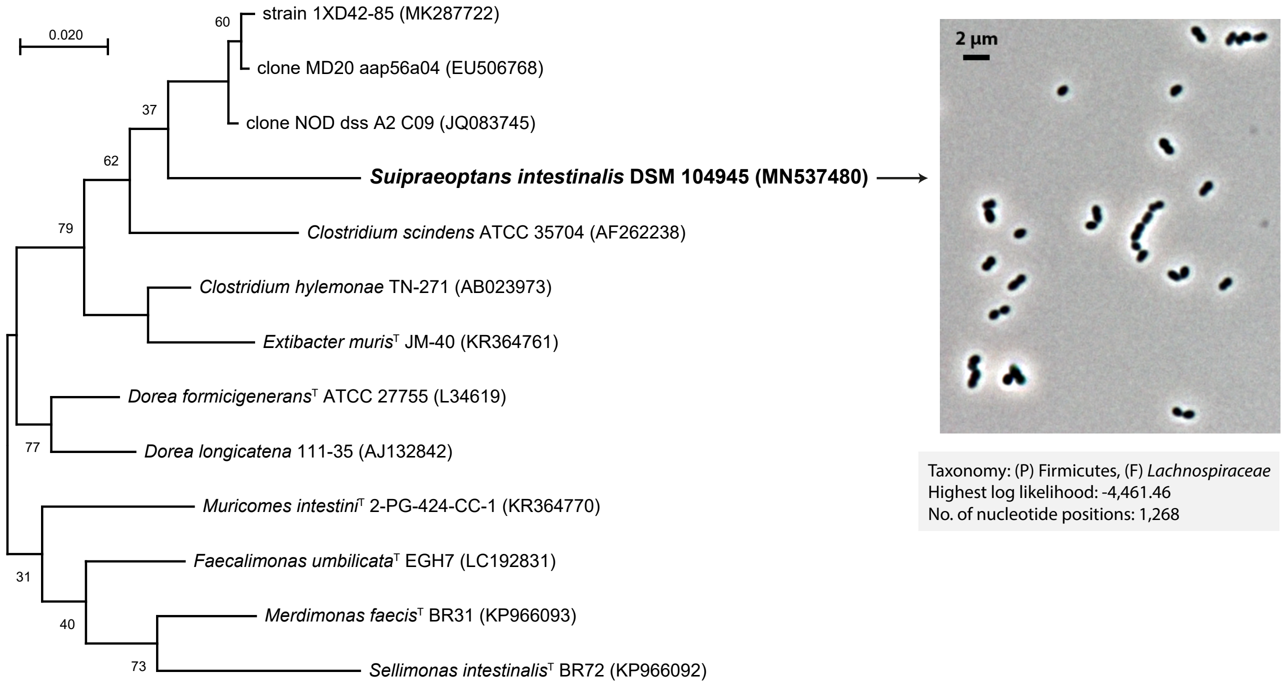

Suppl. Fig. 4n 16S rRNA gene-based phylogenetic tree of *Clostridium porci* sp. nov.

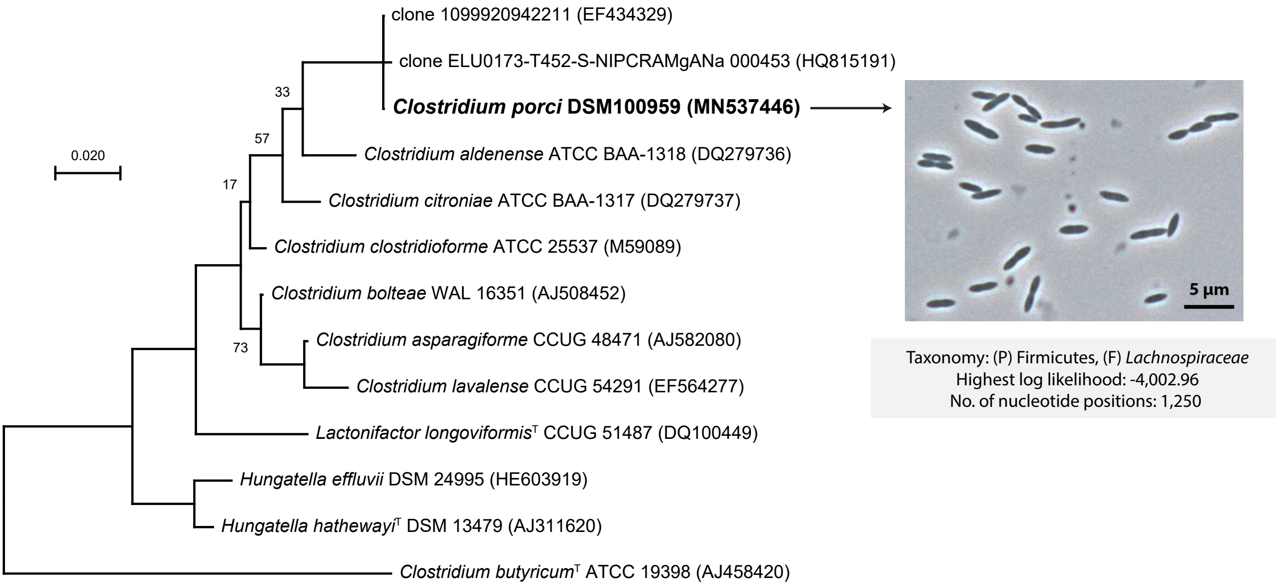

Suppl. Fig. 4o 16S rRNA gene-based phylogenetic tree of *Mogibacterium kristiansenii* sp. nov., *Baileyella intestinalis* gen. nov., sp. nov., *Hornefia butyriciproducens* gen. nov., sp. nov., and *Hornefia porci* sp. nov.

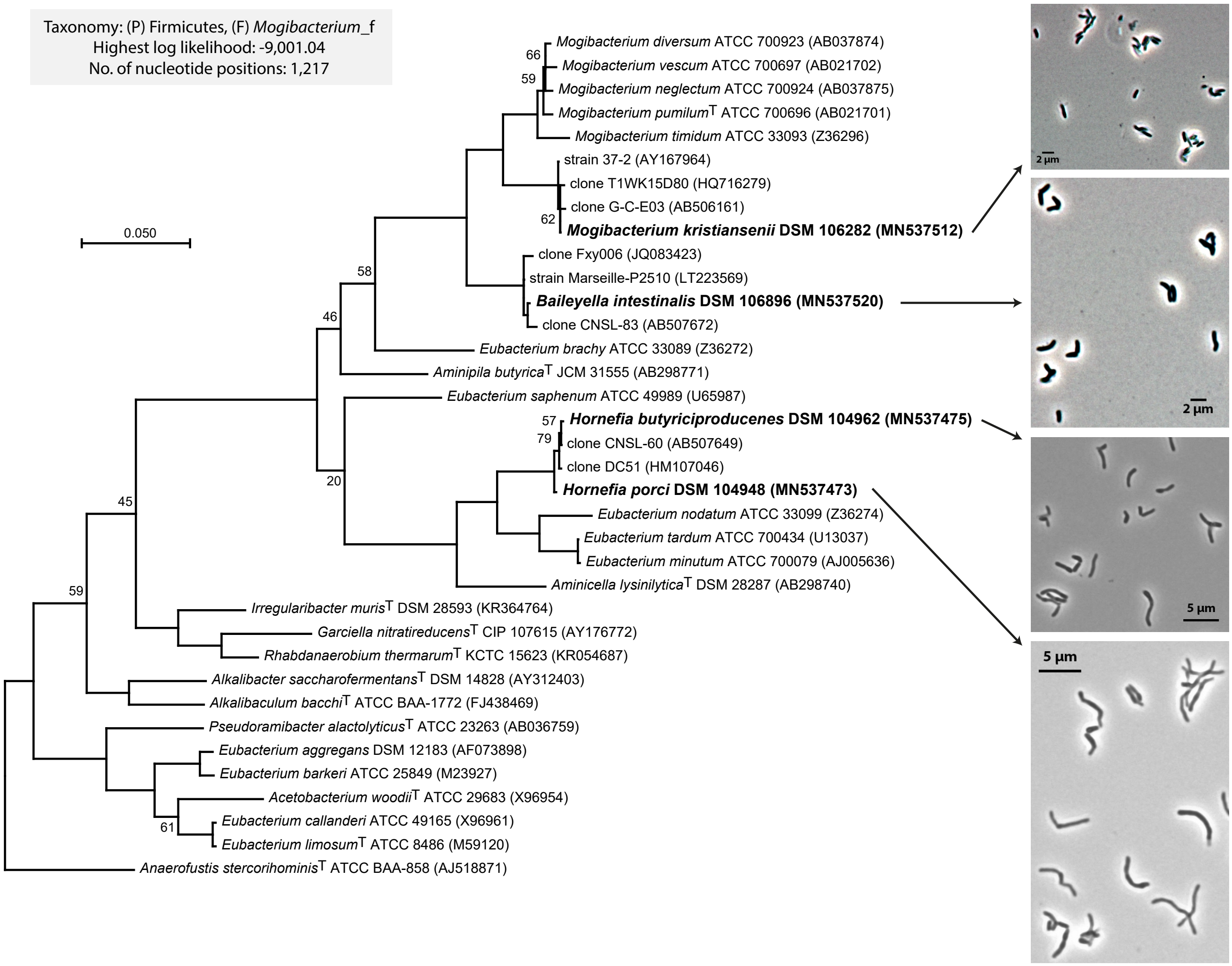

Suppl. Fig. 4p 16S rRNA gene-based phylogenetic tree of *Anaerovibrio slackiae* sp. nov. and *Selenomonas montiformis* sp. nov.

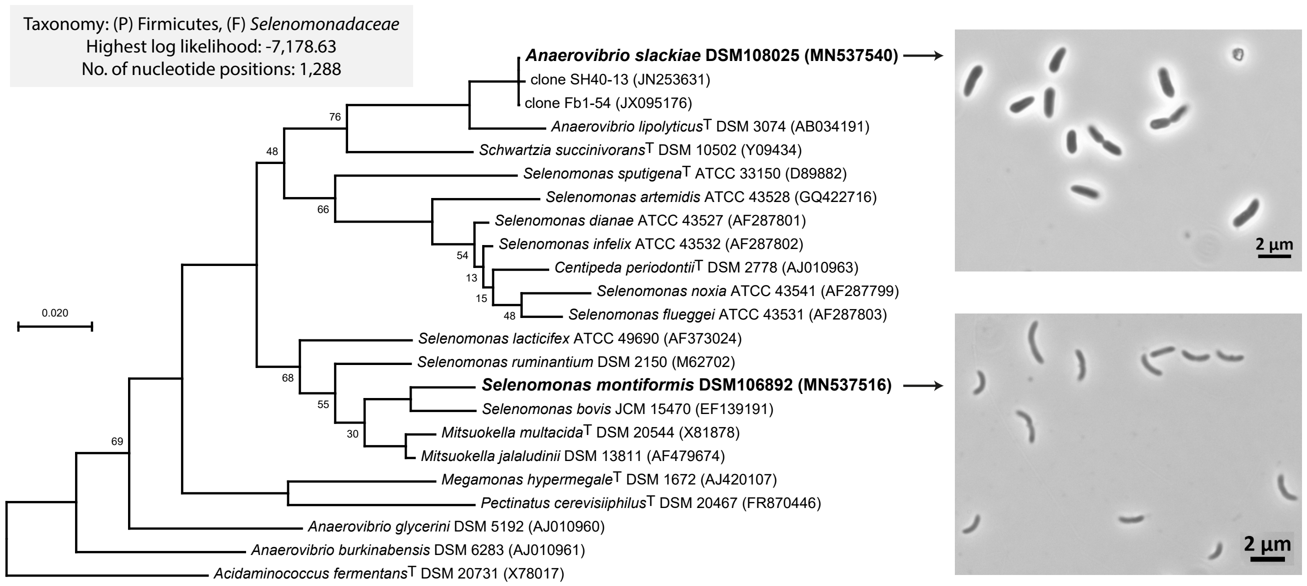

Suppl. Fig. 4q 16S rRNA gene-based phylogenetic tree of *Peptoniphilus porci* sp. nov., *Peptostreptococcus porci* sp. nov., and *Anaerococcus porci* sp. nov.

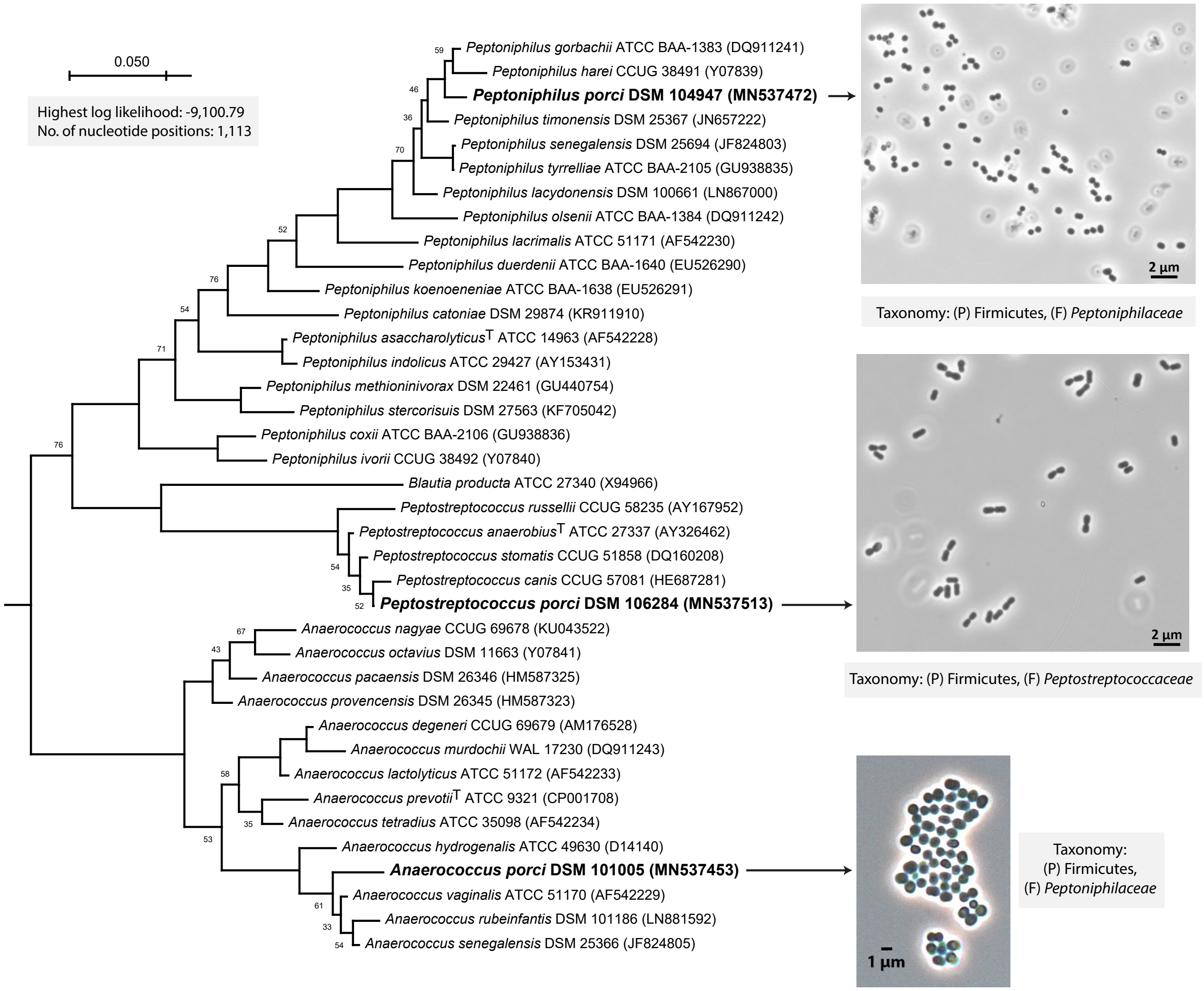

Suppl. Fig. 4r 16S rRNA gene-based phylogenetic tree of *Victivalis lenta* sp. nov.

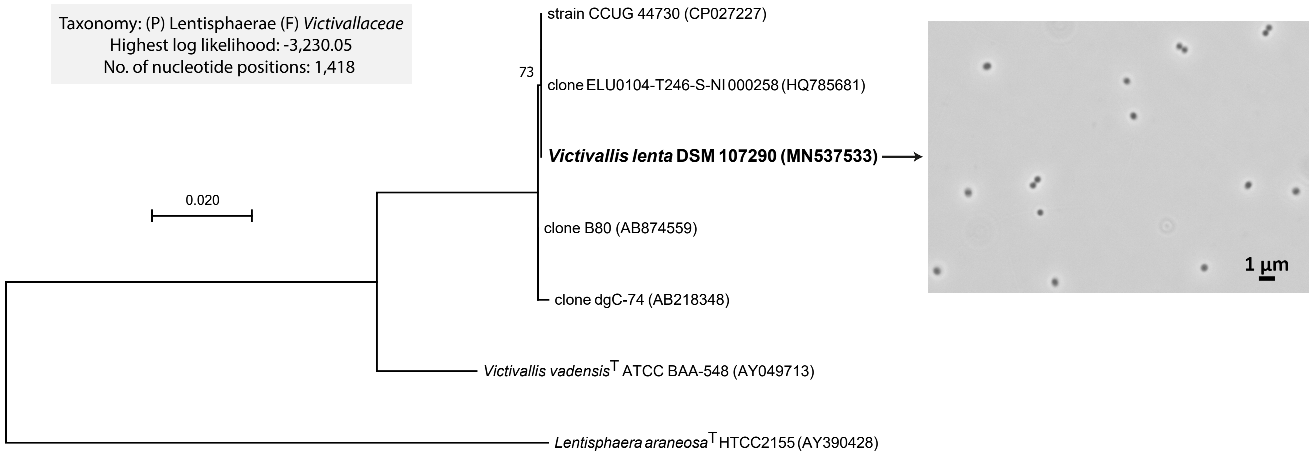

Suppl. Fig. 4s 16S rRNA-based phylogenetic tree of *Desulfovibrio porci* sp. nov.

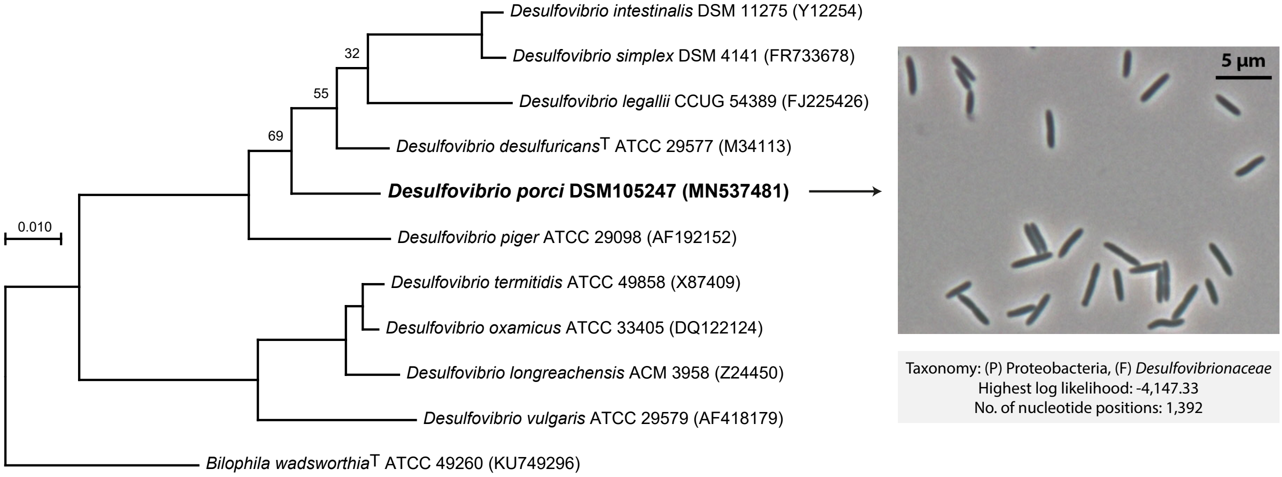

Suppl. Fig. 4t 16S rRNA gene-based phylogenetic tree of *Bullifex porci* gen. nov., sp. nov.

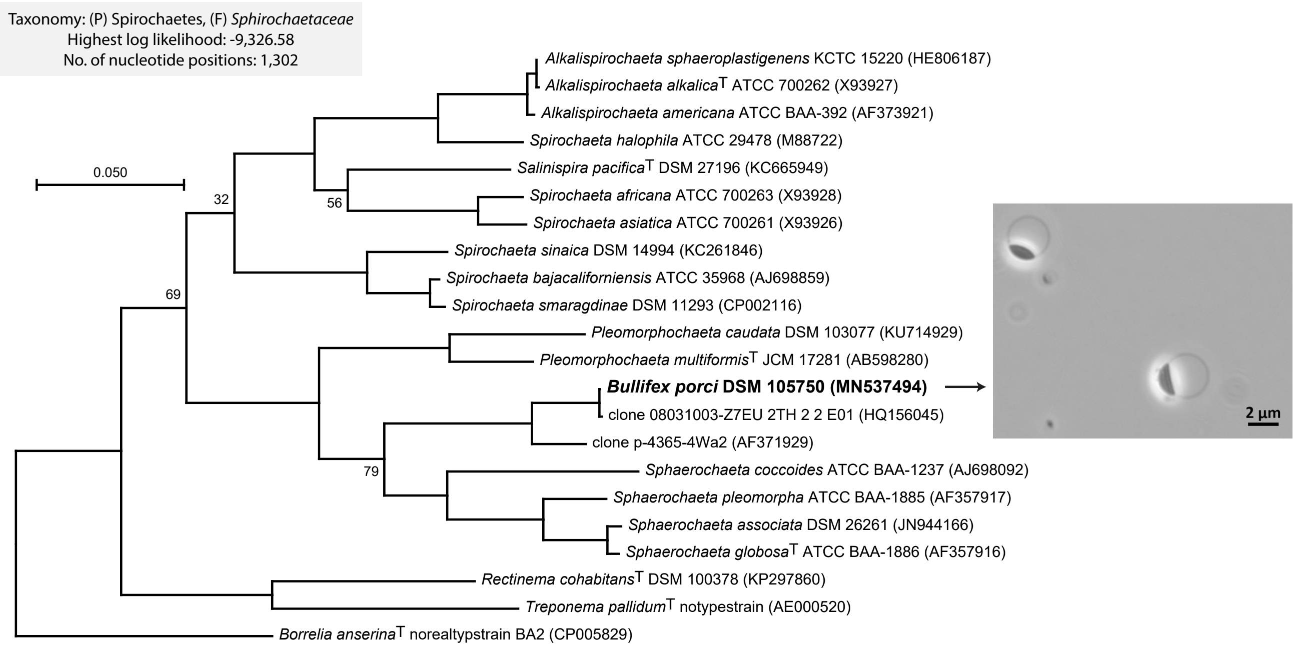

Suppl. Fig. 4u 16S rRNA gene-based phylogenetic tree of *Pyramidobacter porci* gen nov., sp. nov.

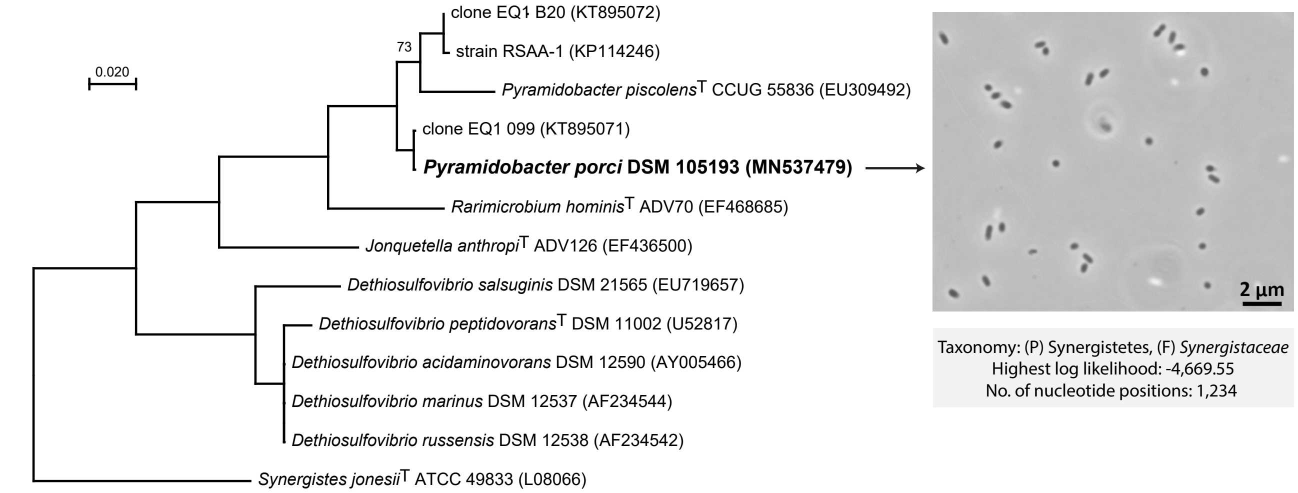

Suppl. Fig. 4v 16S rRNA gene-based phylogenetic tree of *Hallerella porci* gen nov., sp. nov. and *Hallerella succinigenes* sp. nov.

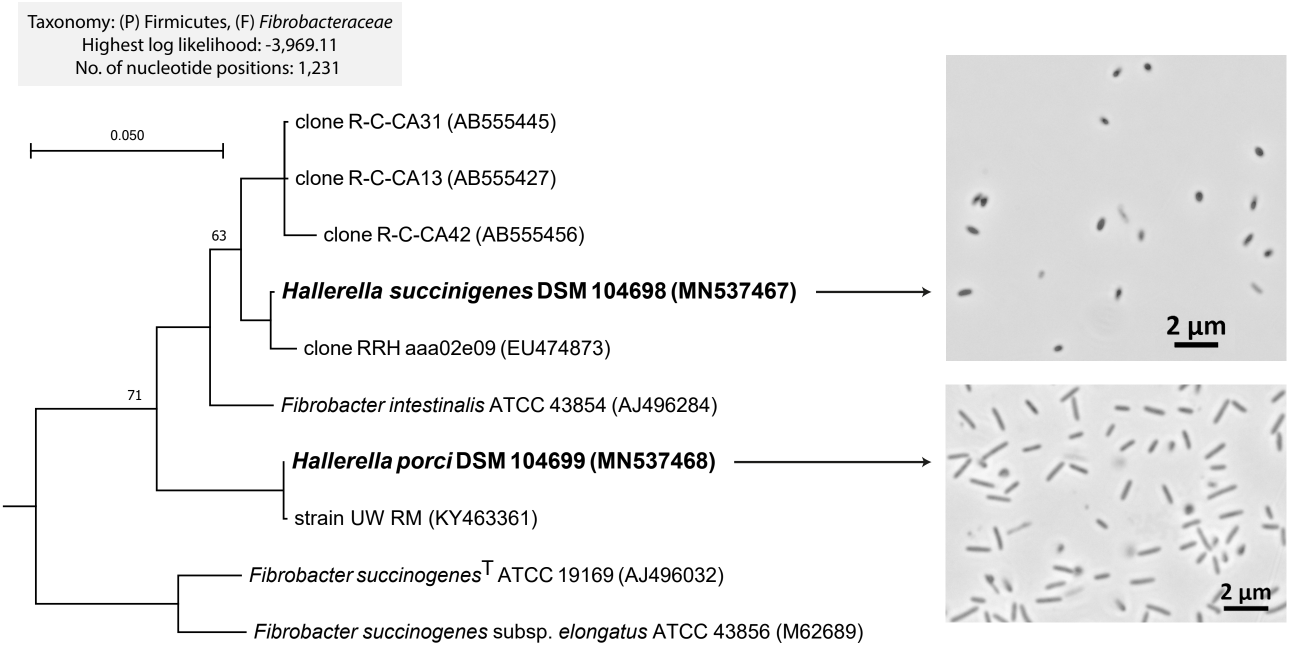

Electron Microscopy Facility of RWTH Aachen University

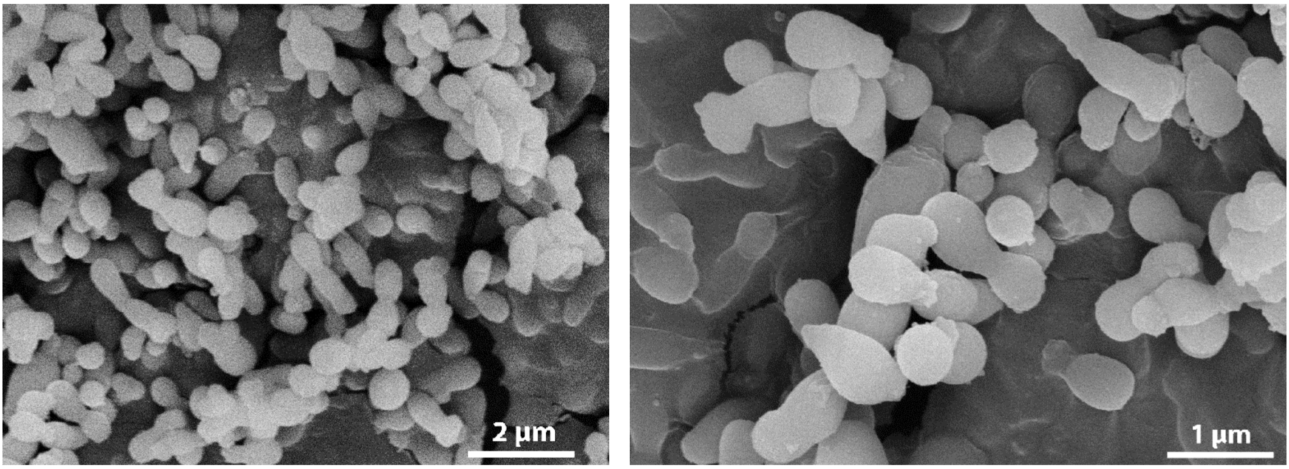

Supplementary Fig. 5a / Scanning electron micrographs of *Pseudoramibacter porci* DSM 106894ᵀ.

Electron Microscopy Center at Wageningen University

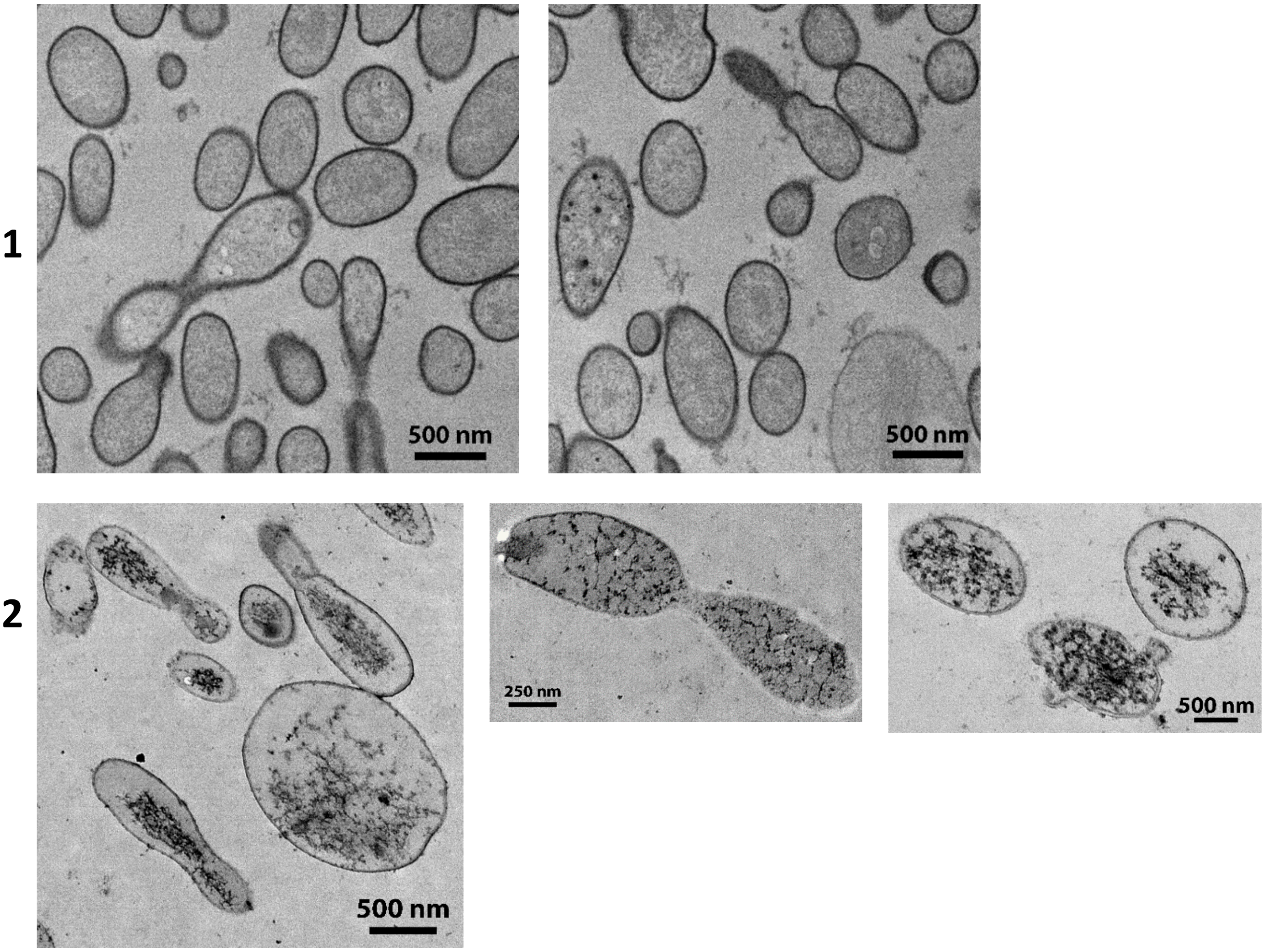

Supplementary Fig. 5b / Transmission electron micrographs of *Pseudoramibacter porci* DSM 106894ᵀ
(1) Cells fixed with 2% (v/v) formaldehyde and 2.5% (v/v) glutaraldehyde (protocol 1 with ruthenium red).
(2) Cells were processed by high pressure freezing (protocol 2; see methods section).

Electron Microscopy Facility of RWTH Aachen University

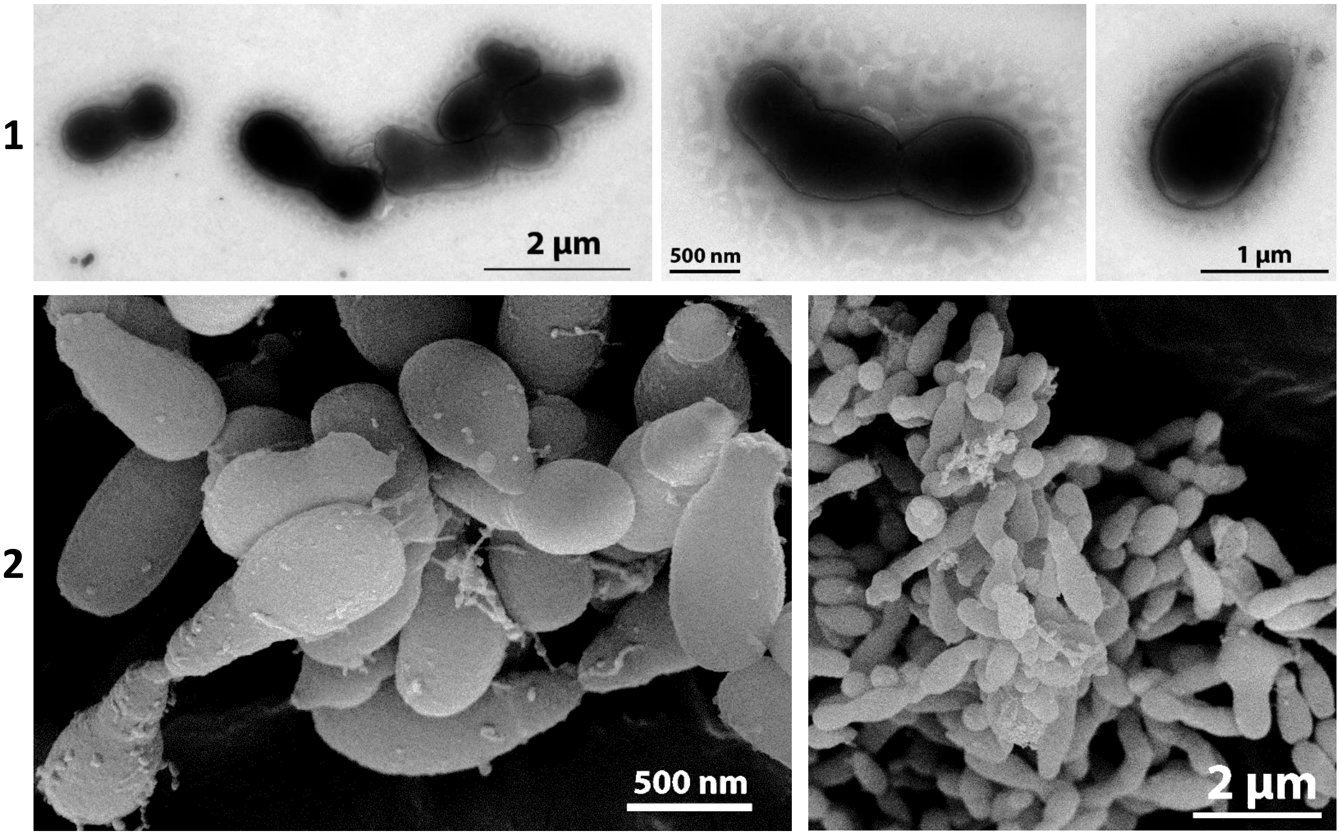

Supplementary Fig. 6a / Electron micrographs of *Stecheria intestinalis* DSM 109718^{T}.
(1) Negative staining of cells observed by TEM indicating the presence of an extra-cellular matrix surrounding cells.
(2) Cells imaged by means of scanning electron microscopy after fixation with 3% (v/v) glutaraldehyde.

Electron Microscopy Center at Wageningen University

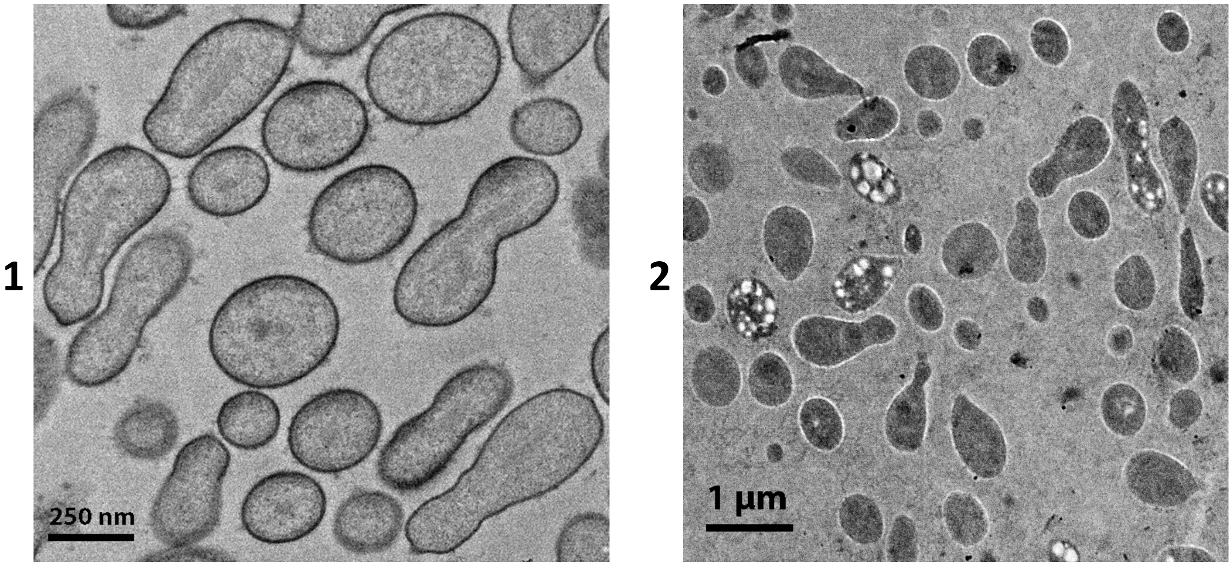

Supplementary Fig. 6b / Transmission electron micrographs of *Stecheria intestinalis* DSM 109718^{T}.
(1) Cells fixed with 2% (v/v) formaldehyde and 2.5% (v/v) glutaraldehyde (protocol 1 with ruthenium red).
(2) Cells were processed by high pressure freezing (protocol 2; see methods section).

Electron Microscopy Facility of RWTH Aachen University

1

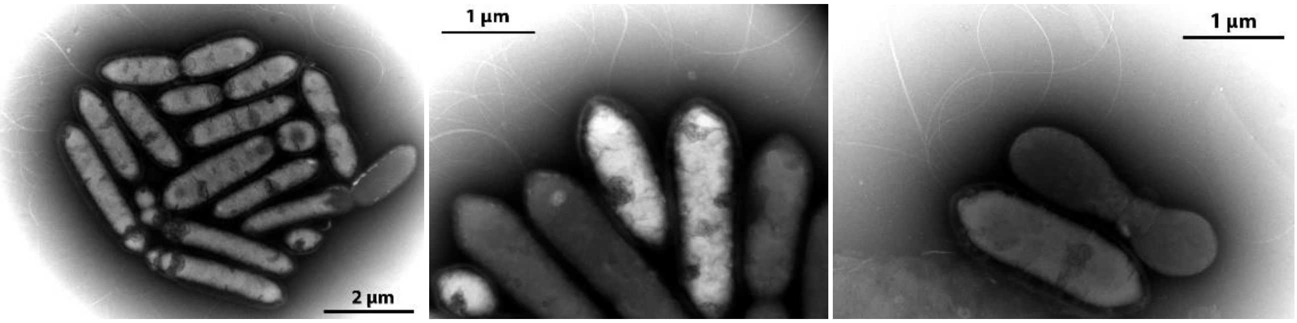

2

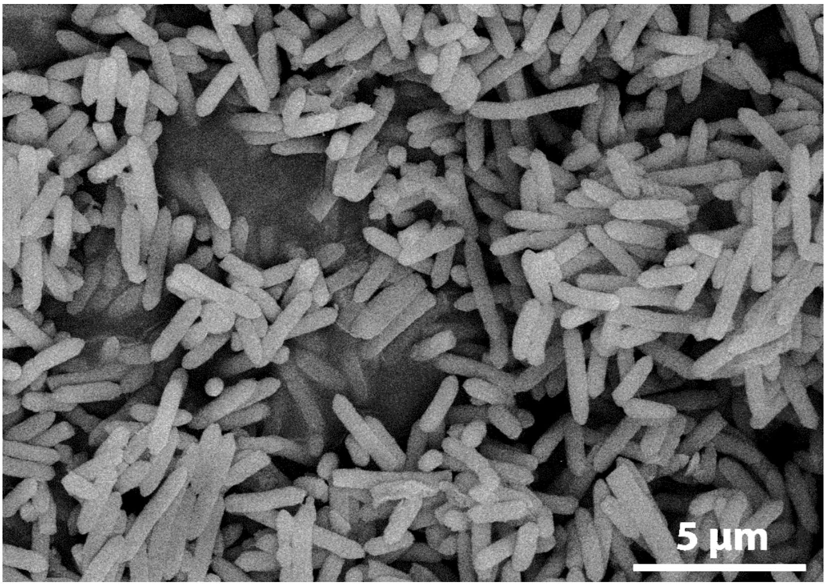

Supplementary Fig. 7a / Electron micrographs of *Tissierella pigra* DSM 109718^{T} performed at RWTH university.

(1) Negative staining of cells observed by TEM indicating the presence of an extra-cellular matrix surrounding cells.

(2) Cells imaged by means of scanning electron microscopy after fixation with 3% (v/v) glutaraldehyde.

Electron Microscopy Center at Wageningen University

1

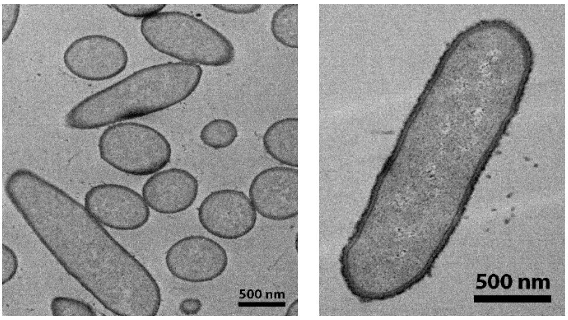

2

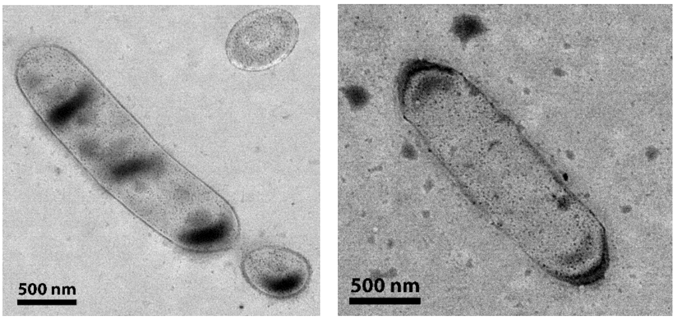

Supplementary Fig. 7b / Transmission electron micrographs of *Tissierella pigra* DSM 109718^{T}.
(1) Cells fixed with 2% (v/v) formaldehyde and 2.5% (v/v) glutaraldehyde (protocol 1 with ruthenium red).
(2) Cells were processed by high pressure freezing (protocol 2; see methods section).

1

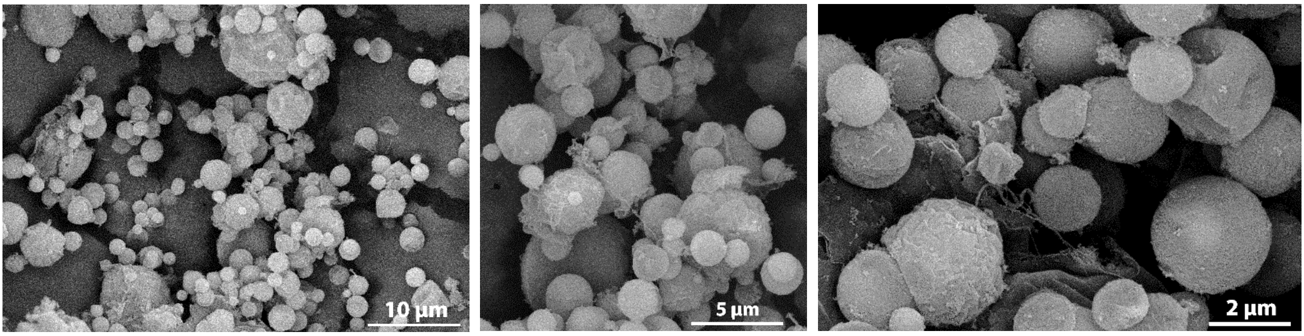

2a

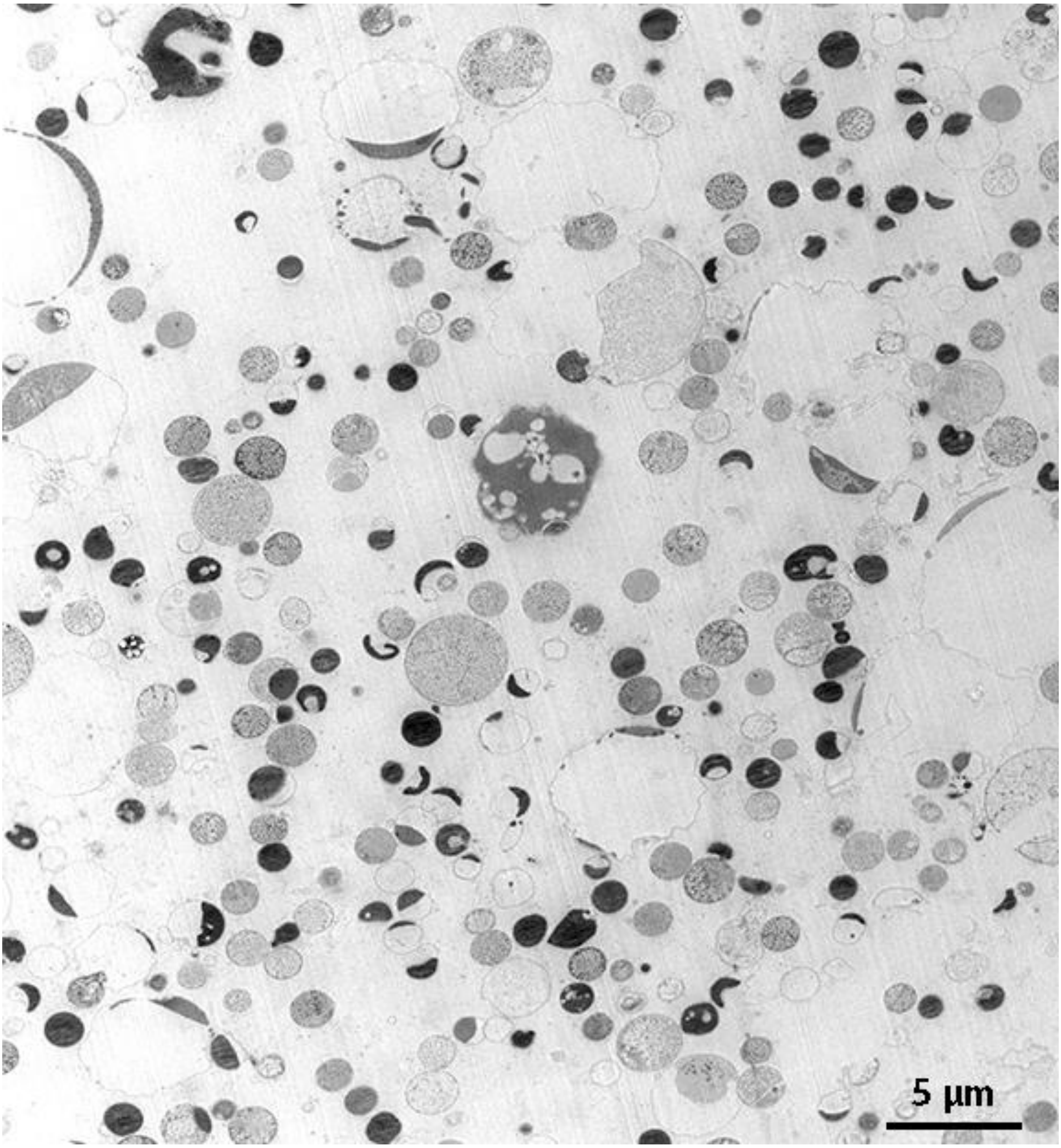

Supplementary Fig. 8a / Electron micrographs of *Bullifex porci* DSM 105750^T performed at RWTH university.
(1) Scanning electron micrographs. **(2a)** Transmission electron micrographs (1.5%-glutaraldehyde fixation, embedding in 1% agarose, high-pressure freezing, ethanol-dehydration).

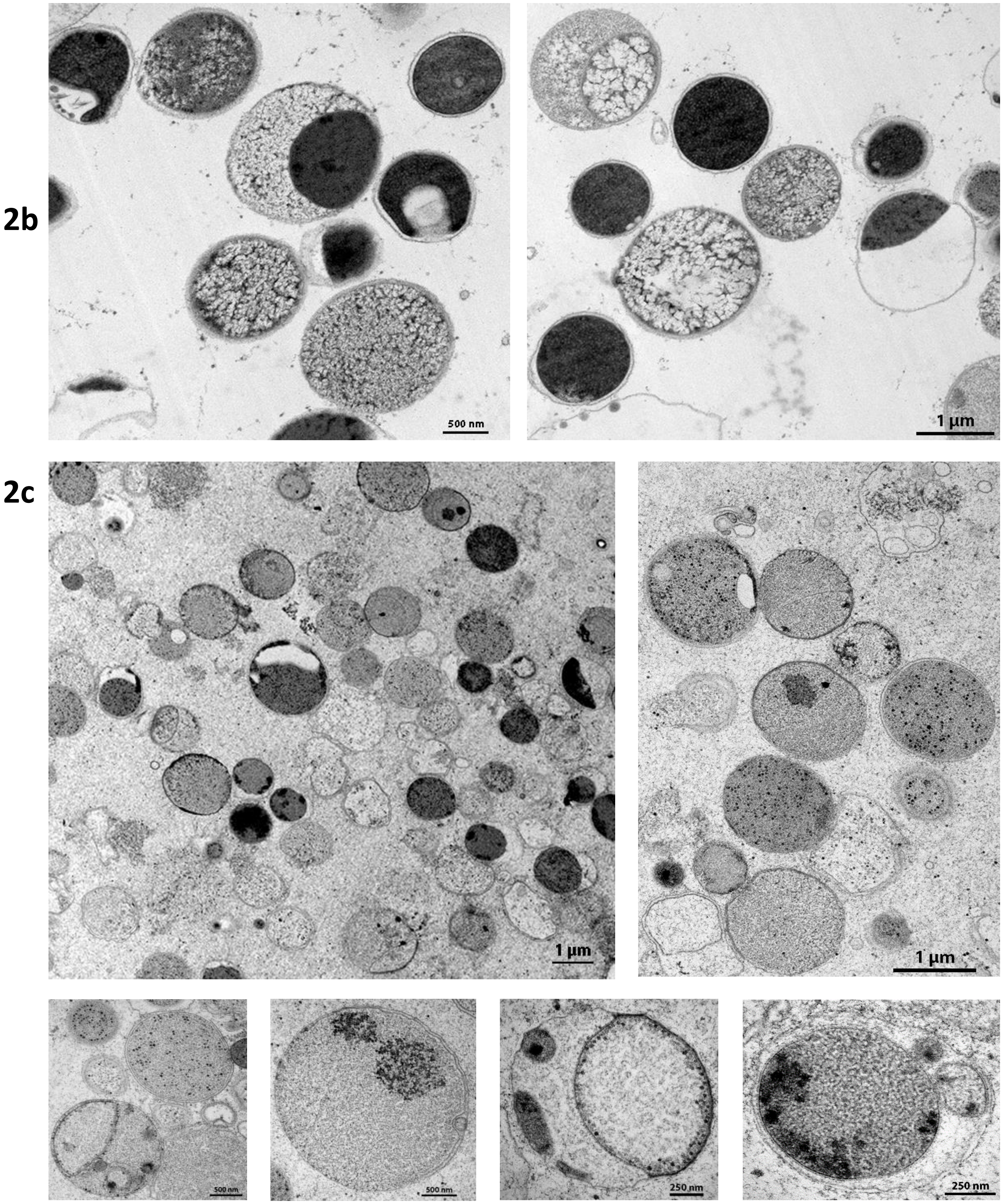

Supplementary Fig. 8a / Electron micrographs of *Bullifex porci* DSM 105750^T performed at RWTH university.
Transmission electron micrographs: **(2b)** 1.5%-glutaraldehyde fixation, embedding in 1% agarose, high-pressure freezing, ethanol-dehydration; **(2c)** Same with 0.2%-glutaraldehyde fixation.

2d

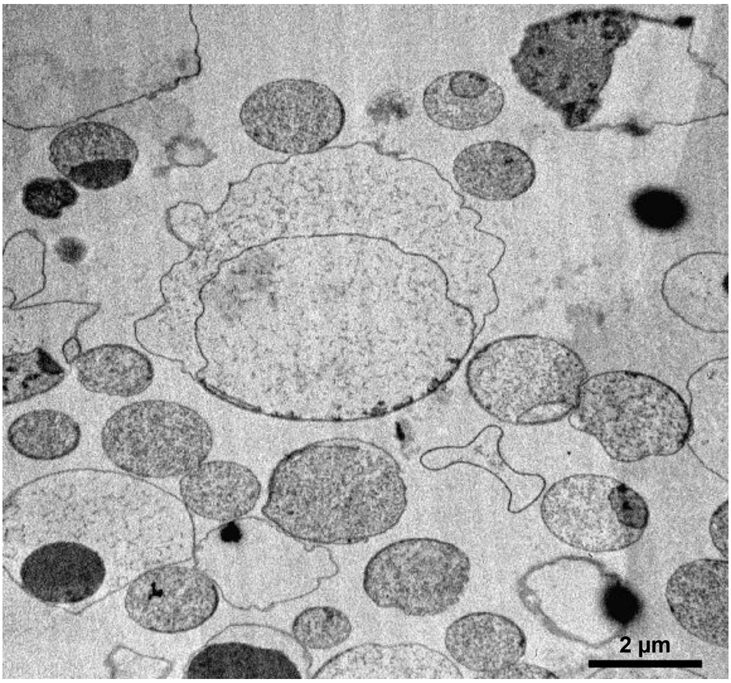

2e

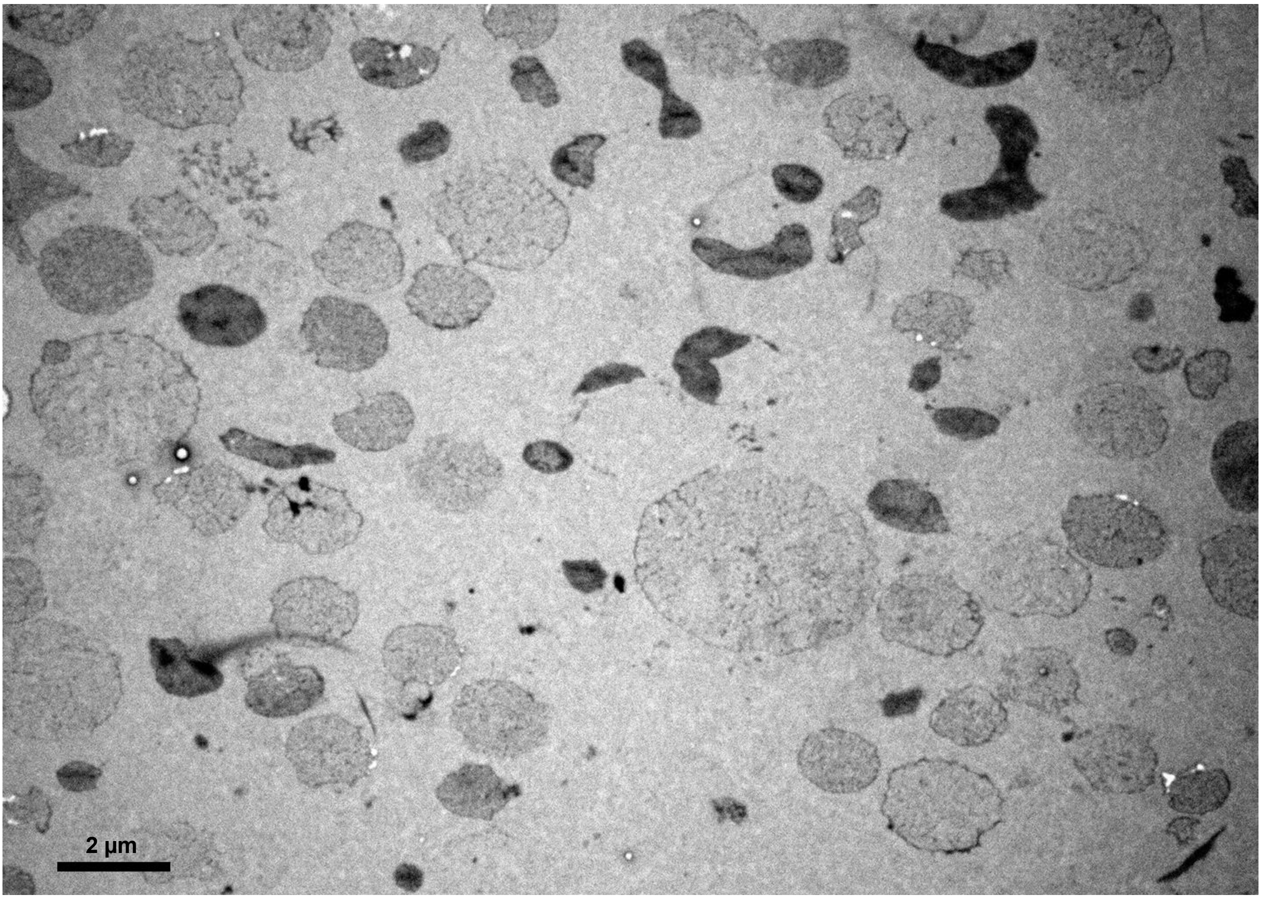

Supplementary Fig. 8b / Electron micrographs of *Bullifex porci* DSM 105750^{T} performed at Wageningen university.
(2d) Transmission electron micrographs of cells fixed with 2% (v/v) formaldehyde and 2.5% (v/v) glutaraldehyde (protocol 1).
(2e) Transmission electron micrographs of cells processed by high pressure freezing followed by freeze substitution (protocol 2).

2f

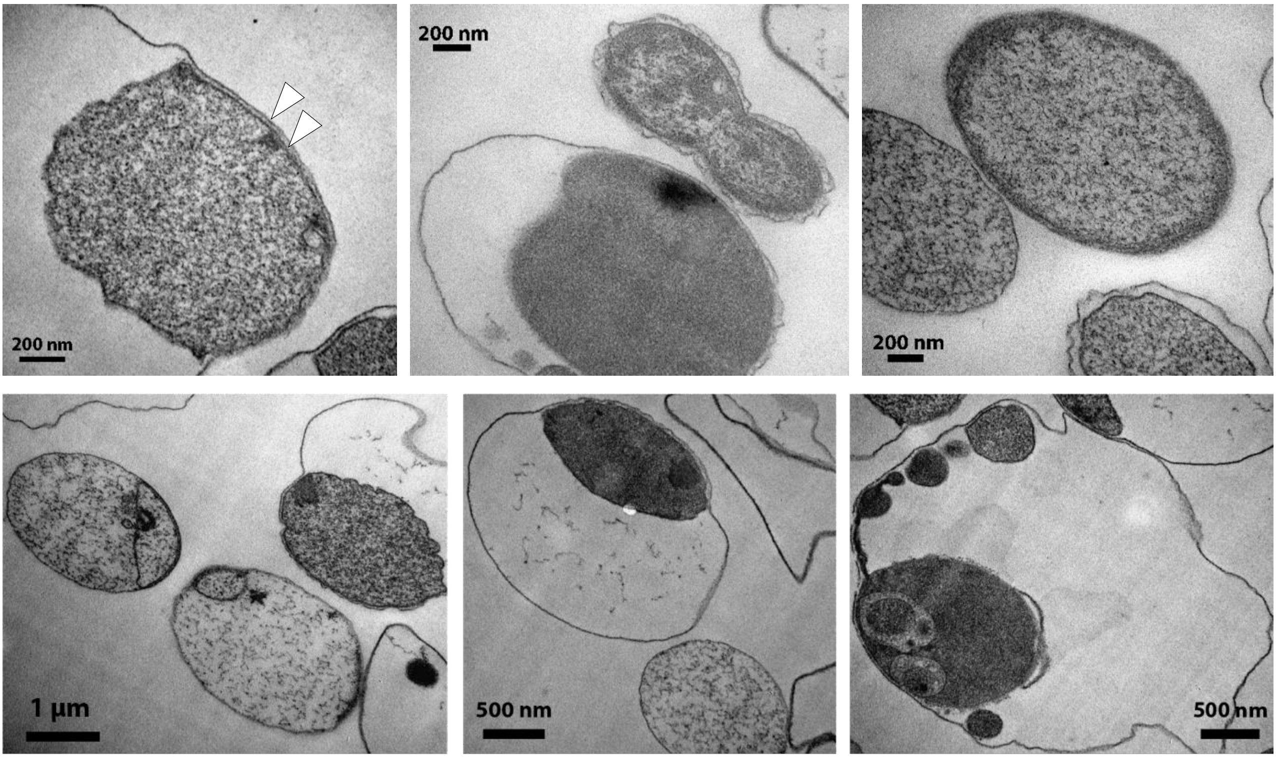

2g

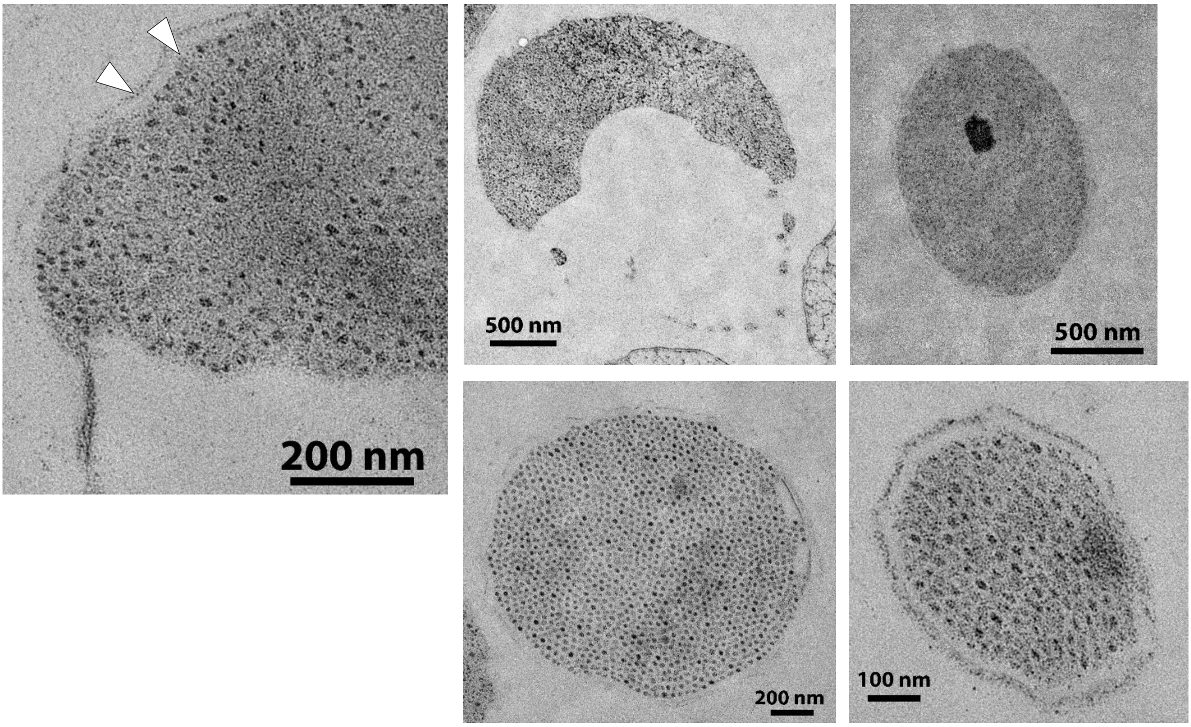

Supplementary Fig. 8b / Electron micrographs of *Bullifex porci* DSM 105750^{T} performed at Wageningen university.
(2f) Transmission electron micrographs of cells fixed with 2% (v/v) formaldehyde and 2.5% (v/v) glutaraldehyde (protocol 1).
(2g) Transmission electron micrographs of cells processed by high pressure freezing followed by freeze substitution (protocol 2; see methods section). White arrows point at intact membranes, indicating the presence of viable cells

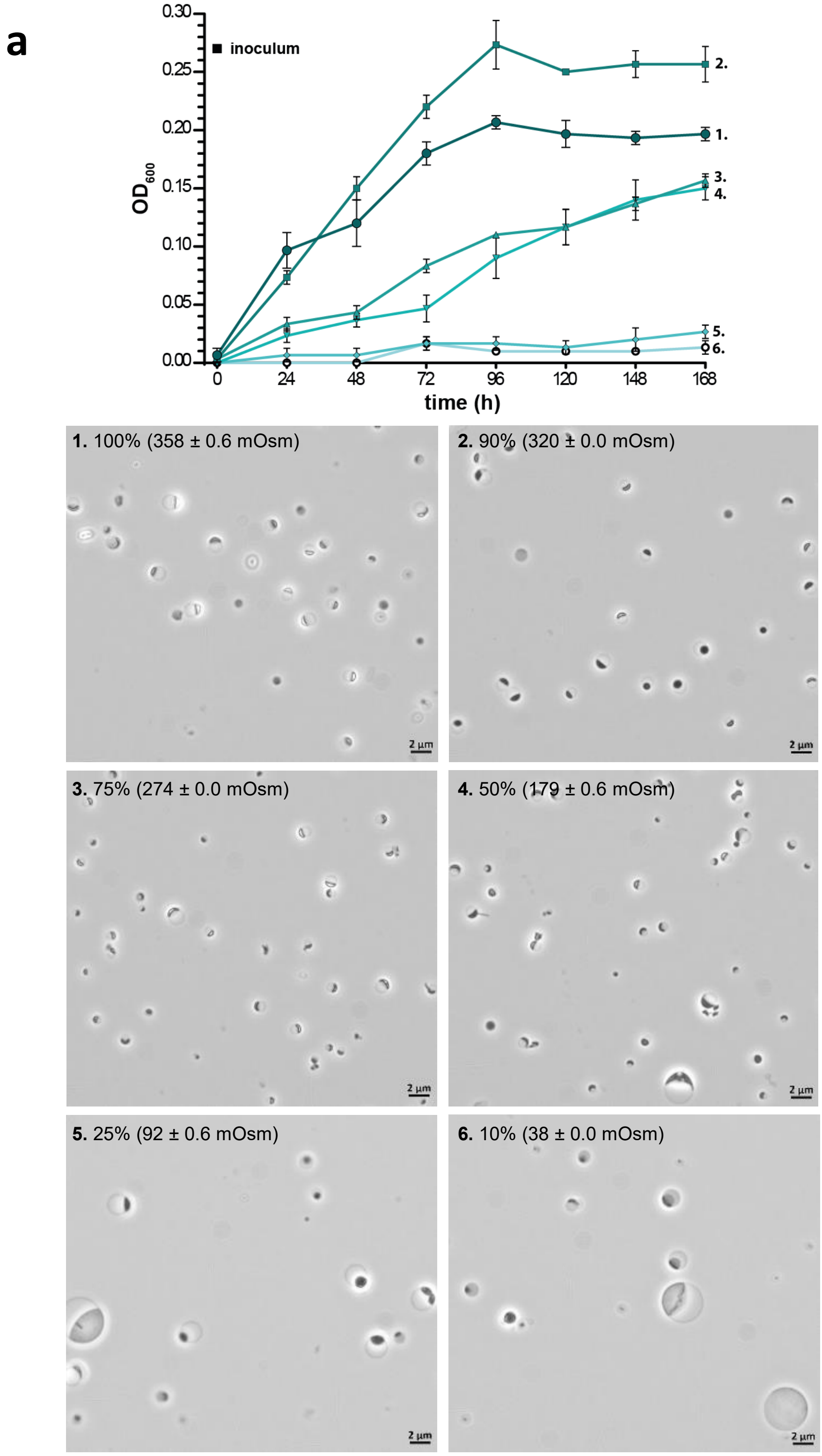

Supplementary Fig. 9a / Impact of medium dilution on the growth and cell morphology of *Bullifex porci* DSM 105750^{T}. The strain was grown for 7 days (168 h) at 37 °C with constant shaking (220 rpm) under anaerobic conditions in Hungate tubes containing 10 ml of media. A gradient of medium dilutions (100, 90, 75, 50, 25, and 10%, as indicated in the images) was obtained by mixing the basal medium (BHI broth supplemented with 10% rumen fluid) with autoclaved and gassed distilled water in appropriate volume ratios. Triplicate cultures were tested for each condition. Data is represented as mean ± SD. The osmolarity of all media was measured using an OSMO Station OM-6050 (Arkray, Kyoto, Japan). Growth was followed overtime by measuring the $OD_{600\ nm}$ directly within the Hungate tubes using a CO8000 Cell Density meter. After one week of growth, cells were centrifuged (3,500 x g, 10 min) and re-suspended in a lower volume of the corresponding growth medium to increase cell density. Cell morphology was observed by phase contrast microscopy as in Fig. 2.

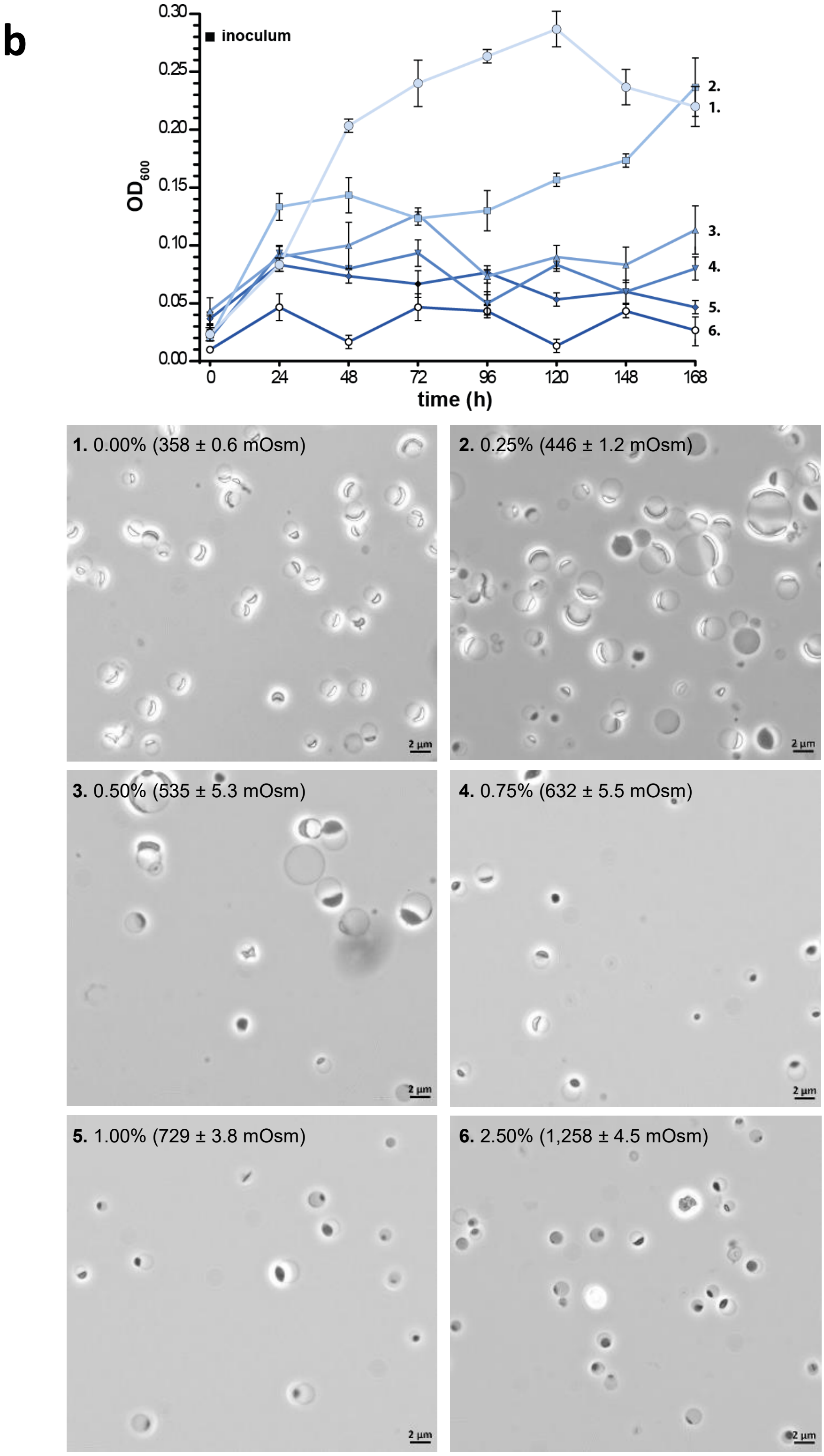

Supplementary Fig. 9b / Impact of NaCl supplementation on the growth and cell morphology of *Bullifex porci* DSM 105750^{T}. The strain was grown for 7 days (168 h) at 37 °C with constant shaking (220 rpm) under anaerobic conditions in Hungate tubes containing 10 ml of media. For increasing osmolarity, the basal medium (BHI broth with 10% rumen fluid) was supplemented with the appropriate amount of NaCl (0, 0.25, 0.50, 0.75, 1.00, and 2.50% (w/v) as indicated in the microscopy images) prior to gassing and autoclaving. Triplicate cultures were tested for each condition. Data is represented as mean ± SD. The osmolarity of all media was measured using an OSMO Station OM-6050 (Arkray, Kyoto, Japan). Growth was followed overtime by measuring the $OD_{600\ nm}$ directly within the Hungate tubes using a CO8000 Cell Density meter. After one week of growth, cells were centrifuged (3,500 x g, 10 min) and re-suspended in a lower volume of the corresponding growth medium to increase cell density. Cell morphology was observed by phase contrast microscopy as in Fig. 2.

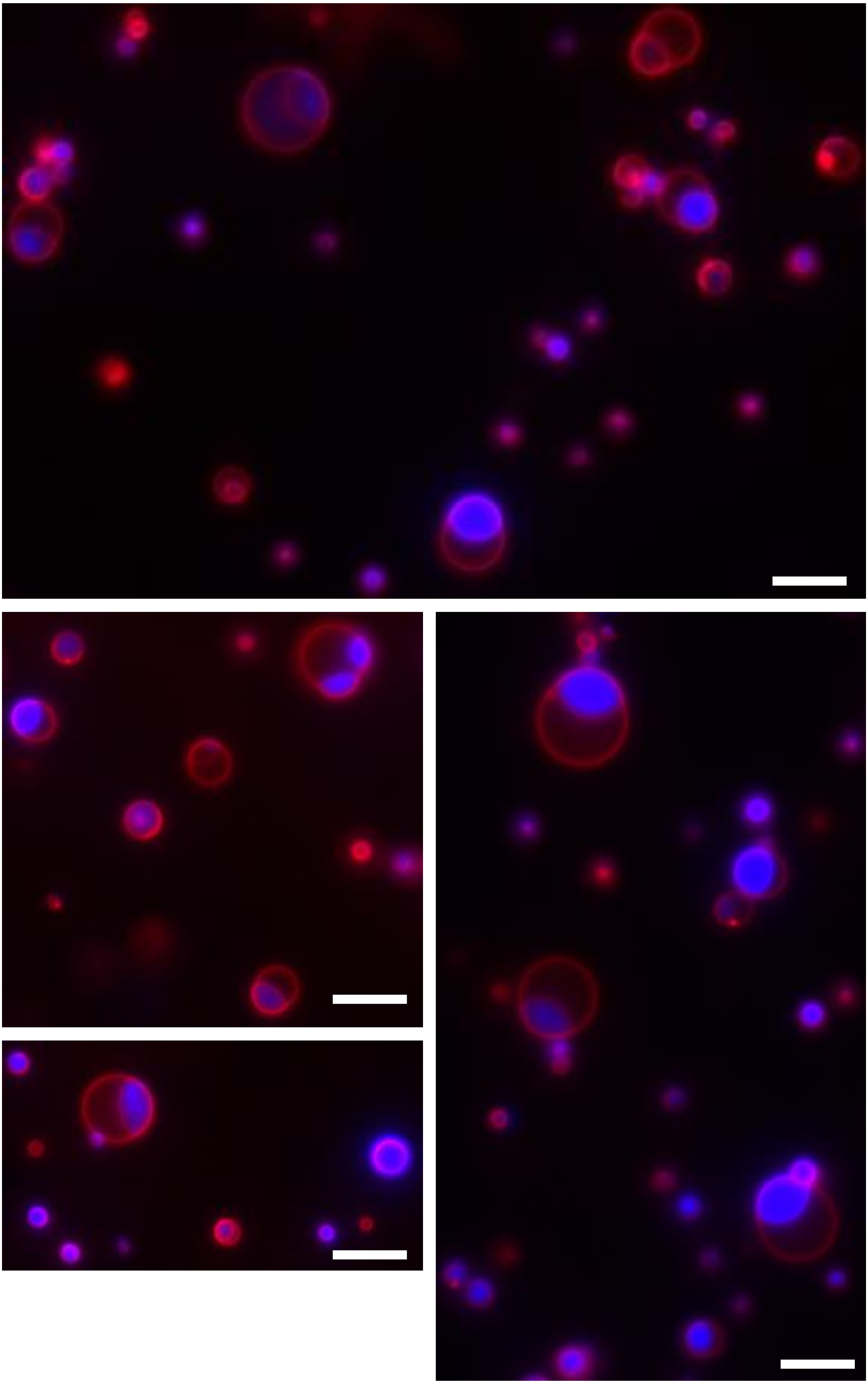

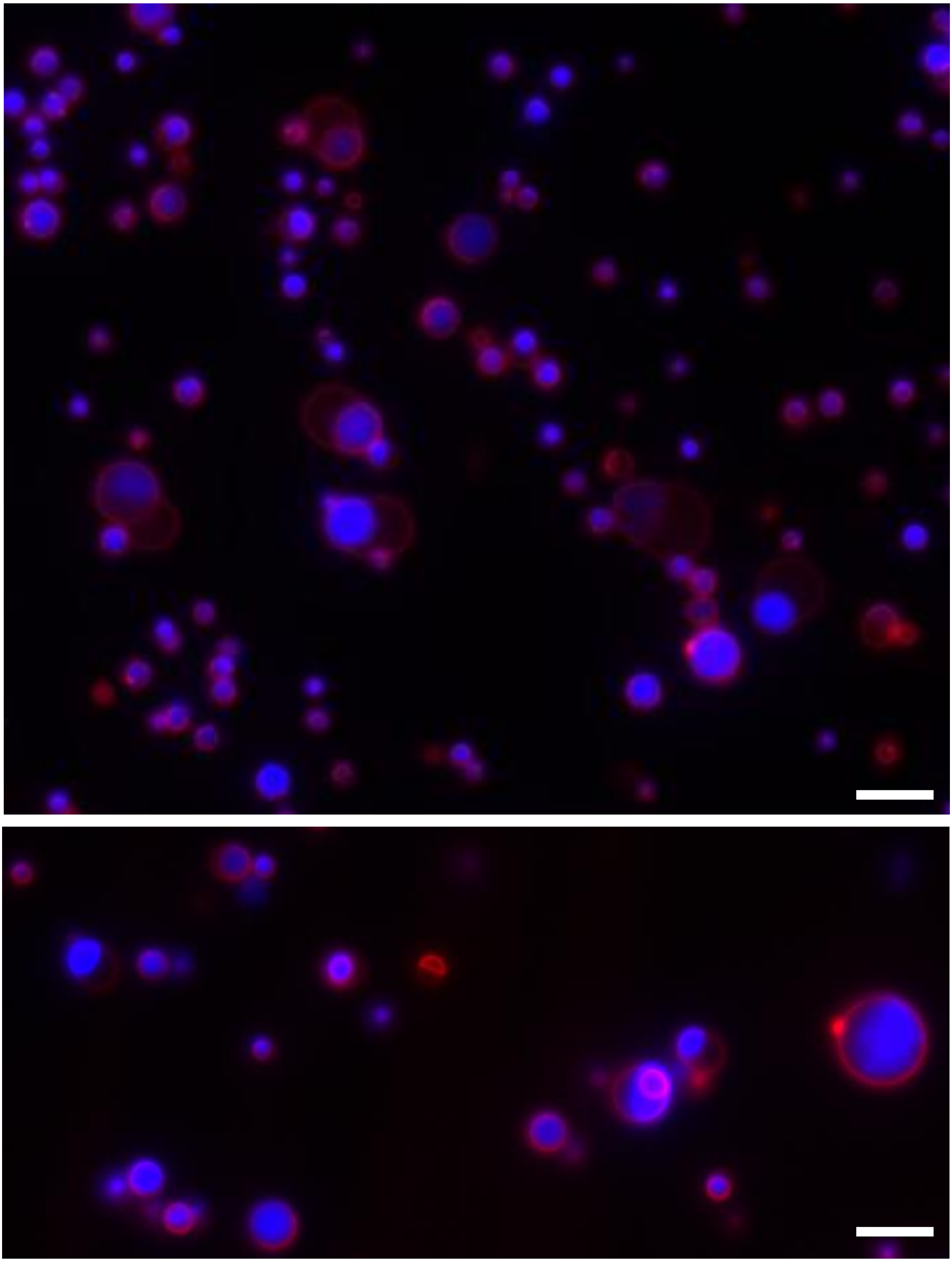

Supplementary Fig. 10 / Fluorescence microscopy images of *Bullifex porci* DSM 105750^{T}.
The strain was grown for 4 days in BHI medium supplemented rumen fluid, cysteine and DTT under anaerobic conditions. Cells were stained with FM4-64 (1 µg/ml; red; membrane) and DAPI (2 µg/ml; blue; DNA) after a wash in PBS (2 min, 5,000 rpm). They were then visualized using an Axio Imager.Z2 microscope (Zeiss, Jena, Germany) equipped with a Plan-Apochromat x 63 phase contrast objective lens, appropriate filter sets, and an ORCA-Flash 4.0 LT digital CMOS camera (Hamamatsu Photonics, Shizuoka, Japan) using the Zeiss Zen Blue software. Brightness and contrast level of the images were adjusted using ImageJ. The bar in the images represents 5 µm.

a

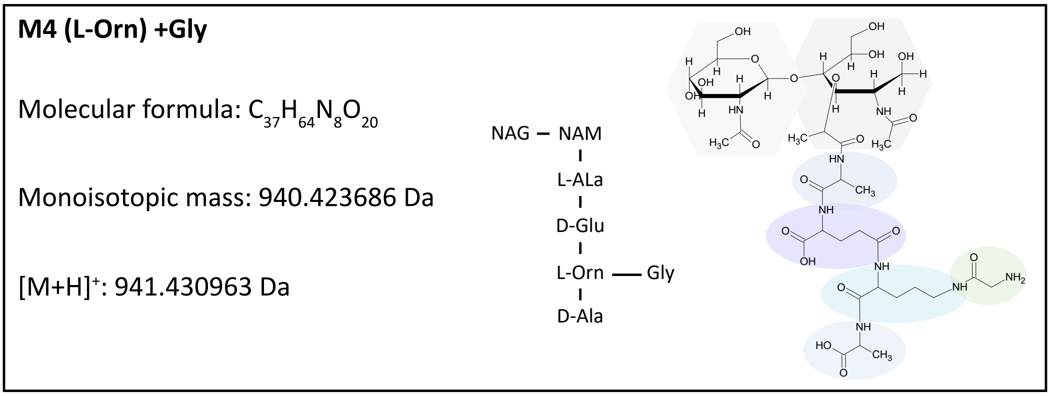

b

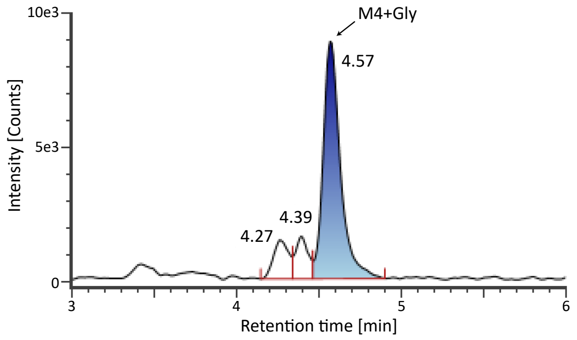

c

Ion	Theoretical m/z	Measured m/z	Error
M+H	941.430962	941.42868	+0.00228
M+Na	963.412904	963.40803	+0.00487
M+2H	471.219119	471.21863	+0.00049
M+2Na	493.201061	493.20106	-0.000001

d

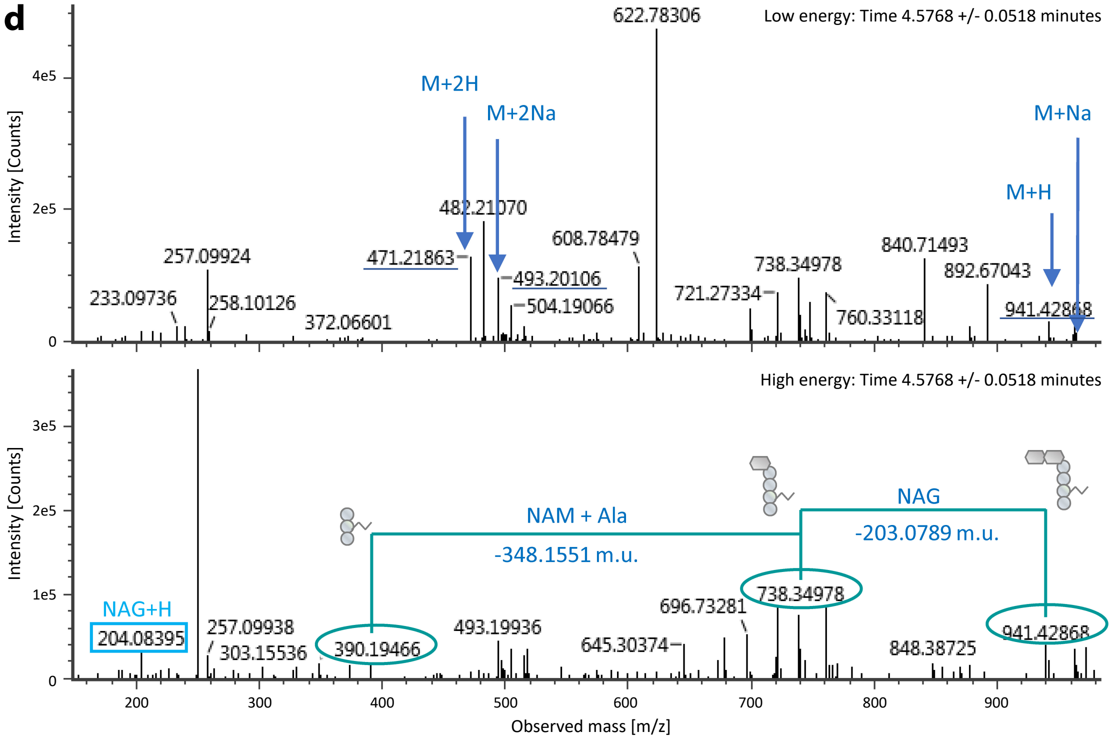

Supplementary Fig. 11 / Detected peptidoglycan in *Bullifex porci*.
Visible and total ion chromatograms obtained from peptidoglycan preparations of *B. porci* did not show any obvious muropeptide profile, pointing at low amounts of peptidoglycan isolated from liters of culture. Use of a UNIFI compound library allowed the detection of muropeptides with m/z value and fragmentation pattern corresponding to a murotetrapeptide with L-Orn linked to a single glycine, as reported previously for phylogenetically related bacteria[3-4]
a, Chemical structure proposed for the muropeptides detected in the isolated peptidoglycan. NAG, *N*-acetylglucosamine; NAM, *N*-acetylmuramic acid. **b**, Extracted ion chromatogram of the detected M4 (L-Orn) + Gly ions. **c**, Theoretical and observed m/z values for the different ion adducts of the M4 (L-Orn) + Gly muropeptide detected. **d**, Low- (top panel) high- (bottom) energy mass spectrum obtained during the fragmentation of the molecular ion of M4 (L-Orn) + Gly molecule (m/z value 941.42 Da) indicating the different adduct ions detected (top) and the fragmentation pattern of the molecule (bottom)

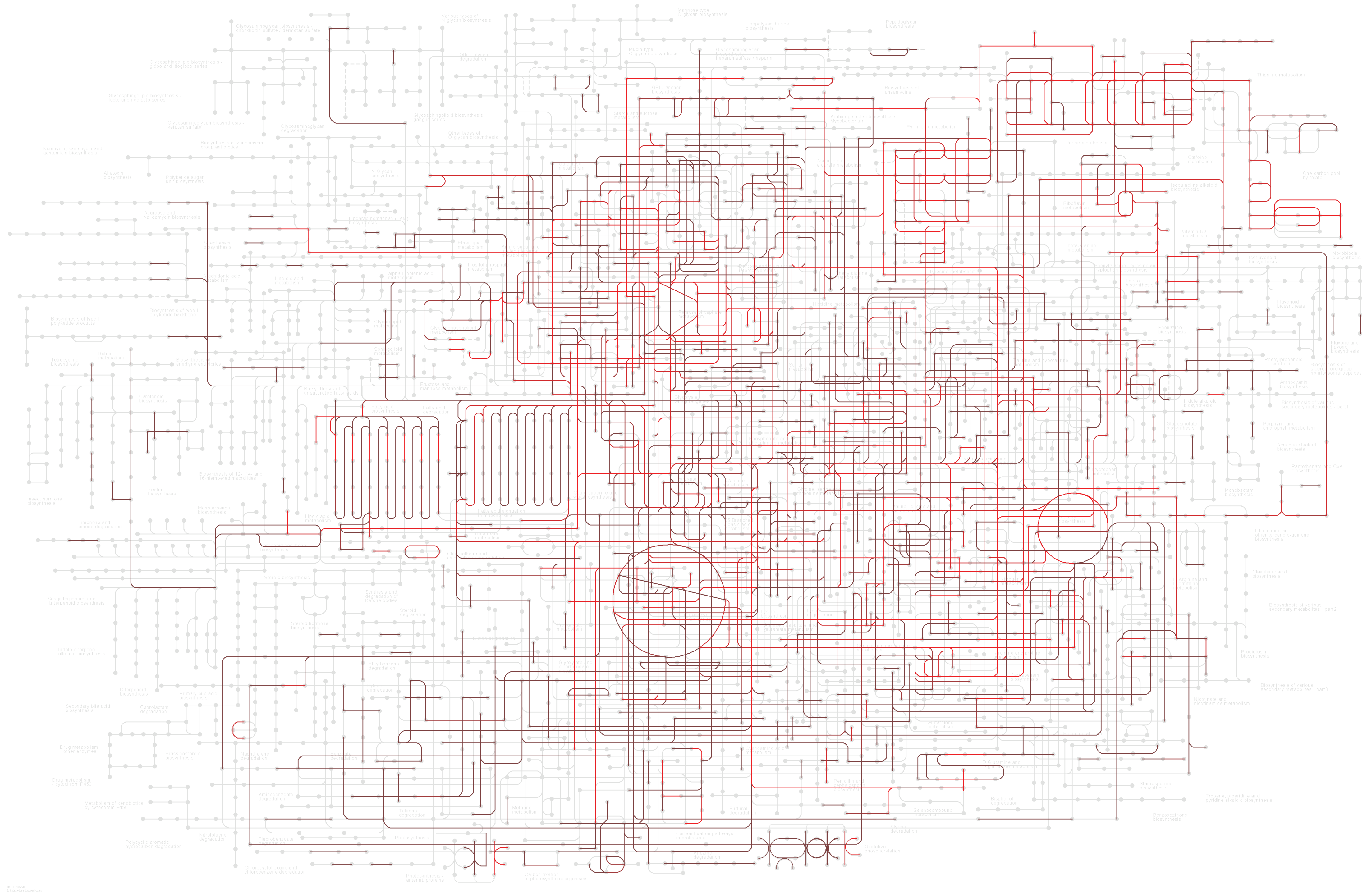

Supplemental Figure 12 / KEGG pathway coverage by the PiBAC collection. KEGG orthologs (KO) were searched for within each of the 117 isolate genomes and the prevalence of each KO visualised over the Metabolic pathways map (map01100). Prevalence is represented via KO colour from dark red to light red.

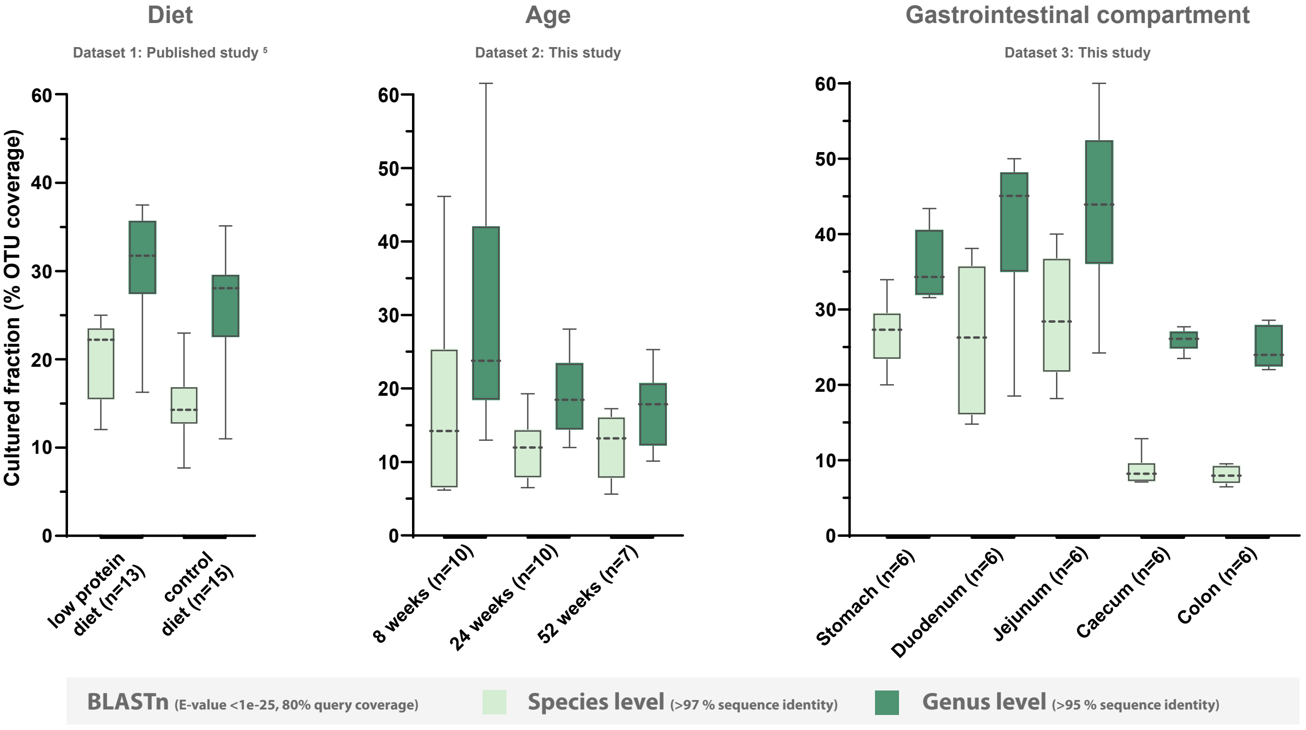

Supplementary Fig. 13 / Cultured fractions as determined using study-specific 16S rRNA gene amplicon data. To assess the coverage of sequence-based diversity by all PiBAC isolates in relation to parameters such as diet, age, and gut locations, we processed three datasets:

Dataset 1: Published data[5] investigating the impact of dietary protein content on the fecal microbiota of castrated male Duroc pigs at the age of 165 days;
Datasets 2: Stool samples from ten German landrace pigs from the animal facility of Thalhausen (TU Munich, Germany) at the age of 8, 24, and 52 weeks;
Dataset 3: Samples from six wildtype pigs from the MIDY biobank,[6] including five locations within the gastro-intestinal tract.

For study 2 and 3, samples were processed as previously described (see also method section).[7] All sequencing data were processed using IMNGS[8] and Rhea.[9]
Cultured fractions were determined by searching for matches between the amplicon sequences of all study-specific OTUs (those occurring at a relative abundance of ≥0.25% in at least one sample within the respective dataset) and 16S rRNA gene sequences of the isolates using blastn (E-value <1e-25, 80% query coverage) at two different sequence identity thresholds: 97% (as proxy for species level) and 95% (genus level). Data are shown in a box plots, center line: median, bounds of box: quartile, whiskers: Tukey.

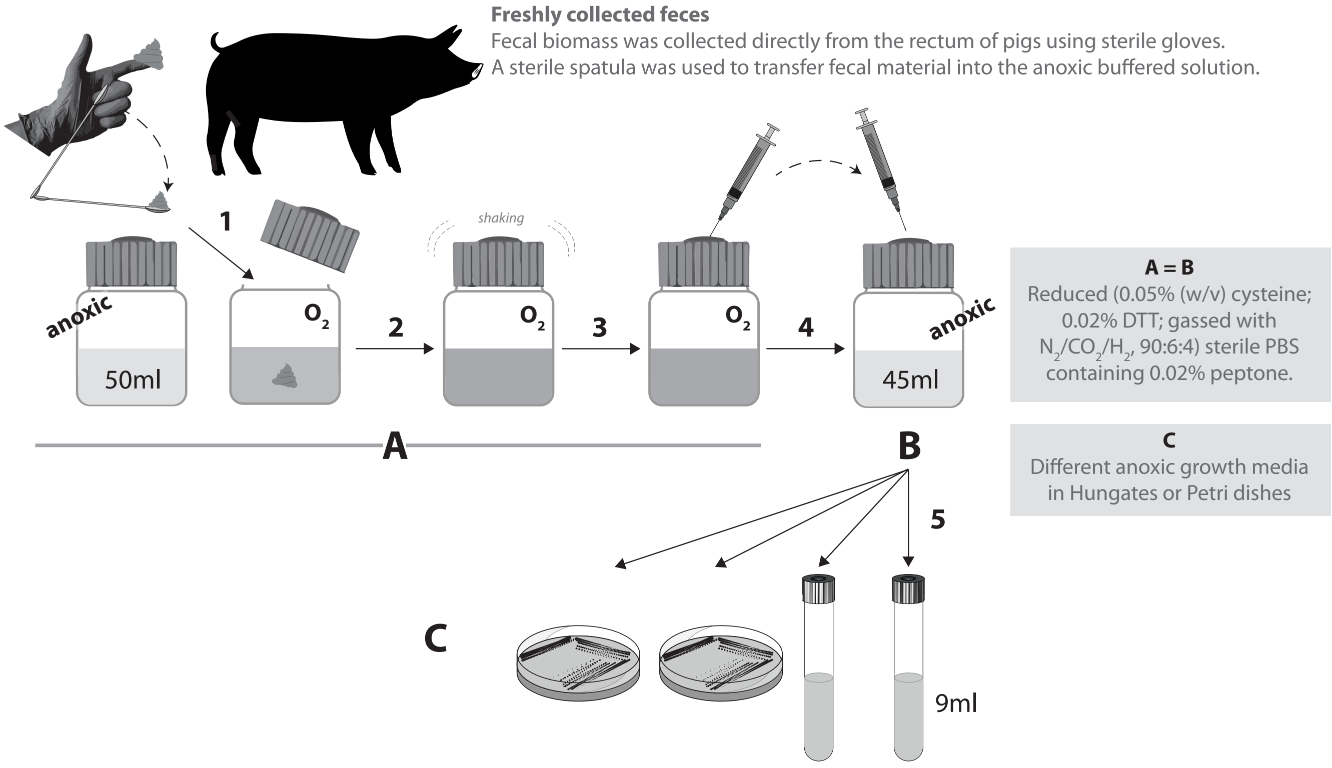

EXPERIMENTAL STEPS

1-- Open the lid of the flask and rapidly transfer ca. 5g of feces using sterile instruments (*e.g.* single-use plastic loops). Thereby, a dilution of ca. *1:10 (w/v)* is obtained.
2-- Close the lid and hand-shake vigourusly until fecal material is re-suspended. Remember, the environment now contains oxygen, so be quick (<1 min).
3-- Let to stand for 1 min to sediment debris. Collect 5 ml of fecal slurry using a syringe with sterile needle (>1 mm ø) via the rubber stopper (avoid particles).
4-- Transfer to new flask through rubber stopper. Thereby, a cumulative dilution of ca. *1:100* is obtained. Mix gently to homogenize.
5-- After transport to the lab, transfer ca. 200 µl of fecal slurry to Hungate tubes using a syringe. Thereby, a final dilution of ca. *1:5,000* is obtained.
Alternatively, dilution series can be plated directly onto agar plates.
In all steps, be mindful of contaminations. For instance, when opening flasks or transferring slurries with syringes through the rubber stoppers.
Flasks and Hungates were autoclaved with aluminium foil over theirs lids for the sake of transportation. These were removed just prior to handling.

Supplementary Fig. 14 / Schematic representation of the fecal sample collection procedure for anaerobic cultivation.

Supplementary References

[1]: Rosero, J. A. et al. Reclassification of Eubacterium rectale (Hauduroy et al. 1937) Prévot 1938 in a new genus Agathobacter gen. nov. as Agathobacter rectalis comb. nov., and description of Agathobacter ruminis sp. nov., isolated from the rumen contents of sheep and cows. IJSEM 66, 768-773, doi:10.1099/i-jsem.0.000788 (2016)
[2]: Zuo, G. & Hao, B. Whole-genome-based phylogeny supports the objections against the reclassification of Eubacterium rectale to Agathobacter rectalis. IJSEM 66, 2451-2451 (2016)
[3]: Holt SC (1978) Anatomy and chemistry of spirochetes. Microbiol reviews 42, 114-160
[4]: Jutras BL, Lochhead RB, Kloos ZA, Biboy J, Strle K, Booth CJ, Bovers SK, Gray J, Schumann P, Vollmer W, BockenStedt LK, Steere AC, Jacobs-Wagner C (2019) *Borrelia burgdorferi* peptidoglycan is a persistent antigen in patients with Lyme arthritis. Proc Natl Acad Scie USA 116, 13498-13507
[5]: Gonzalez-Prendes, R. *et al.* Modulatory Effect of Protein and Carotene Dietary Levels on Pig gut Microbiota. Sci Rep 9, 14582 (2019)
[6]: Blutke, A. *et al.* The Munich MIDY Pig Biobank - A unique resource for studying organ crosstalk in diabetes. Mol Metab 6, 931-940 (2017)
[7]: Reitmeier, S. *et al.* Arrhythmic gut microbiome signatures predict risk of Type 2 diabetes. Cell Host Microbe in press, doi:10.1101/2019.1112.1127.889865 (2020)
[8]: Lagkouvardos, L. *et al.* IMNGS: A comprehensive open resource of processed 16S rRNA microbial profiles for ecology and diversity studies. Sci Rep 6, 33721 (2016)
[9]: Lagkouvardos, L. *et al.* Rhea: a transparent and modular R pipeline for microbial profiling based on 16S rRNA gene amplicons. Peer J 5, e2836 (2017)
