# Supplementary material for: A collection of bacterial isolates from the pig intestine reveals functional and taxonomic diversity
Source: Nat Commun. 2020 Dec 15;11:6389. doi: 10.1038/s41467-020-19929-w (PMC7738495; doi:10.1038/s41467-020-19929-w)
Supplement: Supplementary file 3 — Description of Additional Supplementary Files [file 41467_2020_19929_MOESM3_ESM.pdf]

Description of Additional Supplementary Files

File Name: Supplementary Data 1
Description: Detailed PiBAC strain information

File Name: Supplementary Data 2
Description: Novel taxa with PiBAC against metagenome-reconstructed genomes

File Name: Supplementary Data 3
Description: Genomic and transcriptomic data of C. scindens DSM 100975
