# Supplementary material for: A collection of bacterial isolates from the pig intestine reveals functional and taxonomic diversity
Source: Nat Commun. 2020 Dec 15;11:6389. doi: 10.1038/s41467-020-19929-w (PMC7738495; doi:10.1038/s41467-020-19929-w)
Supplement: Supplementary file 7 — Reporting Summary [file 41467_2020_19929_MOESM7_ESM.pdf]

nature research

Corresponding author(s): Thomas Clavel

Last updated by author(s): Oct 12, 2020

Reporting Summary

Nature Research wishes to improve the reproducibility of the work that we publish. This form provides structure for consistency and transparency in reporting. For further information on Nature Research policies, see our Editorial Policies and the Editorial Policy Checklist.

Statistics

For all statistical analyses, confirm that the following items are present in the figure legend, table legend, main text, or Methods section.

n/a	Confirmed	
☐	☒	The exact sample size (*n*) for each experimental group/condition, given as a discrete number and unit of measurement
☐	☒	A statement on whether measurements were taken from distinct samples or whether the same sample was measured repeatedly
☐	☒	The statistical test(s) used AND whether they are one- or two-sided<br>*Only common tests should be described solely by name; describe more complex techniques in the Methods section.*
☒	☐	A description of all covariates tested
☐	☒	A description of any assumptions or corrections, such as tests of normality and adjustment for multiple comparisons
☐	☒	A full description of the statistical parameters including central tendency (e.g. means) or other basic estimates (e.g. regression coefficient) AND variation (e.g. standard deviation) or associated estimates of uncertainty (e.g. confidence intervals)
☐	☒	For null hypothesis testing, the test statistic (e.g. *F*, *t*, *r*) with confidence intervals, effect sizes, degrees of freedom and *P* value noted<br>*Give P values as exact values whenever suitable.*
☒	☐	For Bayesian analysis, information on the choice of priors and Markov chain Monte Carlo settings
☒	☐	For hierarchical and complex designs, identification of the appropriate level for tests and full reporting of outcomes
☒	☐	Estimates of effect sizes (e.g. Cohen's *d*, Pearson's *r*), indicating how they were calculated

Our web collection on statistics for biologists contains articles on many of the points above.

Software and code

Policy information about availability of computer code

Data collection	Online open source data were used in this work, accessible via: https://www.ebi.ac.uk/ena/data/view/PRJEB11755; https://www.ncbi.nlm.nih.gov/refseq; https://pfam.xfam.org; www.imngs.org; doi:10.5281/zenodo.4075024
Data analysis	The pipelines used in this work can be accessed online, as stated in the manuscript: www.imngs.org; https://github.com/strowig-lab/PIBAC, referenced under doi:10.5281/zenodo.4075065.

For manuscripts utilizing custom algorithms or software that are central to the research but not yet described in published literature, software must be made available to editors and reviewers. We strongly encourage code deposition in a community repository (e.g. GitHub). See the Nature Research guidelines for submitting code & software for further information.

Data

Policy information about availability of data

All manuscripts must include a data availability statement. This statement should provide the following information, where applicable:

- Accession codes, unique identifiers, or web links for publicly available datasets
- A list of figures that have associated raw data
- A description of any restrictions on data availability

All 16S rRNA genes sequences and genomes generated in the present study are available via GenBank using the individual accession numbers provided in Supplementary Data 1. Metagenome-assembled genomes are publicly available at https://github.com/strowig-lab/PIBAC, referenced under doi:10.5281/zenodo.4075024. The 16S rRNA gene amplicon data from pig faeces, the transcriptomic data from in vitro cultures, and all other data from this study are acccessible in NCBI via project PRJNA561470 (https://www.ncbi.nlm.nih.gov/bioproject/561470).

Field-specific reporting

Please select the one below that is the best fit for your research. If you are not sure, read the appropriate sections before making your selection.

- [x] Life sciences
- [ ] Behavioural & social sciences
- [ ] Ecological, evolutionary & environmental sciences

For a reference copy of the document with all sections, see nature.com/documents/nr-reporting-summary-flat.pdf

Life sciences study design

All studies must disclose on these points even when the disclosure is negative.

Sample size	The manuscript includes either data from the literature with previously determined fixed sample size or large-scale analysis of amplicon data using the maximum number of samples (>1,000) available in public databases at the time of analysis.
Data exclusions	No data were excluded.
Replication	Replication is not relevant in the context of this cultivation-based studies. Micrographs were replicates on at least three occasions and in two different labs for electron microscopy, as described in detail in the manuscript
Randomization	Does not apply.
Blinding	Does not apply.

Reporting for specific materials, systems and methods

We require information from authors about some types of materials, experimental systems and methods used in many studies. Here, indicate whether each material, system or method listed is relevant to your study. If you are not sure if a list item applies to your research, read the appropriate section before selecting a response.

Materials & experimental systems

n/a	Involved in the study	
x		Antibodies
x		Eukaryotic cell lines
x		Palaeontology and archaeology
	x	Animals and other organisms
x		Human research participants
x		Clinical data
x		Dual use research of concern

Methods

n/a	Involved in the study	
x		ChIP-seq
x		Flow cytometry
x		MRI-based neuroimaging

Animals and other organisms

Policy information about studies involving animals; ARRIVE guidelines recommended for reporting animal research

Laboratory animals	Laboratory pigs were used as donors of stool material for bacterial isolation. Animal use was approved by the Federal Government of Bavaria (approval no. ROB55.2-2-2532.Vet_02-18-33, 29).
Wild animals	Fecal samples were collected from farmed 'wild' boars, i.e., living in a free-range enclosure and left to forage, breed, and raise their young with minimal human intervention. Permission of the farmer was asked prior to sampling. Defecation was watched from a safe distance, and droppings were collected from three separate boars once they had moved away, and within 10 minutes of voiding. Fecal samples were immediately placed in sterile sample collection bags, and on to ice packs, transferred to the laboratory and stored at -80°C.
Field-collected samples	This study did not involve field-collected samples.
Ethics oversight	Federal Government of Bavaria, Germany; National Animal Disease Center Animal Care and Use Committee, USA.

Note that full information on the approval of the study protocol must also be provided in the manuscript.
