# Supplementary material for: A collection of bacterial isolates from the pig intestine reveals functional and taxonomic diversity
Source: Nat Commun. 2020 Dec 15;11:6389. doi: 10.1038/s41467-020-19929-w (PMC7738495; doi:10.1038/s41467-020-19929-w)
Supplement: Supplementary file 2 — Peer Review File [file 41467_2020_19929_MOESM2_ESM.pdf]

Editorial Note: This manuscript has been previously reviewed at another journal that is not operating a transparent peer review scheme. The manuscript was considered suitable for publication without further review at Nature Communications.
